# Early Domestication History of Asian Rice Revealed by Mutations and Genome-Wide Analysis of Gene Genealogies

**DOI:** 10.1186/s12284-022-00556-6

**Published:** 2022-02-15

**Authors:** Yingqing Lu, Yunzhang Xu, Nan Li

**Affiliations:** 1 State Key Laboratory of Systematic and Evolutionary Botany, Institute of Botany, Chinese Academy of Sciences, 20 Nan Xin Cun, Beijing, 100093 China; 2 University of Chinese Academy of Sciences, Beijing, 100049 China; 3 Present Address: College of Agriculture and Animal Husbandry, Qinghai University, Xining, 810016 China

**Keywords:** 5′ Genealogy, Coding genealogy, Early mutations, Positive selection, New alleles, Domestication genes, Domesticated traits, Gene type, Hybrid origin, Asian rice

## Abstract

**Background:**

Asian rice (O*ryza sativa* L.) has been a model plant but its cultivation history is inadequately understood, and its origin still under debate. Several enigmas remain, including how this annual crop shifted its growth habit from its perennial ancestor, *O*. *rufipogon*, why genetic divergence between *indica* and *japonica* appears older than the history of human domestication, and why some domestication genes do not show signals of introgression between subgroups. Addressing these issues may benefit both basic research and rice breeding.

**Results:**

Gene genealogy-based mutation (GGM) analysis shows that history of Asian rice is divided into two phases (Phase I and II) of about equal lengths. Mutations occurred earlier than the partition of *indica* and *japonica* to *Os* genome mark Phase-I period. We diagnosed 91 such mutations among 101 genes sampled across 12 chromosomes of Asian rice and its wild relatives. Positive selection, detected more at 5′ regions than at coding regions of some of the genes, involved 22 loci (e.g., *An-1*, *SH4*, *Rc*, *Hd3a*, *GL3.2*, *OsMYB3*, *OsDFR*, and *OsMYB15*), which affected traits from easy harvesting, grain color, flowering time, productivity, to likely taste and tolerance. Phase-I mutations of *OsMYB3*, *OsHd3a* and *OsDFR* were experimentally tested and all caused enhanced functions of the genes in vivo. Phase-II period features separate cultivations, lineage-specific selection, and expanded domestication to more genes. Further genomic analysis, along with phenotypic comparisons, indicates that *O. sativa* is hybrid progeny of *O. rufipogon* and *O. nivara*, inherited slightly more genes of *O. rufipogon*. Congruently, modern alleles of the sampled genes are approximately 6% ancient, 38% uni-specific, 40% bi-specific (mixed), and 15% new after accumulating significant mutations. Results of sequencing surveys across modern cultivars/landraces indicate locus-specific usages of various alleles while confirming the associated mutations.

**Conclusions:**

Asian rice was initially domesticated as one crop and later separate selection mediated by human resulted in its major subgroups. This history and the hybrid origin well explain previous puzzles. Positive selection, particularly in 5′ regions, was the major force underlying trait domestication. Locus-specific domestication can be characterized and the result may facilitate breeders in developing better rice varieties in future.

**Supplementary Information:**

The online version contains supplementary material available at 10.1186/s12284-022-00556-6.

## Background

Early agricultural varieties represent a major milestone of human civilization (Diamond 2002), but phenotypes and genotypes of which are generally little known in the absence of historical records or archeological materials. Inferring historical changes from the standing genetic variation has been challenging, as cumulative results of past mutations, segregation/recombination, drift, and selection can be difficult to tease apart according to the causes. Much effort has been put to describe relationships among homologous haplotypes in phylogenetic trees (Felsenstein 1988) or cladograms using the cladistic principal and statistical tests (Posada et al. 2006; Templeton et al. 1992), but these methods do not show directly the temporal distribution of individual mutations on a gene genealogy, as their major issue is on estimation of degrees of relatedness of biological groups. We argue that knowing mutational distribution on a gene genealogy is more relevant to addressing issues on domestication than knowing degrees of closeness of the groups.

Issues of domestication of a crop typically include its origin, selection, selected traits, and cultivation history, and much of the information has been embedded in the temporal distributions of past mutations across genomic regions. Early mutations, in particular, can indicate the nature of historical selection for crops of long histories. Knowledge of the mutations can also help assess how a subset of genetic variation of the wild predecessor was retained in the successor crops (Doebley et al. 2006). During domestication, phenotypic differentiations are accelerated by human selection and the underlined mutation patterns inevitably left prints in genomes of crops in forms of selective sweep (Olsen et al. 2006; Sweeney et al. 2007), or/and positive selection (Koenig et al. 2013; Rubin et al. 2012), which can be readily detected. Nonetheless, duration of detected selection has rarely been articulated.

Since early mutations must have been frequent enough to transcend domestication history, there is a good chance for them to be recognized in a small number of high-quality genomes. This notion is supported by a recent finding that a high percentage (98%) of common alleles has been shared between wild and domesticated populations of strawberry (Hardigan et al. 2021). With increasingly known genomes of wild relatives of crops, gene genealogies can be constructed at genomic level to help grasp a full picture of past variation. Here we show that early mutations can be diagnosed in Asian rice (*Oryza sativa* L.).

Asian rice has been well sequenced, but its origin is still in debate after intensive studies in the recent decade (Choi et al. 2017; Civan and Brown 2018; Gross and Zhao 2014; Huang and Han 2016; Huang et al. 2012; Ishikawa et al. 2020). Its closest wild relatives are the tall, aquatic perennial, *O. rufipogon* Griff. (*Or*), and the short, drought resistant annual, *O. nivara* Sharma & Shastry (*On*). The perennial has a mixed-mating system, and the annual performs self-fertilization and has been considered derived from the perennial (Chang 1976). The wild species can interbreed with each other (Oka 1974) and with Asian rice (Thomson et al. 2003; Mahmoud et al. 2008). Within Asian rice, two traditional subspecies, indica (*O*. *sativa* ssp. *indica*) and japonica (*O*. *sativa* ssp. *japonica*), were further divided into subgroups. Japonica rice (sensu lato) refers to varieties of *temperate japonica*, *tropical japonica*, and *aromatic* subgroups, whereas indica rice (sensu lato) includes those of *indica* (sensu stricto) and *aus* subgroups (Garris et al. 2005). The origins of indica and japonica are also in debate. Ecologically, cultivars of japonica can grow in regions broader than those of indica (Khush 1997). The two subspecies differ in physiological traits (Oka and Morishima 1982) and genomic expression (Liu et al. 2007), but culinary differences are the most familiar– rice from indica is less sticky than that of japonica and easily solidifies at a low temperature.

The origin of Asian rice has been hypothesized in various forms. Patterns of phylogeography (Londo et al. 2006; Gutaker et al. 2020) and population structure (Civan et al. 2015) support multiple domestications, but trait analysis (Oka and Morishima 1982), genetic variations in microsatellite patterns (Gao and Innan 2008), phylogenetic analysis (Molina et al. 2011), and genomic variation (Huang et al. 2012) generally support a single domestication, with *O. rufipogon* frequently taken as the immediate ancestor of *O. sativa*. Meanwhile, distribution of retroposon p-SINE1 across accessions suggests close associations of *O. nivara* with *O. sativa* indica and *O. rufipogon* with *O. sativa* japonica (Cheng et al. 2003). Some of the hypotheses can be tested with patterns in mutation distributions. For instance, Huang et al. (2012) proposed that the ancient Asian rice (early japonica) could first come from *O*. *rufipogon* and then cross with *O*. *nivara* to form indica. If it is true, no genetic signatures of *O*. *nivara* are to be found in genomes of japonica. The prediction can be addressed in genomic analysis, but no relevant report has been seen in the literature.

Other puzzles about Asian rice (Sang and Ge 2007) also persist. One is that nuclear divergence between indica and japonica is older than the history of human domestication itself (Ma and Bennetzen 2004). This divergence is also seen in genomes of chloroplasts (Tong et al. 2016) and mitochondria (Cheng et al. 2019). The second one is the lack of introgressions for major domestication genes such as *SH4* and *Rc* (Civan and Brown 2018). The third puzzle is the annual habit of rice, which cannot be generated from a cross between indica and *O*. *rufipogon* (Xiong et al. 1999) or between *O. nivara* and *O*. *rufipogon* in F2 generation (Grillo et al. 2009), as the perennial growth is dominant in F1 and appears persistent in F2 generation as well.

In this study, we tried to address the above conflicts by reconstructing mutation-based gene genealogies, focusing on identification of early mutations and evaluation of their functions. The result and associated tests help dissipate the previous dilemma, broaden the scope of domestication genes, and shed light on the early domestication history as well as the later selection in Asian rice. More importantly, the origin of Asian rice is clarified with little ambiguity for the first time.

## Results

### Early Mutations Show Pervasive Positive Selection During Early Domestication

**Fig. 1:**
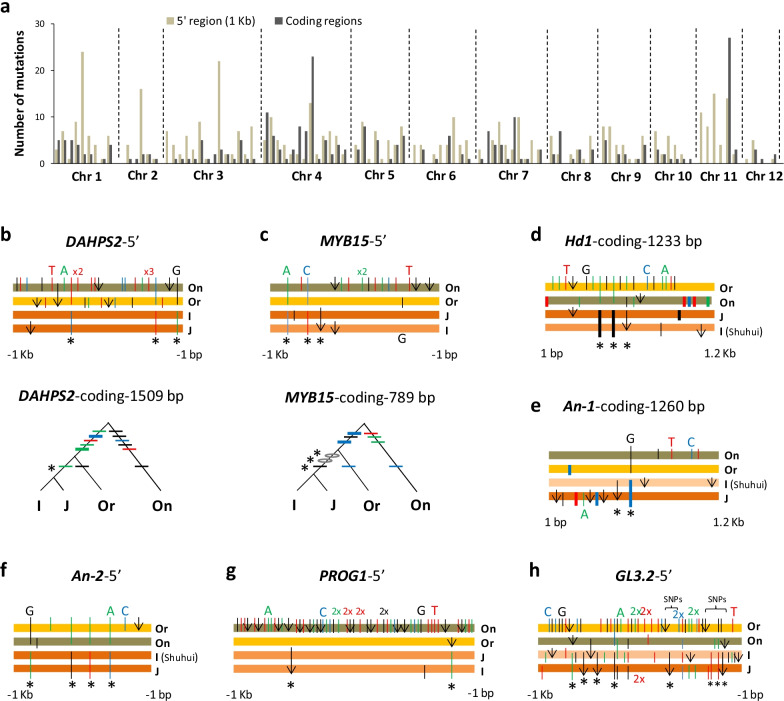
Identification of early mutations in *O. sativa*. **a** Manhattan plot of 756 *Os* mutations on 101 genes distributed across rice chromosomes (chr1–chr12). Details on sampled genes see Additional file 1: Table S1. **b**–**h** Gene genealogies of seven genes showing early mutations (in asterisks). Gene genealogies connecting indica (I) and japonica (J) of *O*. *sativa* to its wild relatives (*O. rufipogon* (Or) and *O. nivara* (On)) are shown in bar graph or tree. A graph of horizontal bars shows orthologs in bars and similarities in similar colors, with substitutions coded by colored sticks (A in green, T in red, C in blue, and G in black, and thick ones for nonsynonymous changes) and indels by arrows. A tree-like gene genealogy also uses colored sticks as above for substitutions but circles for indels. Aligned sticks refer to changes at the same nucleotide site. Number of repeated and adjacent changes is shown before x for simplicity. Allele of indica (I) is shown as the identical sequence among three indica genomes or for genome in the parentheses (Shuhui for Shuhui-498); allele of japonica (J) is from the japonica genome. Only the genic regions having early mutations are shown here

We took members of two major subgroups (*indica* and *temperate japonica*) as representatives of indica and japonica of *O*. *sativa*, showing, from five high-quality genomes including those of the closest wild species (*O. nivara* and *O*. *rufipogon*), relationships of orthologs of each sampled gene by two gene genealogies — one based on 5′-sequences and the other on coding-sequences — to capture possibly varying signals of selection across genic regions. A total of 101 genes (Additional file 1: Table S1) sampled in parallel from these genomes across all chromosomes gave rise to 202 gene genealogies (Additional file 2: Fig. S1a–l). The topology of each gene genealogy, based on carefully aligned orthologous sequences, is determined by polymorphisms of oldest mutations including both substitutions and insertion/deletions (indels). A total of 756 mutations are present in the sampled genes of *O. sativa* (*Os* mutations), of which 478 dispersed in the 1 Kb 5′ regions and 278 scattered in exon regions of various lengths (Fig. 1a). Significantly, 91 early *Os* mutations accumulated at 30 loci in the lineage prior to the partition of indica and japonica but after the divergence of *O. nivara* from *O. rufipogon* (Additional file 2: Fig. S1, Additional file 3: Table S2), which we call the early phase (Phase I) of domestication.

To detect signals of positive selection in 5′ regions during Phase-I domestication, we examined the mutations occurred to *Os* introns within the same period (Additional file 4: Table S3). The intronic mutational rate (0.56 per kilo-nucleotides per period), taken as a proxy of background mutation rate of the *Os* genomes, was used to establish the threshold of two mutations over a 1 K-nucleotide 5′ region during the period as the signal of positive selection. Under the criteria, 5′ regions of 18 *Os* loci were under positive selection (Table 1). For coding regions, 8 genes (4 overlapping with ones involved also in the 5′ selection) displayed positive selection under the stringent test of *d*_*N*_/*d*_*S*_ > 1 (Nielsen and Yang 1998) or amino acid changes of more than three-fold the background mutation for cases of *d*_*S*_ being zero and/or indels. The analysis generated a conserved estimate of 22 *Os* loci under early positive selection, which involved at least six categories of traits (Table 1).

**Table 1:** Sampled *O. sativa* loci under positive selection during the early domestication period

Trait category	Locus^d^	5′ mutations^a^	Coding mutations^a^^,^^b^	References
Grain shattering/awn length	***SH4***	6(1 idl)	1(nons)^c^	Li et al. (2006b)
	*An-1(Awn-1)*		2(1non, 1 idl:1 aa)	Luo et al. (2013)
	*An-2(Awn-2)*	4		Gu et al. (2015)
	*RAE2*	3(1 idl)	1(idl:stop codon)	Wang et al. (1995)
Grain taste/color	*C1*	2(1 idl)		Reddy et al. (1998)
	***DFR***	2(1 idl)		Furukawa et al. (2007)
	***Rc***	3(1 idl)	2(1 nons, 1 idl:stop codon)	Sweeney et al. (2006)
	***MYB3***		2(1 nons, 1 idl:3 aa)	This study
Grain productivity	*GIF1*	2(idl)	2(2 nons)	Wang et al. (2008)
	*GL3.2*	7(3 idl)	3(1 nons)	Xu et al. (2015)
	*AGO2*	3(3 idl)	1(1 nons)	Yin et al. (2020)
Flowering time	*Hd1*	1	3(2 nons,1 idl:2 aa)	Yano et al. (2000)
	*Hd6*	2(1 idl)		Takahashi et al. (2001)
	***Hd3a***	4(1 idl)		Kojima et al. (2002)
Tolerance/resistance	*EPSPS*	3(1 idl)		Xu et al. (2002)
	*MYB15*	3(1 idl)	3(2 idl:5 aa)	This study
	*AGO2*	3(3 idl)	1(1 nons)	Yin et al. (2020)
	*DAHPS2*	3	1	This study
Growth	*PROG1*	2(1 idl)	1	Jin et al. (2008)
	*TCP19*	2(1 idl)		Liu et al. (2021b)
Unknown	*ME*	3	1(idl:2 aa)	This study
	*Os04g47040*		1(idl:5 aa)	This study
	*Os12g34860.1*		2(1 nons, 1 idl:stop codon)	This study

^a^Number of mutations includes those of substitutions (synonymous or nonsynonymous (nons) for coding regions) and indels (idl). Only nons and idl are listed for simplicity. Amino acid (aa) changes introduced by each indel was listed after comma

^b^All mutations are listed but only those underlined were considered under positive selection

^c^This mutation was experimentally confirmed by Li et al. (2006b)

^d^Loci in bold have selected mutation(s) tested functional in at least one experiment

### Agriculture Traits, Tolerance, and Flowering Time were Targeted During the Early Domestication

The inclusion of known domestication genes (e.g., *SH4*, *Rc*), which served as internal references for the detective method above, indicates that the positively selected loci are candidates of domestication genes. Most loci on the list are previously unknown of their roles in domestication. For instance, three genes of the shikimate pathway were under positive selection, including 5′ regions of *OsEPSPS*, which encodes 5-enolpyruvylshikimate 3-phosphate synthase (Xu et al. 2002; Zhou et al. 2006), and *OsDAHPS2* (Fig. 1b), which presumably encodes 3-deoxy-7-phosphoheptulonate synthase, and both 5′ and coding regions of *OsMYB15* (Fig. 1c), a homolog of which in *Arabidopsis thaliana*, *AtMYB15*, may regulate the shikimate synthesis (Chen et al. 2006) while affecting cold tolerance (Agarwal et al. 2006) and immunity (Chezem et al. 2017). Positively selected mutations at the shikimate loci could have altered the tolerance of early Asian rice. Genes associated with the anthocyanin pathway were also positively selected—one encoding dihydroflavonol 4-reductase named *OsDFR* (Shih et al. 2008) and the other a regulatory gene *OsC1* (Zheng et al. 2019). The two loci influence synthesis of anthocyanins and possibly other classes of flavonoids. Since content change of phenylpropanoids may influence flavors of cooked rice (Bett-Garber et al. 2013), mutations at *OsDFR* could affect the taste of early Asian rice. Given that shikimate pathway supplies precursors for biosynthesis of aromatic amino acids and flavonoids (reviewed by Maeda and Dudareva (2012)), mutated enzymes of the two pathways above or their altered expressions could bring correlated changes on traits of tolerance, taste, and nutrition of early Asian rice.

Three flowering-time genes, *Hd1* in the coding regions (Fig. 1d) and *Hd6* (Additional file 2:Fig. S1c) and *Hd3a* (Additional file 2: Fig. S1f) in their 5′ regions, were under early positive selection. So were genes for awn development, such as *An-1* (Fig. 1e) and *Rae2* (Additional file 2: Fig. S1h), and for awn-length, such as *An-2* (Fig. 1f). Early positive selection detected on the 5′ region of *PROG1* (Fig. 1g), which affects plant architecture (Jin et al. 2008), clarified its role in domestication, since previously suspected A → T substitution in coding is shared between *O. nivara* and *O. sativa* thus unlikely the target of human selection*.* Early positive selection also strongly acted on productivity genes such as *GL3.2* (Fig. 1h). Only continuous selection on the same materials would result in the fixation and accumulation of the mutations and documented positive selection involved agriculture-related traits, we thus conclude that the positive selection was largely the result of human selection during this early period of domestication.

Early mutations identified for known domestication genes may explain their lack of introgressions between subgroups. *SH4* is a key gene for grain shattering (Li et al. 2006b) with the non-shattering mutant allele (*sh4*) nearly fixed in the modern rice (Zhang et al. 2009). Its lysine-to-asparagine substitution was demonstrated to affect the shattering phenotype (Li et al. 2006b). We show that this substitution occurred during the early domestication (Additional file 2: Fig. S1d), along with six 5′ mutations fixed in the same period. *Rc*, a domestication gene related to grain-color (Sweeney et al. 2007, 2006), was also intensely selected during the early period in both coding and 5′ regions (Additional file 2: Fig. S1g). Along with other 20 *Os* loci, a conserved estimate of 54 mutations in 5′ regions and 17 in the coding regions have been accumulated under positive selection during the early period (Table 1). These results are consistent with the existence of one crop and its continuous human selection. Validity of identified mutations was subsequently tested for their functional impacts at some newly recognized loci of domestication.

### Positive Selection on Anthocyanin- and Flowering-Related Genes were Experimentally Supported

**Fig. 2:**
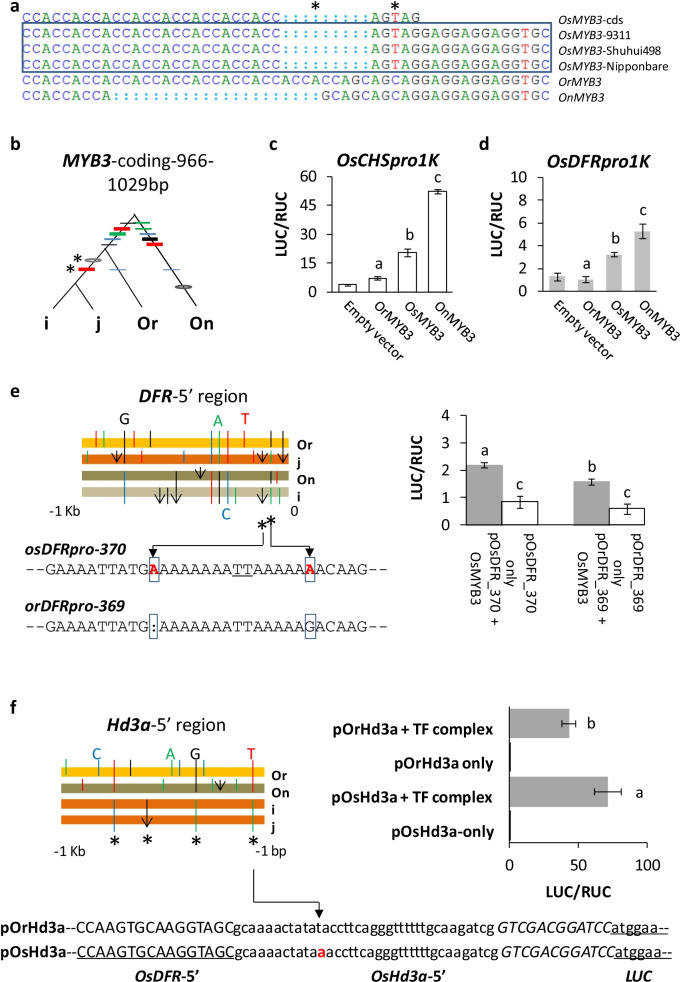
Functional analysis of early mutations. **a** Partial alignment of *Oryza MYB3* exon3s. Early mutations are by the asterisks. **b** Gene genealogy based on the entire coding regions of *MYB3*. The format follows Fig. 1. **c** Activation capacities by three MYB3s on the 1 Kb-long Nipponbare promoter of *OsCHS* (*OsCHS*_*pro1K*_)*.* The promoter activity was measured by the ratio of two fluorescent luciferases (LUC/RUC) in three biological replicates (n = 3, but 5 for the negative control). A significant *t*-test (*P* < 0.001) on mean difference was shown by different letters. Data are represented as mean ± SE here and below. **d** Activations of *MYB3*s on the 1041 bp-long Nipponbare promoter of *OsDFR* (*OsDFR*_*pro1K*_)*.* Each test had six biological replicates (four for negative control). Comparisons of averages by different letters were significant (*t*-test, *P* < 0.008 in both cases). **e** Impact of two early mutations (in square; comma refers to an indel) within 5′ region of *OsDFR.* Each test of transient expression had six biological replicates (four for negative control). Treatment comparisons in different letters were significant (*t*-tests, all *P* < 0.003). **f** Impact of one substitution (by the folded arrow and red letter) in the 5′ UTR of *Hd3a*. The lower panel shows the synthetic 5′UTR, with partial 5′UTR of *OsDFR* promoter (upper case and underlined) replaced by the 5′ UTR fragment (lower case) of *Hd3a*. The upper and italic sequences indicate restriction enzymes between the reporter and the tested 5′UTR region. The right panel shows reporter activities driven by OsC1/OsB2/OsTTG1 (TF complex) and the negative controls (n = 3). The mean difference is supported by a significant *t*-test (*P* < 0.001)

Mutations at three loci were evaluated for their possible impacts on gene function. The first case was early mutations in the coding sequence of *OsMYB3,* a gene that may affect color of seeds (Zheng et al. 2021). Two mutations introduced an early stop codon in the last exon (Fig. 2a), making OsMYB3 shorter than otherwise identical OrMYB3 (Fig. 2b). We investigated the capacity of OsMYB3 as a single regulator, using one Kb-long 5′ regions of chalcone-synthase encoding *OsCHS* and *OsDFR* (Shih et al. 2008) as baits. The results indicate that OsMYB3 not only *trans*-activated *OsCHS* (Fig. 2c) and *OsDFR* (Fig. 2d) but also gained a regulatory capacity stronger than that of its ancestor, OrMYB3 from *O*. *rufipogon*.

The second test was on early mutations in the 5′ region of *OsDFR*. Two mutations—one indel and one substitution—about 370 bp upstream the translation-starting site set *OsDFR*_*pro370*_ apart from *OrDFR*_*pro369*_ (Fig. 2e). When driven by OsMYB3 in vivo, *OsDFR*_*pro370*_ caused a significantly more transcription of the reporter gene than *OrDFR*_*pro369*_ did (Fig. 2e), indicating enhanced gene expression due to the mutations.

The third test was on an early substitution in 5′ UTR of *Hd3a* (Fig. 2f), a homolog of Flowering Locus T gene of *Arabidopsis* (Kojima et al. 2002). The substitution led to 65% more transcripts for *OsHd3a* than those for *OrHd3a* under the same regulatory complex of OsC1/OsB2/OsTTG1 (Fig. 2f). Since elevated OsHd3a could promote early flowering in rice (Kojima et al. 2002; Takahashi et al. 2009), the mutant carrier could set mature seeds earlier and became an easy target of human in grain harvesting. All three cases suggest that the detected positive selection on these gene regions were due to their functional impacts, which were noticed and selected by early breeders.

### Two-Phased History of Rice Domestication

Phase-II domestication of rice began when different mutations started to accumulate in indica or japonica lineage (branch) on a gene genealogy. Of all positively selected 22 *Os* loci of Phase I (Table 1), 14 loci were continually under positive selection, sometimes variably, along lineages of indica and japonica in Phase II (Additional file 3: Table S2). For instance, *An-1* underwent a stronger selection in japonica than in indica or Phase I (Fig. 1e). Meanwhile, positive selection specific to Phase II was observed at 5′ or coding regions of additional 18 loci of indica or japonica genomes (Table 2). Six of the loci appeared to be associated with yield, including grain-filling- and grain-size-related *GS5*, grain-length-related *GL6*, grain-width-related *SPL16,* grain-number-related *NOG1*, and yield-related *SD1* and *Dst*. Two additional flowering-time loci, *Ehd1* and *Hd5*, were positively selected. Six loci experienced positive selection only in indica while other seven loci only in japonica (Table 2). The lineage-specific selection is seen also at unreported or less studied loci, from chorismate-mutase-encoding *OsCM3* (Additional file 2: Fig. S1a), to alcohol dehydrogenase 2-encoding *OsADH2* and unknown (Additional file 2: Fig. S1k). These non-overlapping loci between early and later lineages and between indica and japonica signify that not only selection in phase II differed from that of Phase I and within Phase II, selection also varied between indica and japonica. A major shift of rice cultivation and domestication occurred in Phase II.

**Table 2:** Sampled *Oryza sativa* loci under positive selection during later domestication only

Trait category	Locus^c^	5′ Mutations^a^	Coding mutations^b^	References
Yield	*SD1* in *japonica*	5	2(27 aa)	Ashikari et al. (2002)
	*GS5*	7 in *indica*; 2 in *japonica*	4(3 aa) in *indica*; 3(3 aa) in *japonica*	Li et al. (2011)
	*Dst* in *japonica*	5	5(7 aa)	Li et al. (2013)
	*SPL16* in *japonica*	3		Wang et al. (2015)
	*NOG1* in *japonica*	26		Huo et al. (2017)
	*GL6* in *indica*	5		Wang et al. (2019)
Flowering time	*Ehd1* in *indica*	6		Doi et al. (2004)
	*Hd5*	4 in *indica*; 2 in *japonica*	1(1 aa) in *indica*	Fujino et al. (2013)
Grain shattering	*SSH1* in *japonica*	8	1(3 aa)	Jiang et al. (2019)
Grain texture	***Chalk5*** in *indica*	4	3(1 aa)	Li et al. (2014)
Starch	*bZIP58* (*RISBZ1*)		5(4 aa) in *indica*; 2(2 aa) in *japonica*	Onodera et al. (2001)
Unclassified	*B1*	1 in *japonica*	3(3 aa) in *japonica*; early stop codon in *indica*	Sakamoto et al. (2001)
	*B2* in *indica*	2	3(5 aa)	Sakamoto et al. (2001)
	*CM3* in *indica*	> 6		This study
	*ADH2* in *japonica*	14		This study
	*PK1* in *japonica*	7		This study
	*DAHPS1* in *indica*	9	4(3 aa)	This study
	*Os11g29400.1*	7 in *indica*; 7 in *japonica*	1(1 aa) in *indica*; 26(2 aa) in *japonica*	This study

^a^Mutations shown include both substitutions and indels

^b^All mutations are listed but only those underlined were considered under positive selection. Amino acid (aa) changes introduced by the mutations are in the parentheses

^c^Locus in bold has selected mutation(s) tested functional in at least one experiment

The shift of selection is particularly illuminating in the case of flowering-time gene *Hd3a*. The Phase-I selection occurred only at the 5′ region, which presumably affected only the gene expression (Fig. 2f), but Phase-II selection switched to the coding regions in japonica, leaving indica keeping the protein sequence identical to that of the early rice (Additional file 2: Fig. S1f). An opposite example is *Chalk5*, which controls chalkiness of the endosperm (Li et al. 2014) and has not changed from Phase I to japonica lineage but accumulated four mutations in the 5′ region and three in the coding regions of the gene in indica (Additional file 2:Fig. S1e).

Besides positive selection, human influence sometimes led to negative selection. One candidate example is the 5′ region of *OsC1,* which had two early mutations during Phase I but none for Phase II. We examined this peculiar pattern by sequencing 5′ regions of additional cultivars, including Heidao of *temperate japonica* subgroup and Jixuenuo of *indica* subgroup, and obtained the same results. It appears that after positive selection of Phase I, further changes in expression of *OsC1* were discouraged during Phase-II domestication, which suggests negative selection. Similar deficiency of mutations in the phase II period was seen also at the coding regions of *OsMYB3* (Additional file 2: Fig. S1c), *OsMYB15* (Additional file 2: Fig. S1d), and *OsPROG1* (Additional file 2: Fig. S1g).

### The Emergence Time of Japonica and Indica

The annual growth habit and presumably uninterrupted planting history of Asian rice could bring about somewhat steady selection under traditional breeding, as a more or less fixed proportion of the grains were selected each year for the next generation. To estimate roughly the relative durations of Phase I and Phase II, we sampled the *Os* loci that showed mutations throughout rice history (Additional file 3: Table S2), with an exception of *AGO2*, a gene affecting grain length and salt tolerance (Yin et al. 2020) but considered an outlier here due to its disproportional influence on the total number of mutations in Phase II. The remaining 29 loci accumulated 87 mutations in the early period and 88 in the lineage of indica and 83 in the lineage of japonica (Additional file 3: Table S2) at 5′ regions. Using our model-based estimation method (see Methods) and taking the sum of mutations observed on the two continuing lineages (after the separation of *O*. *sativa* from its closest ancestor on a gene genealogy) as a measure of the entire history of rice domestication, the estimates show that Phase I constitutes approximately half of the domestication history (49.7% by the lineage of indica or 51.2% by the lineage of japonica). Estimates based on only mutations in the coding regions gave similar results, ranging from 47.0 to 49.2%. Consistently, the number of positively selected loci (22) in Phase I was close to those (22 in japonica and 21 in indica) in Phase II, based on our sampled genes. Collectively, the estimates indicate that the history of rice domestication can be divided into Phase I and II almost evenly in time.

### O. sativa was Originated from Hybridization Between O. nivara and O. rufipogon

**Fig. 3:**
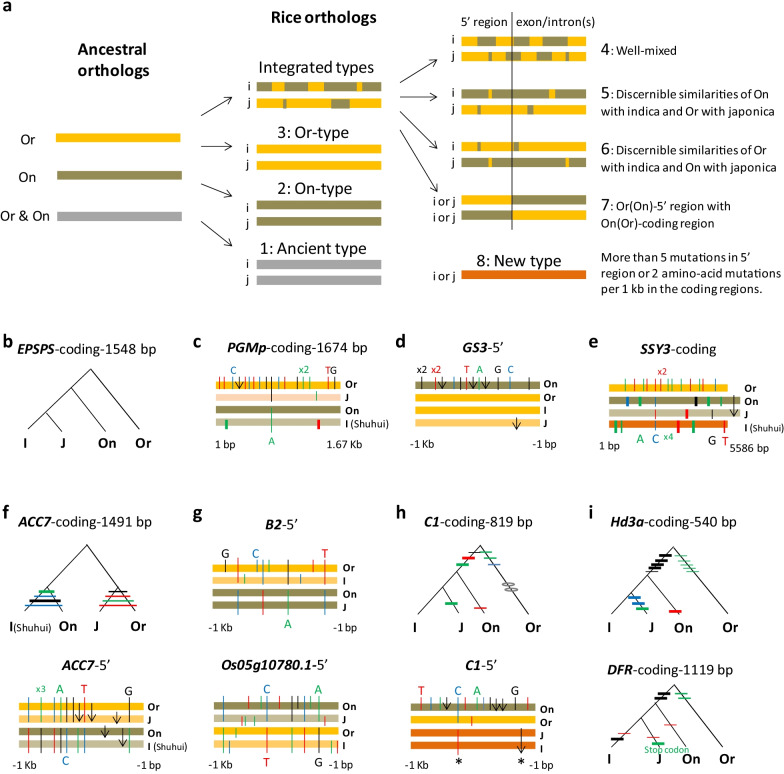
Types of genes in *O. sativa.* **a** A flow chart showing the movement of parental genes into genomes of *O. sativa* and eight gene types in standing rice*.* The left panel classifies the parental orthologs into Or (*O*. *rufipogon*, in yellow) and On (*O*. *nivara*, in dark green) and their common ones (in dark red). The middle and right panels show the current types of rice genes observed in genomes of indica (i) and japonica (j), with the integrated types further broken down into four classes, along with new alleles, in the right panel. **b** A genealogy of Type 1 gene. The example by *EPSPS* of the shikimate pathway shows no changes. **c** A genealogy of Type 2 gene. A resemblance to *On* sequence is seen at *PGMp* of glycolysis. **d** A genealogy of Type 3 gene. A resemblance to *Or* sequence is shown by *GS3*, a regulator of grain size. **e** A genealogy of Type 4 gene. The well-mixed type is found in the coding regions of *SSY3* in starch synthesis. **f** Type-5 gene genealogies with Or-j and On-i associations. It appears in both 5′ and coding regions of *ACC7*, a gene likely involved in ethylene synthesis. **g** Type-6 gene genealogies with Or-i and On-j associations. 5′ regions of two genes, *B2* (a regulator on the anthocyanin pathway) and an unknown gene, show the associations. **h** Genealogies of a Type 7 gene. Different origins of 5′ and coding regions is at *C1*. **i** Genealogies of Type 8 genes. Mutations causing multiple amino-acid changes in *Hd3a* led to the Nipponbare allele of *Hd3a*. And an early stop codon led to a new allele of *OsDFR* in *japonica* lineage. Gene genealogies follow the format of Fig. 1

Genome-wide sampling of genealogies indicates that some *Os* loci are closer to the orthologs of *O*. *nivara* and others to those of *O. rufipogon* (Additional file 2: Fig. S1). Further pursuit of the affinities led to the finding of eight types of *Os* genes across 12 chromosomes (Fig. 3a; Additional file 5: Table S4). Type 1 designates ancient genes (Fig. 3b) and their slight variants in *O*. *sativa*. Nine genes have identical protein sequences and four have nearly unchanged amino-acid sequences among all four *Oryza* groups. These are the old and conserved genes in the *Oryza* groups. Type 2 is an *On*-like gene, as exemplified by *PGMp* (Fig. 3c), which encodes a plastidic form of phosphoglucomutase. 16 *Os* regions at 12 *Os* loci have sequences of 5′ or/and coding regions identical or similar to *O. nivara*. Type 3 is a discernibly *Or*-like gene and found in 30 *Os* regions involving 18 *Os* loci, as shown by *OsGS3* (Fig. 3d), a gene involved in grain size (Fan et al. 2006)*.* Type 4 is for genes of well mixed sequences (Fig. 3e), found at 79 rice regions (involving 56 *Os* loci) showing both features of *O. rufipogon* and *O. nivara*. Genes of Type 5 show associations between *Or* and japonica orthologs and between *On* and indica orthologs (Fig. 3f), as displayed at 29 *Os* regions (involving 20 *Os* loci) where japonica is closer to *O. rufipogon* and indica to *O. nivara*. Type 6 exhibits the reversal of Type 5, showing *Or*-indica association and *On*-japonica association instead, as seen at the 5′ regions of two loci, one is a regulatory bHLH gene *B2* and the other unknown (Fig. 3g). Type 7 is unique in having separate origins between the 5′ and coding regions, as shown by *OsC1*, which has an *Or*-like 5′ region but *On*-like coding regions (Fig. 3h). Type 8 refers to new variants, when allelic mutations outnumbered five in 5′ region or two in coding region in *O. sativa*, as seen at *OsHd3a* and *OsDFR* of *japonica* (Fig. 3i). New alleles emerged in 31 cases among sampled genes, involving 24 *Os* loci (*CKX2* (Ashikari et al. 2005), *NOG1*, *CM3*, *SD1*, *SK2*, *DAHPS1*, *GL3.2*, *Dst*, *An-1*, *B2*, *AGO2*, *F3H*, *GS5*, *EPSPS*, *Hd3a*, *Hd1*, *GL6*, *bZIP58*, *SSH1*, *RAE2*, *Ehd1*, *ADH2*) and three unknowns (Additional file 1: Table S1). All of the loci were under positive selection. Together, these *Os* gene types suggest contributions of both *O. rufipogon* and *O. nivara* to the rice genomes, arguing strongly for a hybrid origin of rice. Consistent with the origin, the most frequently observed gene type, the mixed-type, disperses across all rice chromosomes (Additional file 1: Table S1 and Additional file 5: Table S4).

**Fig. 4:**
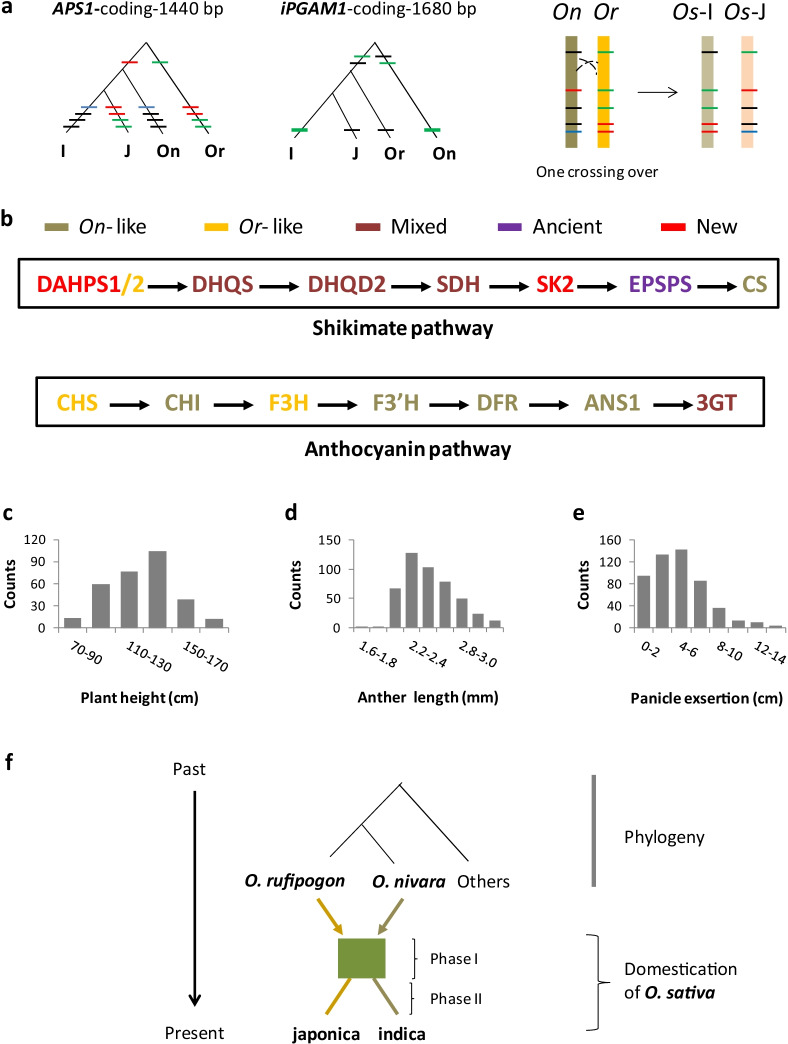
The hybrid origin of *O. sativa*. **a** Gene genealogies showing two recombinant alleles identified in *O. sativa.* One is japonica allele at *OsAPS1*, and the other indica allele at *iPGAM1*. Both can be generated by one crossing-over event during synapsis between chromatids of parental genomes *O. rufipogon* (*Or*) and *O. nivara* (*On*), as shown to the right. **b** Origins of structural genes of two metabolic pathways in rice. **c** The distribution of plant height in *O*. *sativa*. Plant height was measured at harvest on F2 progeny (N = 305) of a cross between Heidao as pollen receiver and Jixuenuo as pollen donor. See Additional file 6: Data 1 for detail. **d** Variation of the anther length just before dispersal of pollen. A total of 65 plants from the F2 population, one *japonica*, and three *indica* cultivars were sampled in two days at noon when flowers (N = 466) were open. See Additional file 7: Data 2 for detail. **e** Degree of panicle exsertion in *O*. *sativa*. A random sample of 232 plants from 46 accessions of indica, japonica, and their crossed progeny were measured at 696 panicles for stem length beyond the sheath of flag leaf. Details see Additional file 7: Data 2. **f** A brief summary of rice domestication. The green rectangle indicates the period when rice was selected as one crop. The colored arrows show contributions of parental genomes, and the bar of the same color indicates slight more contribution of the species to the subgroup. The dots represent other subgroups

Since a hybrid origin is expected to show certain patterns, we examined some expected patterns in Asian rice. The first one is recombinant. Though many historical recombinants have developed mutations to be less recognizable, some accumulated none. We found three *Os* loci in the sample showing recombinants (Fig. 4a). They are *APS1*, which encodes a protein resembling the small unit of glucose-1-phosphate adenylyltransferase in starch synthesis of *Arabidopsis* (Crevillen et al. 2005), *iPGAM1*, a gene presumably encoding phosphoglycerate mutase, and *PK1*, a pyruvate-kinase gene (Additional file 2: Fig. S1k). The second pattern is segregation between the parental genomes. Though the immediate results of segregation are no longer available, the associated patterns remain, as shown at loci of Type 5 (japonica-*Or* and indica-*On* associations) and Type 6 (japonica-*On* and indica-*Or* associations). The two reversal types imply that chromosomal segregation did occur in both directions during the initial synapsis after pairing of the parental genomes, but one type (Type 5) became more common. A reason could be segregation distortion, which was previously reported on hybrid chromosomes of *O. nivara* and *O. rufipogon* created in the laboratory (Grillo et al. 2009). The third pattern is functional complementation, by which we refer to the presence of genes from both ancestors in forming a genetic or biochemical unit. For instance, a metabolic pathway in *O*. *sativa* may contain enzymes encoded by genes from either *O. nivara* or *O. rufipogon*, as shown in the shikimate pathway and the anthocyanin pathway (Fig. 4b).

The hybrid hypothesis also predicts intermediate phenotypes of rice between their parental ancestors. Certain morphological traits vary largely between *O. rufipogon* and *O*. *nivara* (Grillo et al. 2009; Xiong et al. 1999), allowing a test on these traits. Given that japonica genome resembles more *Or* genome while indica genome is closer to *On* genome, we created crossed progeny between Heidao (japonica) and Jixuenuo (indica). Plant height in the F2 generation was more variable than those of their parental plants, with the extremes comparable to heights of their annual and perennial ancestors (Fig. 4c). Similarly, anther length (Fig. 4d) and degree of panicle exsertion (Fig. 4e) measured in the filial generations of the cross and cultivars of rice indicated that they were intermediate between those of *O. rufipogon* and *O*. *nivara* and close to the hybrids of *O. rufipogon* and *O*. *nivara* created artificially (Grillo et al. 2009). All evidence thus far concurs with the hybrid origin of cultivated rice and two-stage domestication (Fig. 4f).

### Co-existence of Ancestral and Derived Alleles in Current Rice Genomes

The hybrid origin and long history of domestication of Asian rice may create locus-specific assortments of alleles. Can *Os* loci that harbored alleles of different origins use them effectively in modern cultivars? To seek the relevant evidence while evaluating precisions of mapped mutations, we sequenced a subset of the sampled loci across diverse cultivars and landraces of rice using Sanger′s method (Additional file 8: Table S5). At *OsAPS1* locus, the recombinant Nipponbare allele (44%) coexist with the indica allele (32%), which is identical to *OnAPS1* in 5′ and coding regions (Table 3). At *OsSSY3* locus (Additional file 9: Table S6), however, the most abundant allele (*OsSSY3_b,* 59%) is a derived one from *O*. *nivara*, with ancestral alleles of *O. rufipogon* (6%) and *O*. *nivara* (1%) rarely observed. For *OsPGMp* (Additional file 10: Table S7), a glycolysis gene, the ancestral *OnPGMp* is the most frequent allele (44%), with *O. rufipogon* contributing little to this locus. This pattern is consistent with its uni-specific (*On*) origin based on gene genealogy (Additional file 2: Fig. S1j). At *OsCKX2* locus, which influences grain production (Ashikari et al. 2005), newly evolved *OsCKX2_a* (Nipponbare allele, 41%) and *OsCKX2_b* (*indica* allele, 40%) have taken the central stage (Additional file 11: Table S8). These data not only confirmed the accuracy of mapped mutations (42 checked and all confirmed) but also indicated that the gene pool of modern rice primarily consists of recombinant, uni-specific, and new alleles.

**Table 3:** Comparisons of model genomes (upper panel) with alleles of *OsAPS1* (lower panel) identified in a survey of rice cultivars

Plant	*APS1*	2698	2778	2865	2951	3020	3072	3131	3293	3298	3321	Frequency
*O. nivara*	*OnAPS1*	T	G	A	T	G	G	A	A	G	G	
*O. sativa* 9311	*OsAPS1*	T	G	A	T	G	G	A	A	G	G	
*O. rufipogon*	*OrAPS1*	A	A	G	G	C	G	G	G	A	G	
*O. sativa* Nipponbare	*OsAPS1*	T	A	G	G	C	G	G	G	A	G	
Other 206 cultivars of *O. sativa*	***OsAPS1_a***	T	G	A	T	G	G	A	A	G	G	0.32
	*OsAPS1_b*	T	A	A	G	G	G	A	A	A	G	0.03
	*OsAPS1_c*	T	A	A	G	G	G	A	A	A	A	0.09
	***OsAPS1_d***	T	A	G	G	C	G	G	G	A	G	0.44
	*OsAPS1_b*′	T	A	A	G	G	A	A	A	A	G	0.12

The nucleotide numbering starts at the first nucleotide (1) of the first exon of *OnAPSL*. The comparison covers nucleotides numbered 2670–3565 between the 2nd exon and the 3rd exon

The most frequent alleles are in bold

In addition to data collected here, we consulted previously reported data at NCBI. From the mutations listed in Table 1, we randomly picked 12 early and 3 later mutations at six loci and compared them against multiple rice varieties reported (Additional file 12: Table S9). The independent data sets largely agree with our identification of the early and later mutations. We then test whether or not the gene types identified here are specific to one subgroup, taking the 5′ region of *Hd3a* (identified as mixed-type above) as an example. Independent data sets from NCBI indicate that the mixed-type is not specific to a subgroup but a feature of the locus (Additional file 13: Table S10).

## Discussion

The GGM analysis above is simple but highly effective for sorting out fixed early polymorphisms, providing a temporal framework for lineage-specific mutations and allowing detection of selection and functional tests of specific mutations. Though early-stage genetic modifications can also be gathered from archeological remains, as shown for maize (Jaenicke-Despres et al. 2003) and grapes (Ramos-Madrigal et al. 2019), these materials are generally scarce. For *O. sativa*, our approach revealed its origin, along with a better understanding of how genetic diversity has accumulated in rice, both of which were thorny issues previously.

### The Hybrid Origin and Subsequent Selection of Asian Rice Explain the Previous Puzzles

Since *O*. *nivara* can easily contribute pollen and *O. rufipogon* can accept outside pollen (Vaughan et al. 2008), a natural hybridization event between *O. rufipogon* as maternal plant and *O*. *nivara* as paternal plant was the most likely scenario for the origin of Asian rice*.* This scenario is compatible with at least three known patterns—its typically watery environment of growth, chloroplast genomes of *O. rufipogon* being basal to those of cultivated rice (Moner et al. 2018), and lack of trait segregation of annual growth when *O*. *sativa* was crossed with *O*. *nivara* (Li et al. 2006a). Once *O*. *nivara* passed down its selfing trait to early rice, further crossing of the progeny as maternal plant with *O*. *nivara* became less likely. Meanwhile, grain-harvesting and subsequent cultivation by human also prevented crossing of early rice with wild *O. rufipogon*. The hybrid origin gave the early rice rich genetic diversity through recombination and segregation. New mutations occurred to inherited alleles and their recombinants were subject to selection by human and environment over the long history of cultivation, which transformed Asian rice, creating its numerous landraces and cultivars along the way.

From the surveyed 101 genes, 12 *On*-like loci and 18 *Or*-like loci were identified (Additional file 1: Table S1). The compositional bias of *Os* genomes toward *O. rufipogon* could be caused by selection favoring *Or* genes in the filial generations, as they supposedly grew in the maternal habitat of *O. rufipogon* after the initial hybridization. This pattern may explain the higher average genetic similarity between *O. rufipogon* and *O*. *sativa* than one between *O. nivara* and *O*. *sativa*. More frequent Type-5 loci (20 sampled) than Type-6 loci (2 sampled) in genomes of Asian rice suggest easily detected japonica-*Or* and indica-*On* associations, as noticed (Cheng et al. 2003). Consequently, part of genetic differentiation between *O. rufipogon* and *O*. *nivara* at Type-5 loci was carried over to subgroups of *O. sativa*, causing the divergence between indica and japonica older than the domestication history.

The hybrid origin of Asian rice can explain both trait segregation and lack of trait segregation in the literature. In the former category, traits such as cold resistance and awn length are bi-specifically determined in rice (as shown by *OsMYB15* and *OsAn-2*, respectively), which permits segregation of a japonica morph in progeny of crosses between an indica rice and *O*. *nivara* (Oka and Morishima 1982). In the latter category, traits such as selfing and annual growth of rice were supposedly inherited from *O*. *nivara*, thus no segregation of the traits would follow in F2 generation of a cross between an indica cultivar and *O*. *nivara* (Li et al. 2006a). Though perennial habit of *O. rufipogon* is dominant to the annual habit of *O*. *nivara* in the first two generations (Grillo et al. 2009), annual growth could appear in further generations and be selected by early breeders. Selected early mutations and their hitchhiking sites necessarily transcended the early rice stock, leaving no signals of introgression at loci of *SH4*, *Rc*, and other 20 *Os* loci (Table 1) during the early domestication.

If the hybrid origin of rice is considered hypothesis A here, it has gained all support so far. An alternative and broadly referenced hypothesis (B) by Huang et al. (2012) finds no support in our GGM analysis (Additional file 14: Fig. S2). Under the hypothesis B, none of *On* alleles or recombinant between *On* and *Or* alleles are expected in japonica genomes, and significantly higher genetic diversity is expected to be seen in genomes of indica than in those of japonica due to the proposed hybrid origin of indica. These predictions, however, are rejected by genomic data (Additional file 14: Fig. S2). Furthermore, the gene genealogies of numerous genomic regions, as shown by those of the coding regions of *C1*, *Hd3a*, and *DFR* (Fig. 3h, i), would have been impossible under the hypothesis B, as the hypothesis denies the presence of *O*. *nivara* mutations in genomes of japonica (Additional file 2: Fig. S1a–l). Since neither indica nor japonica displays strong evidence for having undergone a longer domestication history than the other, the mutation distribution of *O. sativa* supports little of hypothesis-B-like scenarios, i.e. one subspecies was domesticated first and then crossed with a wild species to give rise to the other subspecies.

### Unknown History of Rice Domestication

The single domestication of early rice matches the archeological evidence showing that rice collected from 6600 to 6900 years ago at a south site (Tianluoshan) of the Yangtze River had a gradual increase of non-shattering grains from 27 to 39% (Fuller et al. 2009). If the domestication started about eight to nine thousand years ago (Gross and Zhao 2014), this documented archeological period fell in Phase I domestication when *OsSH4* was still under active selection. Rice in this period had undergone much domestication but still differed from later rice. As mutations were gradually accumulated over time, the current *sh4* allele is evidently not one that contributed to non-shattering grains thousands of years ago.

Detected positive selection and functional validations of some of the early mutations suggest that the early human selection on Asian rice had concentrated on traits of not only grain shattering, color, or awn length, but also flowering, yield, and likely rice taste and tolerance. This extensive range of trait modifications by human selection had likely made the early rice an attractive crop by the end of Phase I. Then, the split domestication came about 4–5 millennia ago, which could be either based on phenotypic differences (including taste), if distorted segregation could lead to some progeny having more genes from *O. nivara* (proto-indica) and others having more genes from *O. rufipogon* (proto-japonica), or phenotype-irrelevant, since different environments and/or selection criteria could also lead to the differentiation of subgroups. Under either scenario, separate cultivations of Asian rice were inevitably an event of historical importance, as it involved divided labor and possibly people(s), though the cause is unclear.

Following the historical event, selections over the second stage of domestication were largely isolated from each other, judging from fixation of lineage-specific mutations in genomes of nucleus and organelles, causing phylogenetic divergence between indica and japonica within Asian rice (Tong et al. 2016; Choi et al. 2017; Cheng et al. 2019). This period lasted until the modern age, and recent hybridizations between indica and japonica by human, though increasing quickly, are still relatively rare among rice varieties and can hardly alter the historical patterns presented here.

### Genomic Basis of Selected Phenotypes

Since a significant portion of positive selection was operated on 5′ mutations, phenotypic alterations during rice domestication were more likely through adjusting gene expression than by modifying protein. For instance, transcript enhancements of *Hd3a* and *OsDFR* were positively selected during the early domestication (Table1; Fig. 2). This domestication history of *Hd3a,* along with that of *Hd1,* expands a recent report of their positive selections in japonica cultivars (Liu et al. 2021a). In another example, positively selected four 5′ mutations of *Chalk5* happened only in Phase II in indica, two (thymines) of which were previously demonstrated to abate expression levels of *Chalk5* (Li et al. 2014). We show that, as a Type-5 gene, *Chalk5* inherited different alleles from *O. nivara* and *O. rufipogon* and passed them down to indica and japonica lineages, respectively (Additional file 2: Fig. S1e). Whether or not the ancestral differences also contributed to texture dissimilarity between indica and japonica requires additional investigations.

Certain patterns have been made clear for known *Os* genes—loci influencing productivity and under positive selection increased from four in Phase I to ten in Phase II. Meanwhile, positive selection was expanded from three to five loci for flowering time. These patterns reflect increased breeding effort for high yield and broader regions of rice planting in Phase II, a trend continued until this date. Consistently, domestication has promoted emergence of new alleles at *Os* loci affected.

### Promises and Caveats

The major advantage of obtaining a mutation-based gene genealogy is its robustness to species boundaries, selection, recombination, and not much influenced by random events such as bottleneck or incomplete lineage sorting, which make the approach particularly fit for understanding domestication of crops. Depending on availability of genomes from various lineages, mutations can be allocated on inner branches of a gene-genealogy, providing information on largely fixed mutations at earlier stages for an evolutionarily recent process of differentiation (e.g. domestication).

Cautions need to be exercised, however, in applying the method. One is the lack of resolution for detecting the temporal order of single mutations within a branch when multiple mutations occurred within the period. The second is that detection of positive selection based on mutation rate of introns can be highly conservative when introns themselves are influenced by selective processes, leaving human selection at a moderate or low intensity less likely to be detected using the criteria given here. One can either use a less stringent threshold or switch to other appropriate base-line for comparison. Due to different criteria used for detecting positive selection across regions, an inference about inter-region comparison of positive selection should be taken as a hypothesis and subject to more evidence. The third caution is on the evaluation of new mutations in the focal group. Only substitutions or/and indels present in crop (offpring) genomes and absent in relatives′ (ancestral) genomes are classified as new mutations, the genomes of wild relatives thus serve as references for each monomorphic site in crops, leaving polymorphic sites between wild relatives only used for inferring the topology of a gene genealogy. For evaluating fixed polymorphisms (McDonald and Kreitman 1991) early in domestication, the minimum sample size for our method to be informative consists of two high-quality genomes representing diverse groups of a crop and two high-quality genomes from its wild relatives.

If the goal of an investigation is to know historical events/patterns, a small to moderate number of high-quality and independent genomes are often sufficient. If a sample of all mutations follows the binomial distribution and everything else is equal, two independent genomes, when being the same at a given nucleotide site, may give 75% assurance for identifying a monomorphic site and four such genomes raise the probability to 94%. The probability can be much higher if the substitution models are taken into account. Increasing genome number may, of course, lead to more mutations identified, but transient mutations (ones of low frequencies) tend to rise disproportionally, too, which may inflate end-branch length when the base line of comparison is the historical and fixed mutation. To illustrate this point, we compared the genome of Kitaake (PRJNA448171), another well-sequenced *japonica*, against our earlier classification of mutations of the 101 genes. The Kitaake genome differs from the Nipponbare genome at 43 of the 202 regions but agrees with all of the early fixed mutations except one indel (1/91) and most (652/665) of the later mutations (Additional file 15: Table S11). The disagreements (< 2%) have a negligible effect on our analysis. Among the 43 regions, 3 in 2 genes are identical to those of 9311, suggesting some level of genetic introgression; one region (the coding of *RAE2*) is a recombinant between indica and japonica, and the rest appears largely transient.

The biggest caveat, nonetheless, comes from sequencing errors or missing sites, which can be corrected by re-sequencing critical sites if necessary. Clearly, genomes of uncertain alignments are not fit for the method. This makes the GGM method particularly fit for studies on a process of recent differentiation.

## Conclusions

We show that domestication history of Asian rice can be divided into two phases of similar lengths. The rice in the first or early phase was a single crop and the rice in the later or divergent phase was represented by two subspecies, referred to as indica and japonica. Early mutations of domestication can now be delineated by stratifying mutations on an inner branch of gene genealogy, enabling researchers to evaluate impacts of specific mutations on phenotypic traits over a historical period. This has led to better understanding of how selection has transformed Asian rice and broadening of the genetic basis for future breeding. One message brought by the history of domestication is that positive selection at 5′ regions may occur more frequently than one at coding regions. This selective strategy is mild to plant and likely healthy to the *Os* genomes as well. Knowing the origin of allelic diversity and history of human selection helps reconstruct past achievements of agriculture, while assisting better varieties to be created in future.

## Methods

### Gene Sampling and Alignment of Orthologs

Sampled genes (Additional file 1: Table S1) should cover all chromosomes in order to capture genome-wide patterns, though the proportions of unknown and known genes can be arbitrary. Known genes are essential, as they allow inferences on mutations and traits of human selection. Including unknown genes is to make sure that no great bias is introduced by sampling known genes. Information of selection can be gathered regardless of the status of gene.

For the purpose of identifying fixed mutations within a domesticated species, diverse genomes should be included, along with genomes of wild relative(s). The specific period of domestication is defined by selected genomes, thus sampling of genomes are not totally random. For instance, if the early period before subgroups is targeted, samples from subgroups are needed. A minimum of two diverse genetic groups per domesticate should be included in order to provide a topology needed for inferring early mutations. For rice, high-quality nuclear genomes were chosen at the NCBI web site (https://www.ncbi.nlm.nih.gov/, accessed 2019.4.21–2021.2.18), including *O. rufipogon* (PRJEB4137, strain W1943), *O. nivara* (PRJNA48107, strain IRGC 100897), one cultivar (PRJDB1747, Nipponbare) of *japonica* and two cultivars, Shuhui (PRJNA302543, Shuhui-498) and 9311(PRJNA427873), of *indica*. A third *indica* cultivar Bio226 (PRJNA285384, RP Bio-226) was consulted only in the case of insufficient information in the previous two genomes, as its coverage (~ 20 ×) was relatively low. For Asian rice, a total of 101 loci were sampled to represent 12 chromosomes, genetic pathways of interest, and ones related to agricultural traits. Following BLAST searches of the model genomes using the reference genes as baits, we obtained homologous sequences and identified the best matches as candidate orthologs. The homology of the candidate sequences was further examined for chromosomal location, gene structure, and similarity to the reported reference to ensure orthologs of one another. Two genealogies, one based on the 5′ regions (5′ genealogy) and the other on the entire coding regions (coding genealogy), were then built. This dual representation of a gene is based on their different functions, the former influencing protein quantity and the latter affecting protein function (quality).

Fragments of genomic sequences covering the whole gene from 1 Kb 5′ upstream to about 200 bp of 3′ downstream were sliced in Sequencher (version 4.10.1, Gene Codes) or directly from the genome using online tools at the NCBI website. The 1 Kb 5′ regions are generally well aligned, but can be shorter due to difficulty in alignments in some cases. We aligned sequences in MegAlign (version 8+, DNASTAR) using ClustalW and adjusted the alignment manually when necessary. For many genes of small variation, manual alignments are sufficient.

### Construction of Gene Genealogy and Identification of Early Mutations

A gene genealogy here can be viewed as a graphic translation of an unambiguous alignment of DNA sequences for the purposes of inferring mutation distribution. The oldest mutations on the gene genealogy are those polymorphic between *O. rufipogon* and *O. nivara*, which are mainly for setting the topology of a gene genealogy. The targeted mutations are those within lineages of *O. sativa*. We mapped these mutations in two interchangeable forms of genealogy. The traditional tree-like gene genealogy shows better the temporal distribution of mutations and suits for small numbers of mutations such as those of coding sequences. A gene genealogy in bar graph displays better the spatial distributions of mutations and fits for a large number of mutations as typically seen at 5′ region. All genealogies had at least four terminal units connecting orthologs of *O. rufipogon*, *O*. *nivara*, *O*. *sativa* japonica, and *O*. *sativa* indica. As *O. nivara* was considered derived from *O. rufipogon*, we labeled nonsynonymous changes in the lineage of *O. nivara* when the situation was permitted. This labeling is tentative and has no direct impact on the subsequent analysis here. The second category of mutations includes ones shared between *japonica* and *indica* but absent in both *O. rufipogon* and *O. nivara*, which were the early mutations. Mutations emerged after the split of japonica and indica within *O. sativa* belong to the third category, which may include a small quantity of transient ones by chance. Recurrent mutations are unlikely due to the shallow history of domestication, thus not considered.

### Detection of Positive Selection

For the 5′ gene genealogy, an intron-based test of positive selection was developed. Relative to 5′ regions, introns seldom influence gene function and are less likely subject to selection. To estimate the background mutation rates (*u*, in the unit of mutations per 1 Kb nucleotides over the period (lineage) considered) of the *Oryza* genomes, we collected mutations observed in a random sample of introns located within the sampled genes and longer than 800 bp. The purpose of sampling long introns was to make them more comparable to the lengths of 5′ region and average coding regions. Naturally occurring mutations in *Os* introns were averaged as a proxy for the background mutations (the higher of the two estimates between indica and japonica lineages was taken for Phase II). Since positive selection can elevate mutation rate, the cut-off value for the signal of positive selection in 5′ regions was set at a stringent level of more than three times the background-level mutation rate (3*u*), which includes both substitutions and indels.

For the coding gene genealogy, codon-based detection of positive selection (Nielsen and Yang 1998; Yang et al. 2000) can be readily applied to cases when substitutions are sufficient to permit the test. The average rate of synonymous substitutions per site (*d*_*S*_) and that of nonsynonymous substitutions per site (*d*_*N*_) can be computed in DNAsp (Rozas et al. 2017) or PAML (Yang 2007). We applied the stringent condition of *d*_*N*_/*d*_*S*_ > 1 for positive selection (Nielsen and Yang 1998) on the japonica and indica lineages. Because mutation numbers on the early lineage are often low and *d*_*S*_ can be zero, we applied a simple test using a cut-off value of two amino-acid changes per 1 Kb sequence as the signal of positive selection in these cases. The test is more stringent than that of *d*_*N*_/*d*_*S*_, as it is equivalent to regarding all sites as nonsynonymous.

### Estimation of the Relative Duration of a Recent Lineage

Because one plant of the *Oryza* groups can generate a reasonable gamete pool, genetic drift is expected to be small. Only mutations and selection under the infinite site model are considered for Asian rice. Let the background mutation rate be *v* per generation, effective size of rice population be *N*_*e*_, neutral mutations are expected to occur in a quantity of 2*vN*_e_ per generation. If mutations were under selection at intensity *s*, their fixation probability (P) would be (1−e^−4*sqNe*^)/(1−e^−4*sNe*^) according to Kimura (1962), where *q* is the initial frequency of the mutation. Let *q* = 1/(2 N), here N denotes the census population size, P = (1−e^−(2*sNe)/N*^)/(1−e^−4*sNe*^), which can be approximated by 2* s* when *s* is small and *N*_e_ equals N (Li 1997). However, since *N*_*e*_ in a selfing population is reduced to 1/2 of N (Nordborg and Donnelly 1997), P ≈ 1−e^−*s*^ in Asian rice. It suggests that P is largely *s*-dependent. The expected number (m) of fixed mutations per lineage can be approximated by 2*vN*_*e*_PT, where T denotes the total generations spent in a lineage. For the early lineage where mutations were expected to accumulate in the quantity of m_e_ in T_e_ generations, T_e_ can be computed by m_e_/(2*vN*_*e*_P). Similarly, for the lineage of *indica*, where m_i_ mutations are expected to be fixed, T_i_ (time in *indica* lineage) can be computed by m_i_/(2*vN*_*e*_P). The whole history of rice (T_w_) can thus be estimated up by (T_e_ + T_i_) or (T_e_ + T_j_), where T_j_ refers to the generation time in *japonica* lineage. The relative duration of T_e_ becomes T_e_/T_w_, which can be measured simply by m_e_/(m_i_ + m_e_) or m_e_/(m_j_ + m_e_), where m_j_ is the number of expected mutations fixed in *japonica* lineage. By letting the expected mutation numbers equal to the observed ones (assuming transient mutations are rare in the samples and negligible), the relative duration of the early lineage (T_e_/T_w_) can be estimated for rice. We used only loci that accumulated mutations throughout domestication for the inference to reduce bias of estimation.

### Nucleotide Extractions and cDNA Amplifications

Total RNAs were obtained from fresh grains collected in a Paddy field using TRNzol total RNA Reagent (Tiangen, Beijing, China) following the method of Wang et al. (2012). About 2 µg RNAs was then used for the cDNA synthesis via TIANScript II RT Kit (Tiangen, Beijing, China). For each PCR reaction, about 100–500 ng cDNA was taken as the template to amplify targeted genes with specific primers. Total genomic DNA was gathered from young leaves freshly collected from the field by the CTAB method.

### Constructs Used in Functional Experiments

To examine whether OsMYB3 was able to activate *OsDFR *in vivo, we constructed CaMV 35S-driven-transcription factor (TF) vector pOsMYB3 by replacing the coding sequence of pJIT163 (Guerineau et al. 1992) with that of *OsMYB3* from cDNAs, which was prepared from RNAs extracted from developing grains of Heidao. On the same backbone, we built reporter vector pOsDFR_pro1K_ from 1041-bp long 5′ region of *OsDFR*, which was amplified from leaf gDNA of Heidao using primers OsDFRpro0921-F and OsDFRpro0921-R (Additional file 16: Table S12). The second reporter vector pOsCHS_pro1K_ was based on 1 Kb-long 5′ sequence of *OsCHS1* from Heidao′s gDNA using amplification primers (OsCHS_pro_-EcoRIF and OsCHS_pro_-MluIR).

Further TF-vectors were built from the same backbone above using the coding sequences of *OrMYB3* or *OnMYB3*. OrMYB3 were adapted from OsMYB3 by introducing needed changes with linkers (Orlink1r1, Orlink1r2, and Orlink2f) and primers (OsTT2-163-SalIF and OsTT2-163-EcoRI_R). OnMYB3 was similarly obtained with linkers (OnTT2cag-f, OnTT2cag-r, OnTT2tlnk-r, OnTT2tlnk-f, Onlink1r, and Onlink2) and primers (OnTT2-163TF-F and OnTT2-163TF-R).

Mutation-specific reporters were constructed for pOsDFR_pro-370_ from 370 bp 3′ end of pOsDFR_pro1K_ using primers (OsDFRp370f and OsDFRpro0921-R) and for pOrDFR_pro-369_, which hosted that same 5′ sequences except at the two mutated sites, using primers (OrDFRp369f and OsDFRpro0921-R).

For 5′ UTR mutation in *Hd3a*, a single substitution (T in *Or* → A in *Os*) at the TATA site of *OsHd3a* was designed into two reporter vectors based on pOsDFR_pro1K_ by replacing the local 5′UTR of *OsDFR* with that of *OsHd3a* or *OrHd3a*. To build the nested vector pOsHd3a, we took pOsDFR_pro1K_ as the template and two linkers (OsHd3alinker1 and Hd3alinker2) and primers OsDFRpro0921-F and OsDFRpro0921-R as interacting components in PCR reactions. The desired 5′ sequence was inserted back to pJIT163 at enzyme cut sites. Similarly, pOrHd3a was created from two linkers (orHd3alinker1and Hd3alinker2) and the same primers. With pJIT163, we further synthesized three vectors from the coding sequences of *OsC1*, *OsB2*, and *OsTTG1*. OsC1 and OsTTG1 were cloned from the cDNAs of Heidao and OsB2 from that of Jixuenuo. All primers were synthesized by Sangon (Shanghai, China). Constructs were all sequenced to ensure correct alignments and sequences.

### Transient Expressions

We used a yellow-husked *indica* material (B16-44) to prepare the protoplasts for the following tests of transient gene expression using fluorescent firefly luciferase (LUC) as the reporter gene. Protoplast isolation and transformation followed the previous protocol of Yoo et al. (Yoo et al. 2007) but the incubation temperature used 28 °C. The two constructs, pOsMYB3 and pOsDFR_pro1K_ (2 μg of each), were co-introduced into rice protoplasts, along with 0.2 μg renilla-luciferase (RUC)-expressing vector pRUC as internal control. After incubating at 28 °C for 16 h, the culture was analyzed via the dual luciferase reporter assay system (Promega, Madison, WI. USA), following the manufactory′s protocol. The experiment was repeated with pOsMYB3 and pOsCHS_pro1K_ using the same routine.

After knowing the activation capacity of OsMYB3, we compared it with OnMYB3 and OrMYB3 in their activation capacities on the same promoter of pOsDFR_pro1K_. For two promoter mutations within 370 bp 5′ region of *OsDFR*, we compared their activation levels measured after co-infections of pOsDFR_pro-370_ (or pOrDFR_pro-369_) and TF-vector pOsMYB3 (2 μg of each), along with the internal control as above.

Effect of the 5′UTR mutation in *Hd3a* on gene expression was compared between two reporters, pOsHd3a and pOrHd3a, by subjecting them to the regulatory complex consisted of products from three regulatory vectors. The experimental procedure followed the same protocol mentioned above.

### Morphological Traits

Besides nuclear data, morphological traits were collected in two field experiments and compared to reported values on *O. rufipogon* (Xiong et al. 1999), *O*. *nivara* (Grillo et al. 2009; Li et al. 2006a), and their hybrids (Grillo et al. 2009). In the first experiment, we measured plant height in F2 generation of a cross between Heidao of *japonica* and Jixuenuo of *indica*. Mature plants were harvested just above ground and hang upside down to allow measuring of height using a meter ruler (1 mm). In the second experiment, length of an anther was measured on unopened pollen sacs of freshly opened flowers by a caliper (0.01 mm) on 26 accessions of the filial generation of the cross. Toward grain maturation, 48 accessions were sampled for degree of panicle exsertion, measured as the distance from the basal node of a panicle to the top of leaf sheath surrounding the panicle by a ruler (1 mm).

### Sequencing Surveys

Current allelic diversity was surveyed in polymerase chain reactions using leaf gDNAs and gene-specific primers (Additional file 16: Table S12). The gDNAs were extracted from seedlings of rice cultivars with the conventional CTAB method. The 5′ region of *C1* gene was similarly amplified from leaf gDNA by specific primers (Additional file 16: Table S12). All sequences were edited against valid chromatograms when necessary.

## Supplementary Information

**Additional file 1: Table S1.** Information on 101 genes analyzed in this study.

**Additional file 2: Fig. S1.** Mapping of mutational events. **a** Chromosome 1. **b** Chromosome 2. **c** Chromosome 3. **d** Chromosome 4. **e** Chromosome 5. **f** Chromosome 6. **g** Chromosome 7. **h** Chromosome 8. **i** Chromosome 9. **j** Chromosome 10. **k** Chromosome 11. **l** Chromosome 12.

**Additional file 3: Table S2.** A list of 30 loci showing early and later mutations in rice.

**Additional file 4: Table S3.** Estimation of background-mutation rates from intron mutations of four genomes (*Or, On, Indica* (I) and Nipponbare (J)).

**Additional file 5: Table S4.** Distribution of eight gene types (shown in Fig. 3) among 101 sampled loci of *O. sativa*.

**Additional file 6: Data 1.** Plant height of F2 population in the field experiment 1.

**Additional file 7: Data 2.** Morphological data in the field experiment 2.

**Additional file 8: Table S5.** Genotypes at four Os loci across accessions.

**Additional file 9: Table S6.** Comparisons of the model genomes (upper panel) with alleles of *OsSSY3* (lower panel) in rice cultivars.

**Additional file 10: Table S7.** Comparisons of model genomes (upper panel) with alleles of *OsPGMp* (lower panel) surveyed in rice materials.

**Additional file 11: Table S8.** Comparisons of model genomes (upper panel) with alleles of *OsCKX2* (lower panel) surveyed in rice.

**Additional file 12: Table S9.** Random samples on identified polymorphisms from NCBI.

**Additional file 13: Table S10.** Population-level examinations of mixed contributions of *Or* and *On* to *OsHd3a* based on NCBI data.

**Additional file 14: Fig. S2.** Comparisons of predictions under different hypotheses.

**Additional file 15: Table S11.** Genomic comparisons of Kitaake with reference genomes across 101 genes.

**Additional file 16: Table S12.** List of primers used in this study.

## Data Availability

All data generated or analyzed during this study are included in this publication [and its Additional files]. The experimental materials are available from the corresponding author on reasonable request.

## Abbreviations

B16-44: An accession from interbreeding of two white-rice cultivars
*d*_*S*_: The average rate of synonymous substitutions per site
*d*_*N*_: The average rate of nonsynonymous substitutions per site
GGM: Gene genealogy-based mutation analysis
Kb: Kilobase
m: The expected number of fixed mutations in a lineage
LUC: Luciferase of firefly
N: Population size by census
*N*_*e*_: Effective population size
NCBI: National Center for Biotechnology Information, National Institute of Health, USA
*Or*: *Oryza rufipogon*
*On*: *Oryza nivara*
*Os*: *Oryza sativa*
P: Fixation probability
RUC: Renilla luciferase
*s*: Selection intensity
T: Total generations spent in a lineage
TF: Transcription factor
5′ UTR: Untranslated segment at the 5′ region of a gene
*u*: Background mutation rate over a lineage (or period)
*v*: Background mutation rate per generation
