# Supplementary material for: Early Domestication History of Asian Rice Revealed by Mutations and Genome-Wide Analysis of Gene Genealogies
Source: Rice (N Y). 2022 Feb 15;15:11. doi: 10.1186/s12284-022-00556-6 (PMC8847465; doi:10.1186/s12284-022-00556-6)
Supplement: Supplementary file 1 — Additional file 1: Table S1. Information on 101 genes analyzed in this study. [file 12284_2022_556_MOESM1_ESM.pdf]

## **Additional file 1**

**Supplemental Table 1**. Information on 101 genes analyzed in this study.

| Chromosome | Rice gene | RAP ID | MSU ID[a] | Comparison of rice gene to homologs of the wild relatives[c] | | Known function[d] | Number of mutations in *O. sativa*[f] | |
|---|---|---|---|---|---|---|---|---|
| | | | | 5' region (1 Kb) | Coding region(s) | | 5' region (1 Kb) | Coding regions |
| **1** | ***ME*** | Os01g0188400 | Os01g09320.1 | Mixed | Mixed | Malic enzyme | 3 | 5 |
| | ***CKX2*** | Os01g0197700 | Os01g10110.1 | New I | New | Cytokinin oxidase | 7 | 5 |
| | ***ANS1*** | Os01g0372500 | Os01g27490.1 | On-like[b] | On-like | Flavonoid network | 1 | 5[e] |
| | ***DFR*** | Os01g0633500 | Os01g44260.1 | Mixed | On-like | Flavonoid network | 9 | 4 |
| | ***NOG1*** | Os01g0752200 | Os01g54860.1 | New J | Mixed | Grain number | 24 | 2 |
| | ***CM3*** | Os01g0764400 | Os01g55870.1 | New I[b] | Mixed | *Shikimate pathway* | 6 | 2 |
| | ***iPGAM1*** | Os01g0817700 | Os01g60190.1 | Mixed[b] | Mixed | *Glycolysis* | 4 | 0 |
| | ***qSH1*** | Os01g0848400 | Os01g62920.1 | *O. nivara* | Mixed | Seed shattering | 0 | 1 |
| | ***SD1*** | Os01g0883800 | Os01g66100.1 | New J | Nearly Identical amino acids (aa) | Gibberellin biosynthesis | 6 | 4 |
| **2** | ***SBE3*** | Os02g0528200 | Os02g32660.1 | Or-J &On-I | Or-J &On-I | Starch branching enzyme | 0 | 0 |
| | ***ZB8*** | Os02g0627100 | Os02g41680.1 | Mixed | Or-like | Phenylalanine ammonia-lyase | 4 | 1 |
| | ***TTG1*** | Os02g0682500 | Os02g45810.2 | Or-J &On-I | Or-J &On-I | WDR gene | 0 | 1 |
| | ***SK2*** | Os02g0687500 | Os02g46220.1 | New | Nearly identical aa | *Shikimate pathway* | 16 | 2 |
| | ***FLS*** | Os02g0767300 | Os02g52840.1 | Or-J &On-I | Identical aa | *Flavonoid network* | 2 | 2 |
| | ***DTH2*** | Os02g0724000 | Os02g49230.1 | Or-J &On-I | Or-J &On-I | Flowering | 1 | 1 |
| **3** | ***MADS1*** | Os03g0215400 | Os03g11614.1 | Mixed | Identical nucleotides (nt) | Grain length | 7 | 0 |
| | ***CS*** | not listed | Os03g14990.1 | Mixed | On-like | *Shikimate pathway* | 4 | 1 |
| | ***LAR*** | Os03g0259400 | Os03g15360.2 | Mixed | Mixed | *Flavonoid network* | 2 | 1 |
| | ***Unknown*** | Os03g0330200 | Os03g21260.1 | Mixed | Mixed | Unknown | 6 | 1 |
| | ***SUS2*** | Os03g0340500 | Os03g22120.2 | On-like[b] | Identical aa | *Sugar metabolism* | 3 | 1 |
| | ***DAHPS1*** | Os03g0389700 | Os03g27230.1 | New I | New I | *Shikimate pathway* | 9 | 5 |
| | ***GS3*** | Os03g0407400 | not listed | *O. rufipogon* | *O. rufipogon* | Grain length | 1 | 1 |
| | ***MYB3*** | Os03g0410000 | Os03g29614.1 | Or-like | Or-like | *Flavonoid network* | 0 | 2 |
| | ***GL3.2*** | Os03g0417700 | Os03g30420.1 | New | On-like | Grain development | 22 | 3 |
| | ***TB1*** | Os03g0706500 | Os03g49880.1 | Mixed[b] | Or-like | Branching | 2 | 2 |
| | ***Hd6*** | Os03g0762000 | Os03g55389.1 | Mixed[b] | Nearly identical aa | Flowering | 2 | 1 |
| | ***Dst*** | Os03g0786400 | Os03g57240.1 | Mixed | New J | Regulator of *CKX2* | 7 | 5 |
| | ***CHI*** | Os03g0819600 | Os03g60509.1 | On-like[b] | On-like | Flavonoid network | 2 | 1 |
| | ***ASA1*** | Os03g0826500 | Os03g61120.1 | Mixed | Mixed | *Amino-acid synthesis* | 8 | 1 |
| **4** | ***An-1*** | Os04g0350700 | Os04g28280.2 | Or-like | New | Awn development | 5 | 11 |
| | ***GIF1*** | Os04g0413500 | Os04g33740.1 | Mixed | Mixed | Grain filling | 10 | 6 |
| | ***MYB15*** | Os04g0517100 | Os04g43680.1 | Or-like | Or-like | *Stress tolerance* | 5 | 3 |
| | ***An-2*** | Os04g0518800 | Os04g43840.1 | Mixed | Mixed | Awn length | 4 | 1 |
| | ***Unknown*** | Os04g0557200 | Os04g47040.1 | Mixed | New J | Unknown | 2 | 3 |
| | ***B2*** | Os04g0557500 | Os04g47059.1 | On-J & Or-I | New J | bHLH gene | 2 | 7 |
| | ***B1*** | Os04g0557800 | Os04g47080.1 | Mixed | New | bHLH gene | 1 | 7 |
| | ***AGO2*** | Os04g0615700 | Os04g52540.1 | New J | New | Grain length/salt tolerance | 13 | 23 |
| | ***IPK1*** | Os04g0661200 | Os04g56580.1 | Mixed | Identical aa | Mineral transport | 2 | 1 |
| | ***F3H*** | Os04g0662600 | Os04g56700.1 | Or-J &On-I | New J | Flavonoid network | 6 | 5 |
| | ***SH4*** | Os04g0670900 | Os04g57530.1 | Mixed | Or-like | Seed shattering | 7 | 2 |
| | ***PK3*** | Os04g0677500 | Os04g58110.1 | Or-J &On-I | Identical aa | *Glycolysis* | 6 | 4 |
| | ***SHAT1*** | Os04g0649100 | Os04g55560.1 | Or-J &On-I | Nearly identical aa | Seed shattering | 2 | 3 |
| **5** | ***Chalk5*** | Os05g0156900 | Os05g06480.1 | Or-J &On-I | Or-J &On-I | Endosperm chalkiness | 4 | 3 |
| | ***GS5*** | Os05g0158500 | Os05g06660.1 | Mixed | New | Grain width | 9 | 8 |
| | ***qSW5*** | Os05g0187500 | Os05g09520.1 | Or-like | *O. rufipogon* | Grain size | 1 | 0 |
| | ***unknown*** | Os05g0196600 | Os05g10780.1 | On-J & Or-I | Mixed | Unknown | 7 | 5 |
| | ***ACC7*** | Os05g0319200 | Os05g25490.1 | Or-J &On-I | Or-J &On-I | *Ethylene synthesis* | 1 | 0 |
| | ***C4H*** | Os05g0320700 | Os05g25640.1 | Or-J &On-I | Mixed | *Cinnamate 4-monooxygenase* | 5 | 1 |
| | ***SH5*** | Os05g0455200 | Os05g38120.1 | Mixed | Mixed | Seed shattering | 4 | 4 |
| | ***T6PS*** | Os05g0518600 | Os05g44210.1 | Mixed | Mixed | *Trehalose-6-phosphate synthase* | 8 | 6 |
| **6** | ***EPSPS*** | Os06g0133900 | Os06g04280.1 | New | Identical nt | Shikimate pathway | 4 | 0 |
| | ***Hd3a*** | Os06g0157700 | Os06g06320.1 | Mixed | New J | Flowering pathway | 4 | 3 |
| | ***SSY1*** | Os06g0160700 | Os06g06560.1 | *O. rufipogon*[b] | *O. rufipogon* | *Starch metabolism* | 0 | 0 |
| | ***C1*** | Os06g0205000 | Os06g10340.1 | Or-like | On-like | Flavonoid network | 2 | 1 |
| | ***TCP19*** | Os06g0226700 | Os06g12230.1 | Or-like | *O. rufipogon* | Tillering response to nitrogen | 4 | 0 |
| | ***Hd1*** | Os06g0275000 | Os06g16370.1 | Mixed | New I | Flowering pathway | 4 | 6 |
| | ***3GT*** | Os06g0291100 | Os06g18790.1 | Mixed | Or-J &On-I | *Flavonoid network* | 10 | 0 |
| | ***vATPB1*** | Os06g0568200 | Os06g37180.1 | Mixed | Mixed | *Photosynthesis* | 4 | 2 |
| | ***GL6*** | Os06g0666100 | Os06g45540.1 | New I | Mixed | Grain length | 5 | 1 |
| **7** | ***PROG1*** | Os07g0153600 | Os07g05900.1 | Or-like | Or-like | Growth angle | 3 | 1 |
| | ***bZIP58*** | Os07g0182000 | Os07g08420.1 | Or-J &On-I | New | Starch metabolism | 0 | 7 |
| | ***Rc*** | Os07g0211500 | Os07g11030.1 | Mixed | Mixed | Flavonoid network | 5 | 4 |
| | ***SSH1*** | Os07g0235800 | Os07g13170.1 | New J | New J | Seed shattering | 9 | 4 |
| | ***Ghd7*** | Os07g0261200 | Os07g15770.1 | Or-J &On-I | Or-J &On-I | Grain productivity | 4 | 1 |
| | ***SDR4*** | Os07g0585700 | Os07g39700.1 | Mixed | Mixed | Seed dormancy | 3 | 10 |
| | ***BG2*** | Os07g0603700 | Os07g41240.1 | Mixed | Or-J &On-I | Grain growth | 10 | 1 |
| | ***DAHPSp*** | Os07g0622200 | Os07g42960.1 | Mixed | Mixed | *Shikimate pathway* | 1 | 1 |
| | ***NADH*** | Os07g0645400 | Os07g45090.1 | Mixed | Mixed | *Energy transportation* | 5 | 0 |
| | ***PRR37*** | Os07g0695100 | Os07g49460.1 | Mixed | Mixed | Circadian clock | 3 | 3 |
| **8** | ***Hd5(Gdh8)*** | Os08g0174500 | Os08g07740.1 | Or-J &On-I | Or-J &On-I | Grain productivity | 6 | 2 |
| | ***SSY3*** | Os08g0191500 | Os08g07740.1 | On-like | On-like | Starch metabolism | 2 | 7 |
| | ***APS1*** | Os08g0345800 | Os08g09230.2 | Or-J &On-I | Or-J &On-I | *Starch metabolism* | 0 | 0 |
| | ***CM4*** | Os08g0441600 | Os08g34290.1 | Mixed | Mixed | *Shikimate pathway* | 2 | 1 |
| | ***RAE2*** | Os08g0485500 | Os08g37890.1 | New | New J | Awnless | 3 | 3 |
| | ***IPA1*** | Os08g0509600 | Os08g39890.1 | Or-like | Or-like | Plant architecture | 1 | 0 |
| | ***SPL16*** | Os08g0531600 | Os08g41940.1 | Mixed | Mixed | Grain width | 6 | 3 |
| **9** | ***unknown*** | Os09g0440600 | Os09g26890.1 | Mixed | Mixed | Unknown | 8 | 5 |
| | ***unknown*** | Os09g0440700 | Os09g26900.1 | Mixed | Identical nt | Unknown | 8 | 0 |
| | ***DEP1*** | Os09g0441900 | Os09g26999.1 | Mixed | Mixed | Panicle morphology | 4 | 2 |
| | ***PGI*** | Os09g0465600 | Os09g29070.1 | Mixed | Mixed | Glycolysis | 4 | 2 |
| | ***bZIP73*** | Os09g0474000 | Os09g29820.1 | Or-J &On-I | Or-J &On-I | Cold tolerance | 1 | 0 |
| | ***PRR95*** | Os09g0532400 | Os09g36220.1 | Mixed[b] | Mixed | *Circadian clock* | 1 | 1 |
| | ***DHQS*** | Os09g0539100 | Os09g36800.1 | Mixed | Mixed | *Shikimate pathway* | 6 | 4 |
| **10** | ***PGMp*** | Os10g0189100 | Os10g11140.2 | On-like[b] | *O. nivara* aa | *Sugar metabolism* | 7 | 3 |
| | ***F3'H*** | Os10g0320100 | Os10g17260.1 | Mixed[b] | *O. nivara* aa | Flavonoid network | 2 | 2 |
| | ***Ehd1*** | Os10g0463400 | Os10g32600.1 | Mixed, new I | Or-like | Flowering time | 7 | 1 |
| | ***DAHPS2*** | Os10g0564400 | Os10g41480.1 | Or-like | Or-like | *Shikimate pathway* | 5 | 1 |
| | ***MYC2*** | Os10g0575000 | Os10g42430.1 | Mixed | Mixed | *Jasmonate signaling* | 2 | 1 |
| | ***NRT1.1B*** | Os10g0554200 | Os10g40600.1 | Or-like | Mixed | Nitrogen usage | 0 | 1 |
| **11** | ***PK1*** | Os11g0148500 | Os11g05110.2 | Mixed | Identical aa | *Glycolysis* | 11 | 0 |
| | ***unknown*** | Os11g0181100 | Os11g07910.1 | Mixed | *O. nivara* aa | Unknown | 8 | 0 |
| | ***ADH2*** | Os11g0210500 | Os11g10510.1 | Mixed[b], new J | *O. nivara* | *Alcohol metabolism* | 15 | 0 |
| | ***unknown*** | Os11g0483900 | Os11g29350.2 | Mixed | Identical nt | Unknown | 4 | 0 |
| | ***unknown*** | Os11g0484500 | Os11g29400.1 | Or-J[b] &On-I[b] | New J | Unknown | 14 | 27 |
| | ***CHS*** | Os11g0530600 | Os11g32650.1 | Mixed[b] | Mixed | Flavonoid network | 2 | 3 |
| **12** | ***unknown*** | Os12g0108500 | Os12g01760.1 | Or-J &On-I | *O. rufipogon* | Unknown | 1 | 0 |
| | ***unknown*** | Os12g0533700 | Os12g34860.1 | Mixed | Mixed | Unknown | 5 | 3 |
| | ***SDH2(DHQD2)*** | Os12g0534000 | Os12g34874.1 | Or-J &On-I | Mixed | *Shikimate pathway* | 0 | 1 |
| | ***unknown*** | Os12g0534700 | Os12g34920.1 | *O. rufipogon* | *O. rufipogon* | Unknown | 0 | 0 |
| | ***unknown*** | Os12g0578400 | Os12g38920.1 | Or-like | New i | Unknown | 1 | 2 |
| | ***CM2*** | Os12g0578200 | Os12g38900.1 | *O. rufipogon* | *O. rufipogon* | *Shikimate pathway* | 0 | 0 |
| | | | | | | Subtotal | 478 | 278 |
| | | | | | | Total | 756 | |

[a] Gene ID was based on the Nipponbare genome annotated by MSU Rice Genome Annotation Project.
[b] Alignable 5' regions were shorter than 1 Kb.
[c] The gene types largely followed those of Figure 3, with mixed standing for Type 4.
[d] Italic indicates uncharacterized genes, which have protein domains similar to these characterized in other species such as *Arabidopsis thaliana*.
[e] Missing sites in *On* lineage.
[f] Details see Supplemental Fig. 1.
