# Supplementary material for: Early Domestication History of Asian Rice Revealed by Mutations and Genome-Wide Analysis of Gene Genealogies
Source: Rice (N Y). 2022 Feb 15;15:11. doi: 10.1186/s12284-022-00556-6 (PMC8847465; doi:10.1186/s12284-022-00556-6)

Additional file 2

Supplemental Fig. 1. Mapping of mutational events.

A total of 202 genealogies are shown for 101 genes sampled from the genomes of *Oryza sativa* and the close relatives *O. rufipogon* (Or) and *O. nivara* (On). Genes are grouped by chromosomes and numbered in the order as one listed in Supplemental table 1. A gene-genealogy follows the format of Fig. 1. The missing sites of nucleotides are shown in square. Indels are in circles and those causing early stop codons in filled circles.

a. Chromosome 1

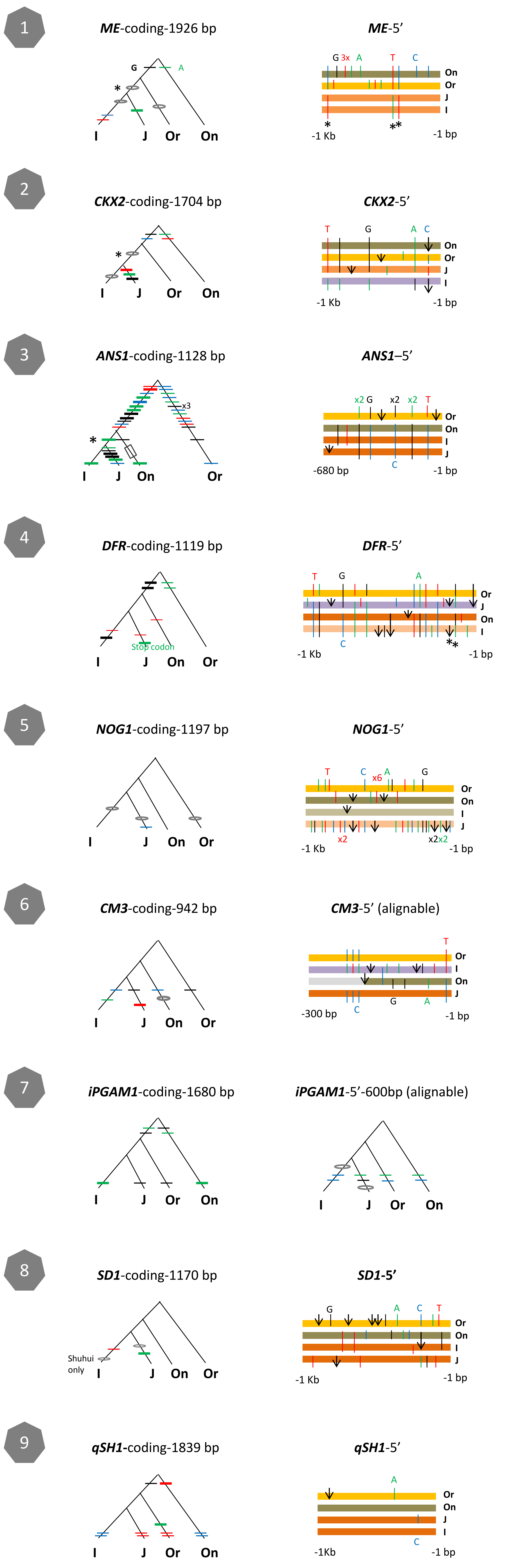

b. Chromosome 2

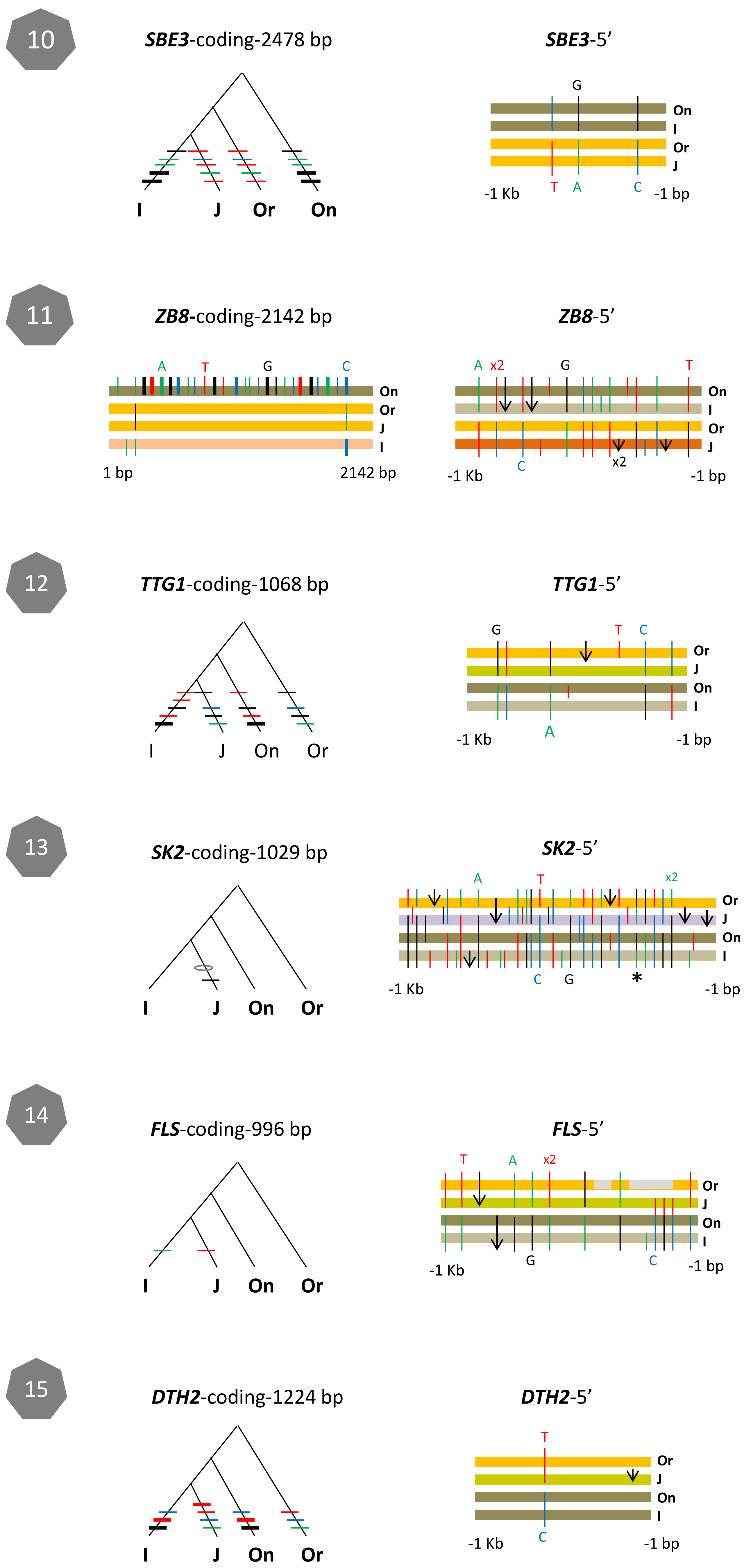

c. Chromosome 3

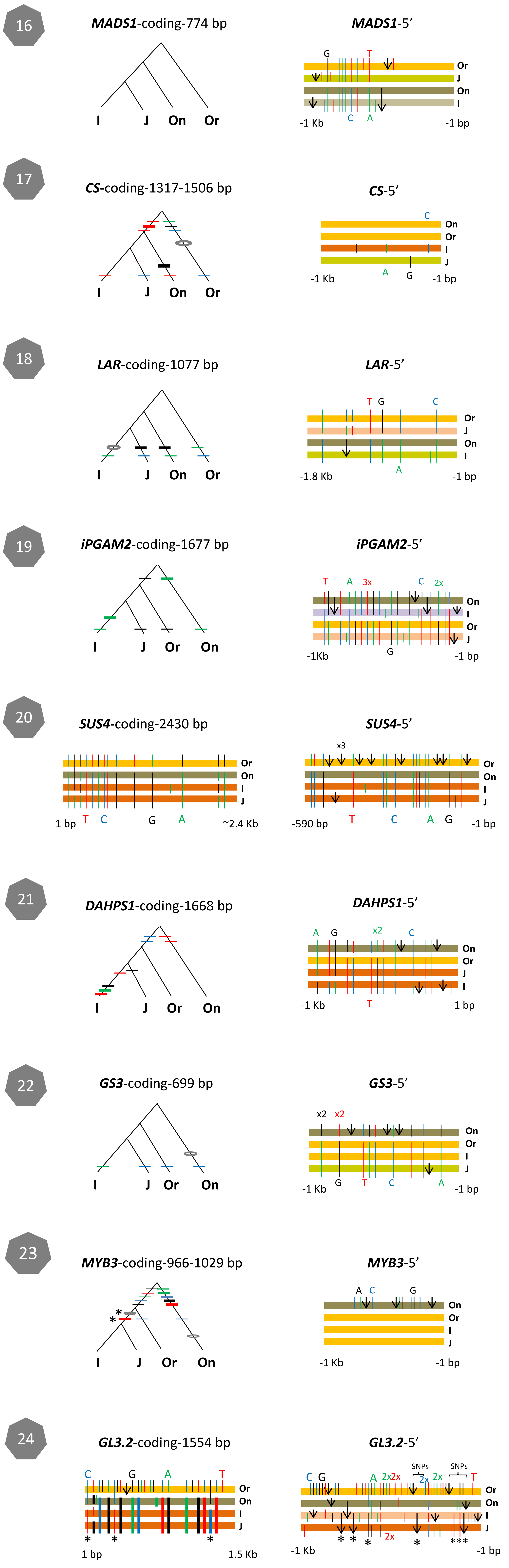

c. Chromosome 3 (cont.)

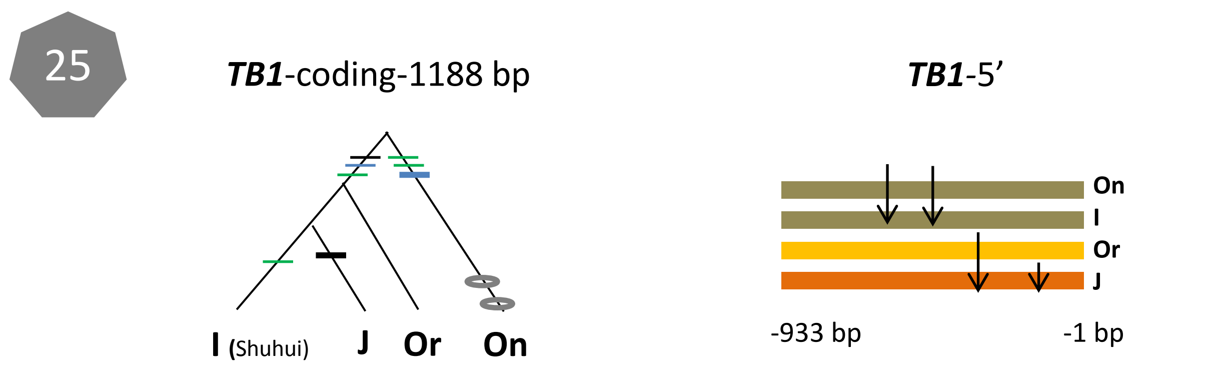

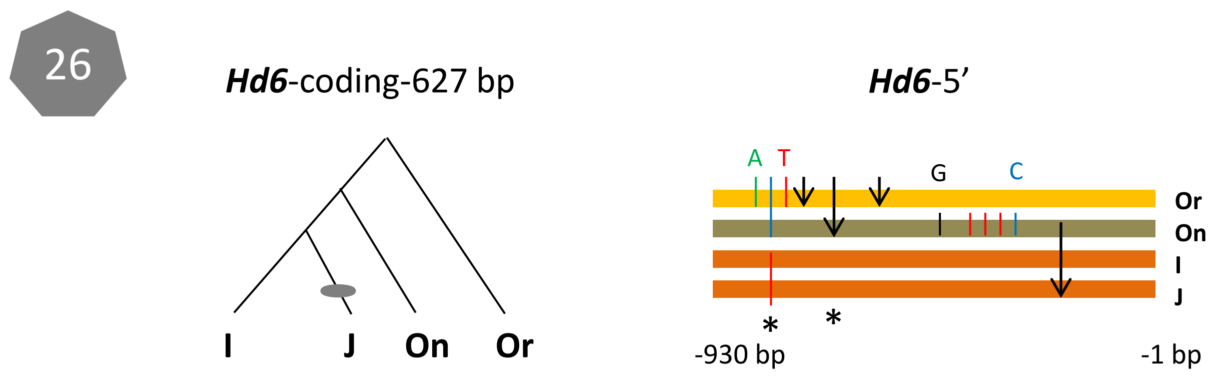

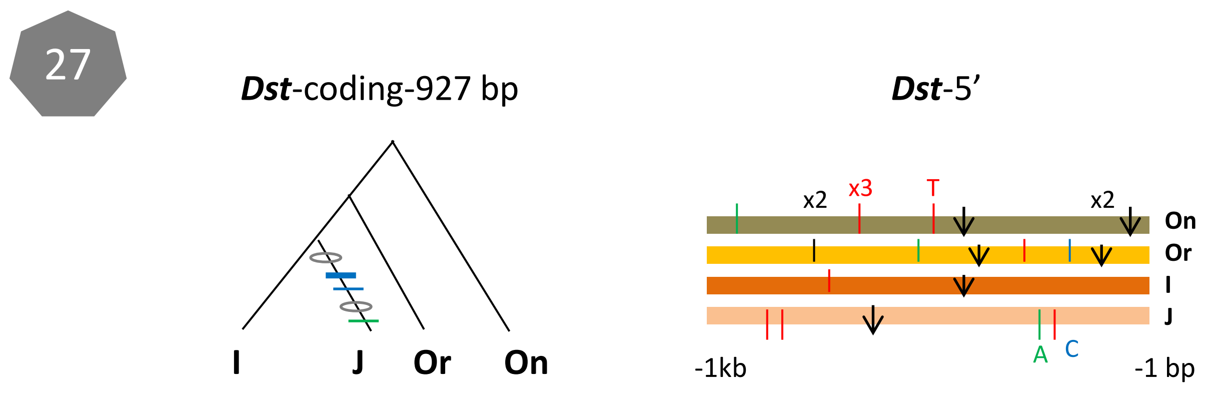

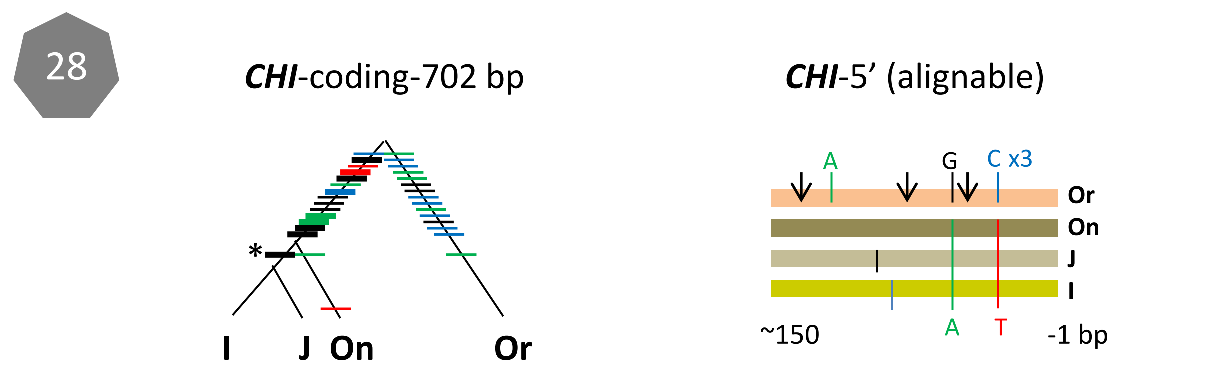

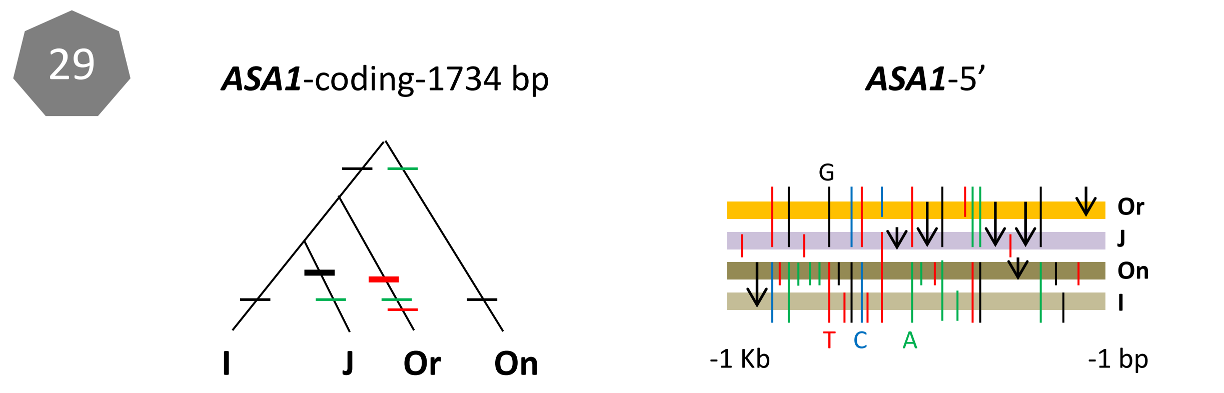

d. Chromosome 4

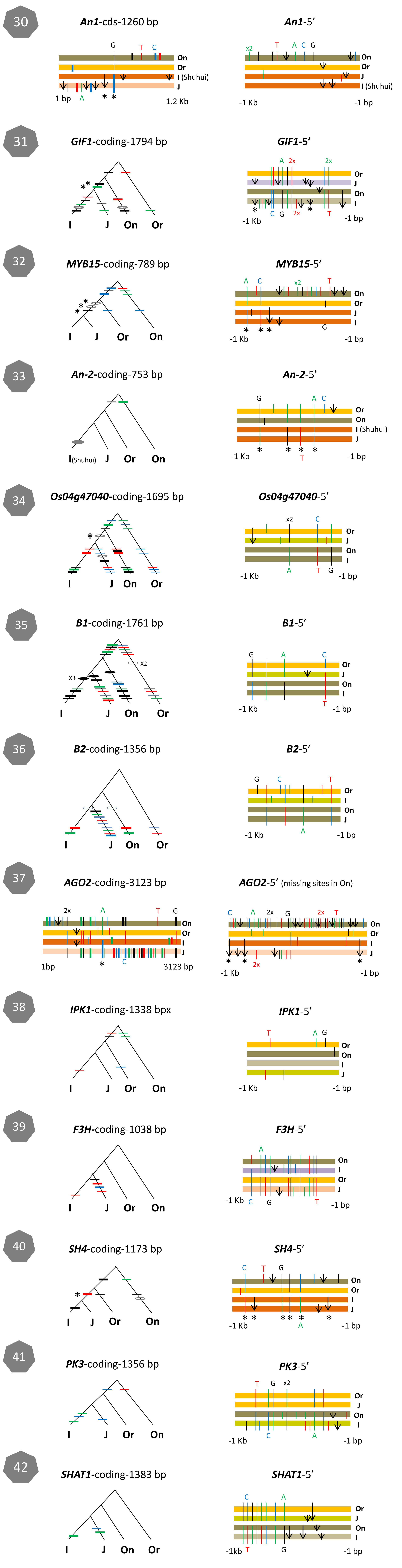

e. Chromosome 5

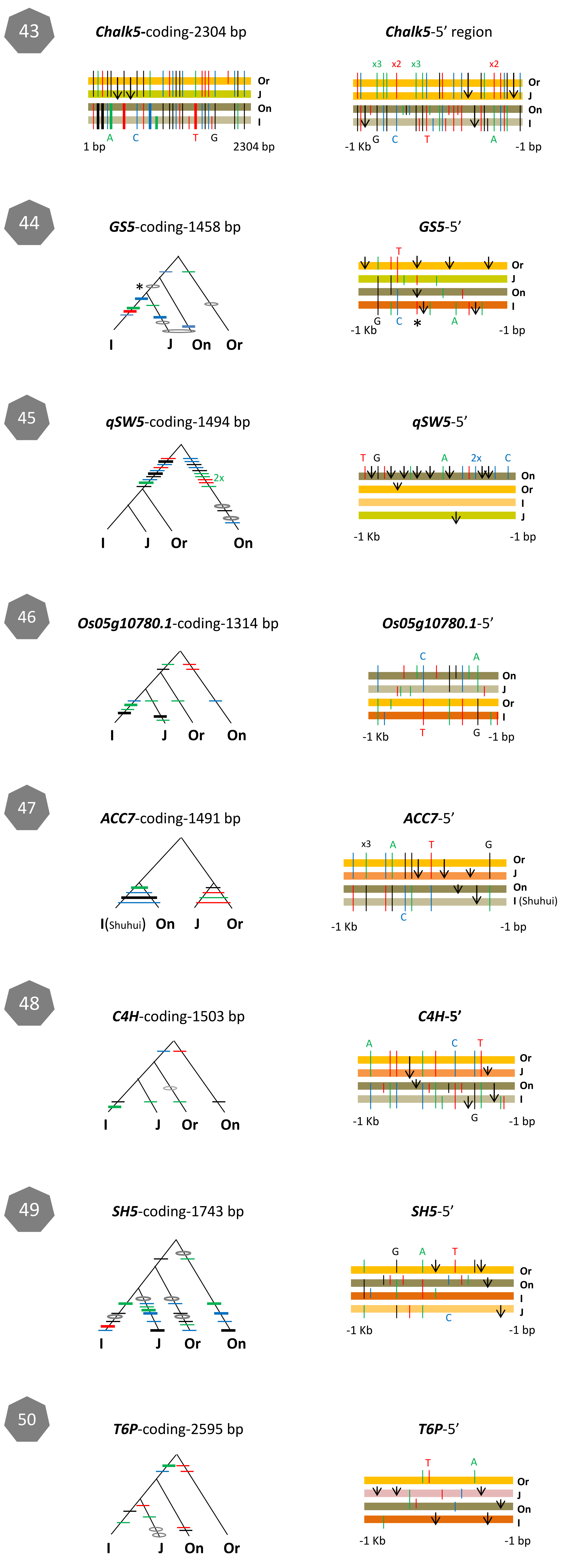

f. Chromosome 6

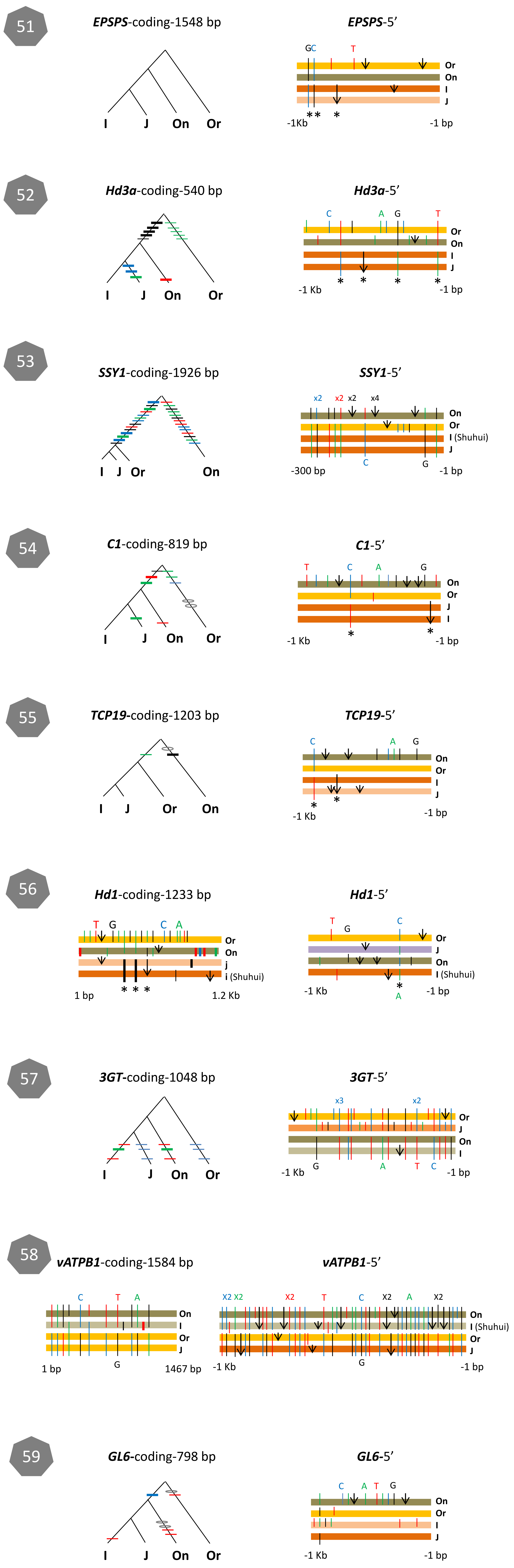

g. Chromosome 7

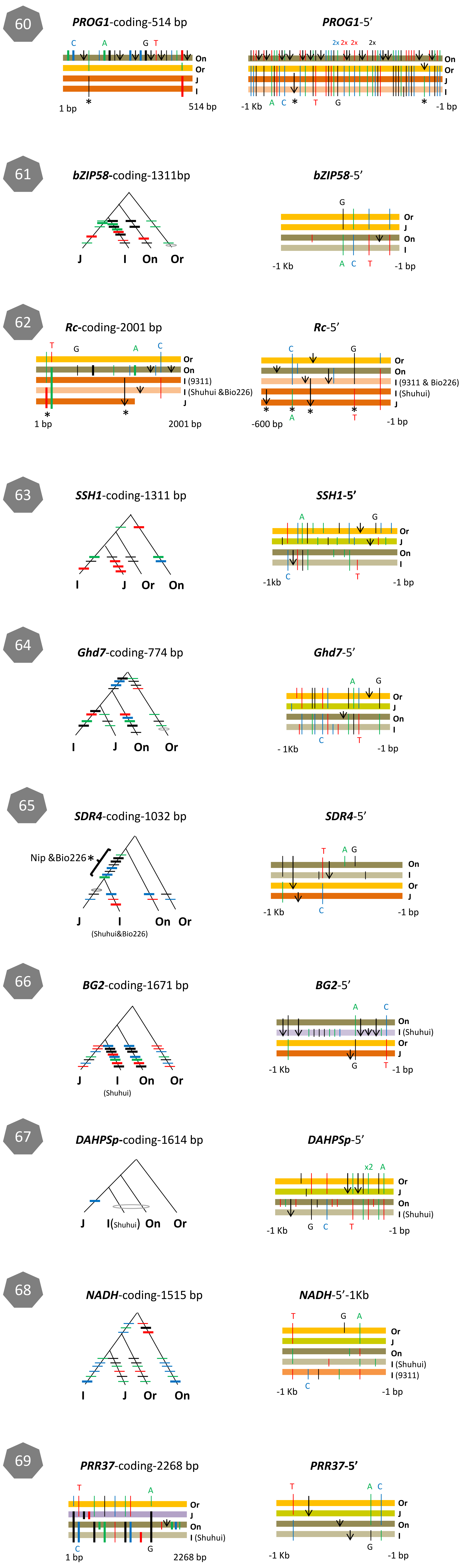

h. Chromosome 8

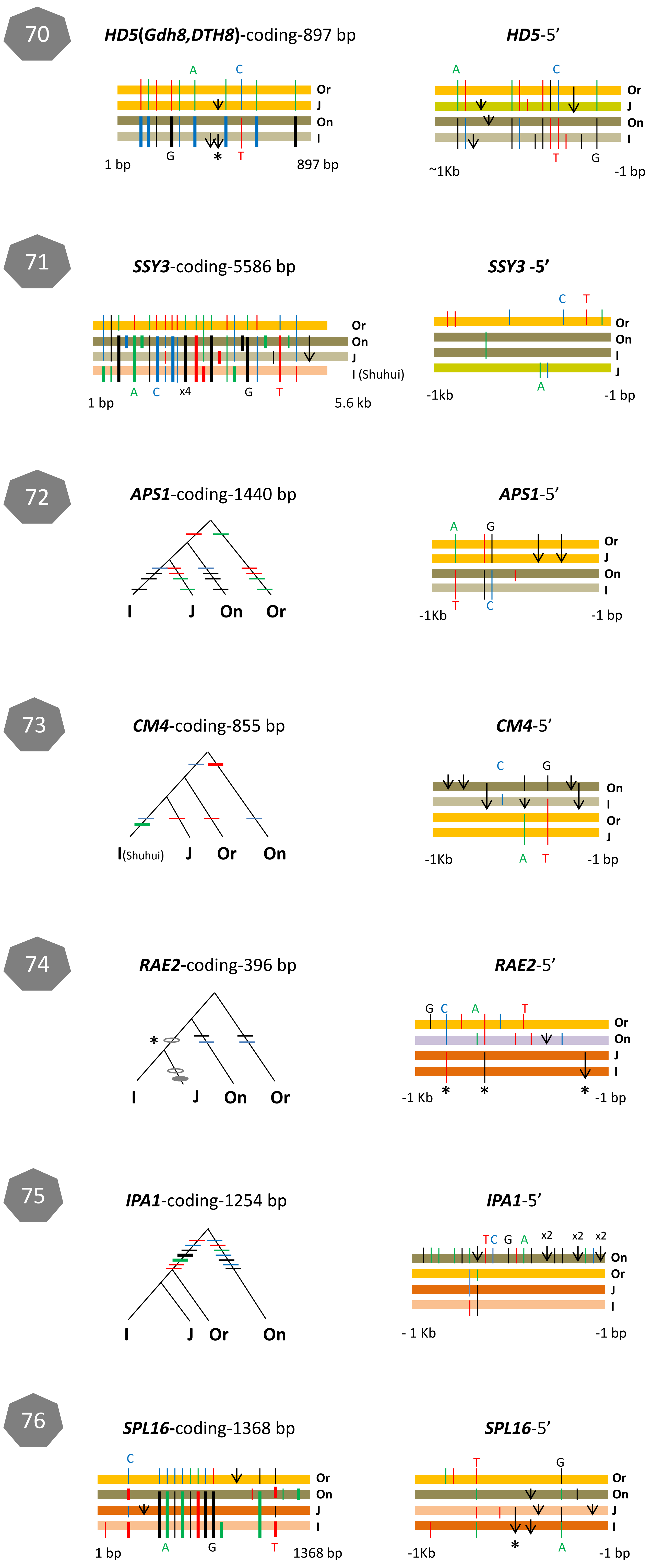

i. Chromosome 9

77

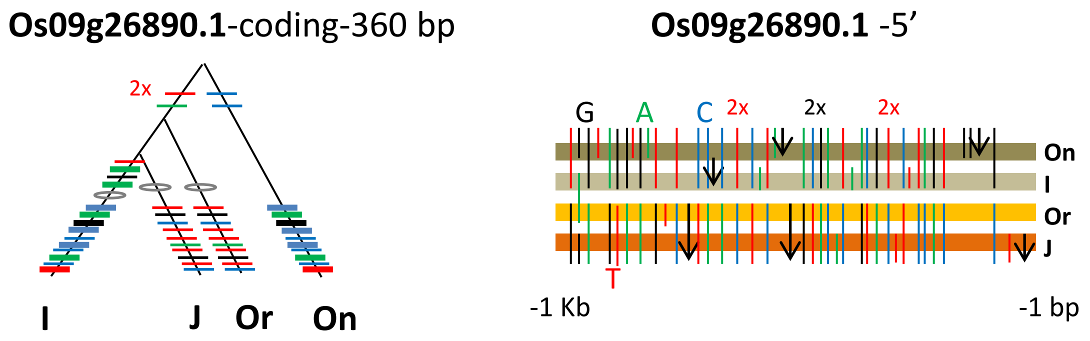

78

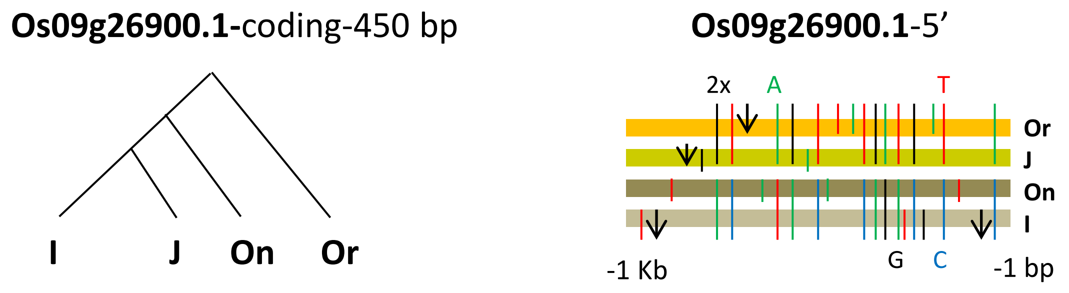

79

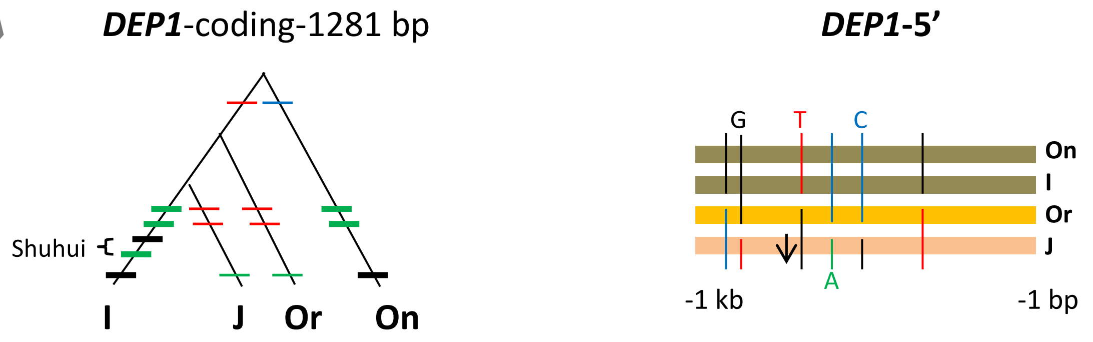

80

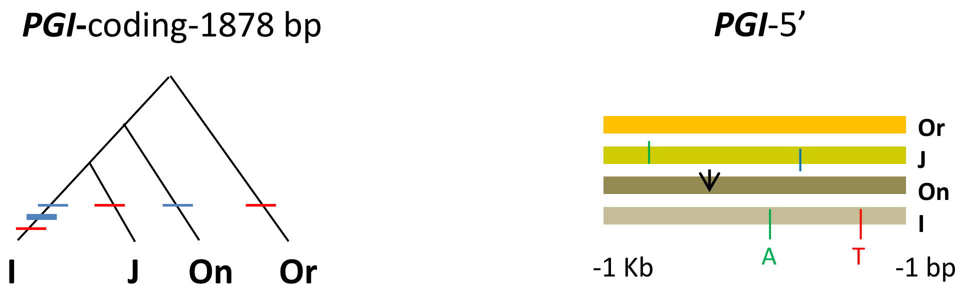

81

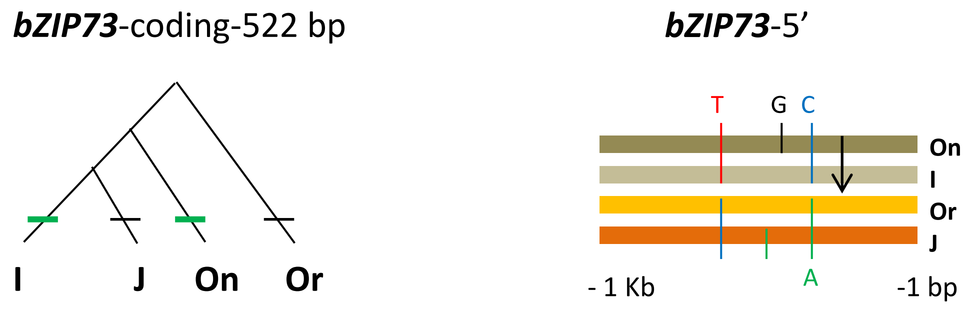

82

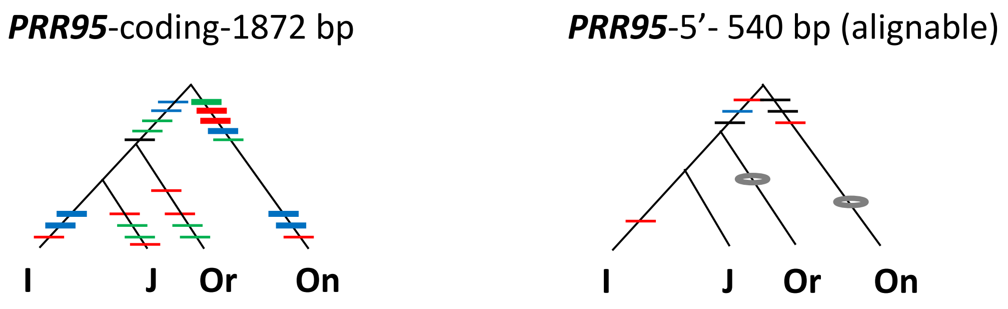

83

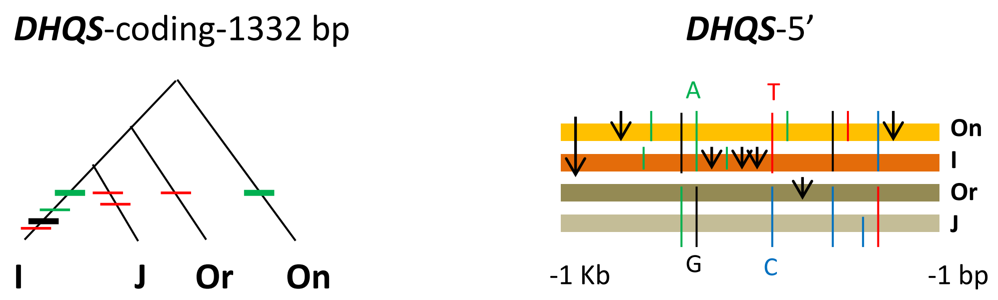

j. Chromosome 10

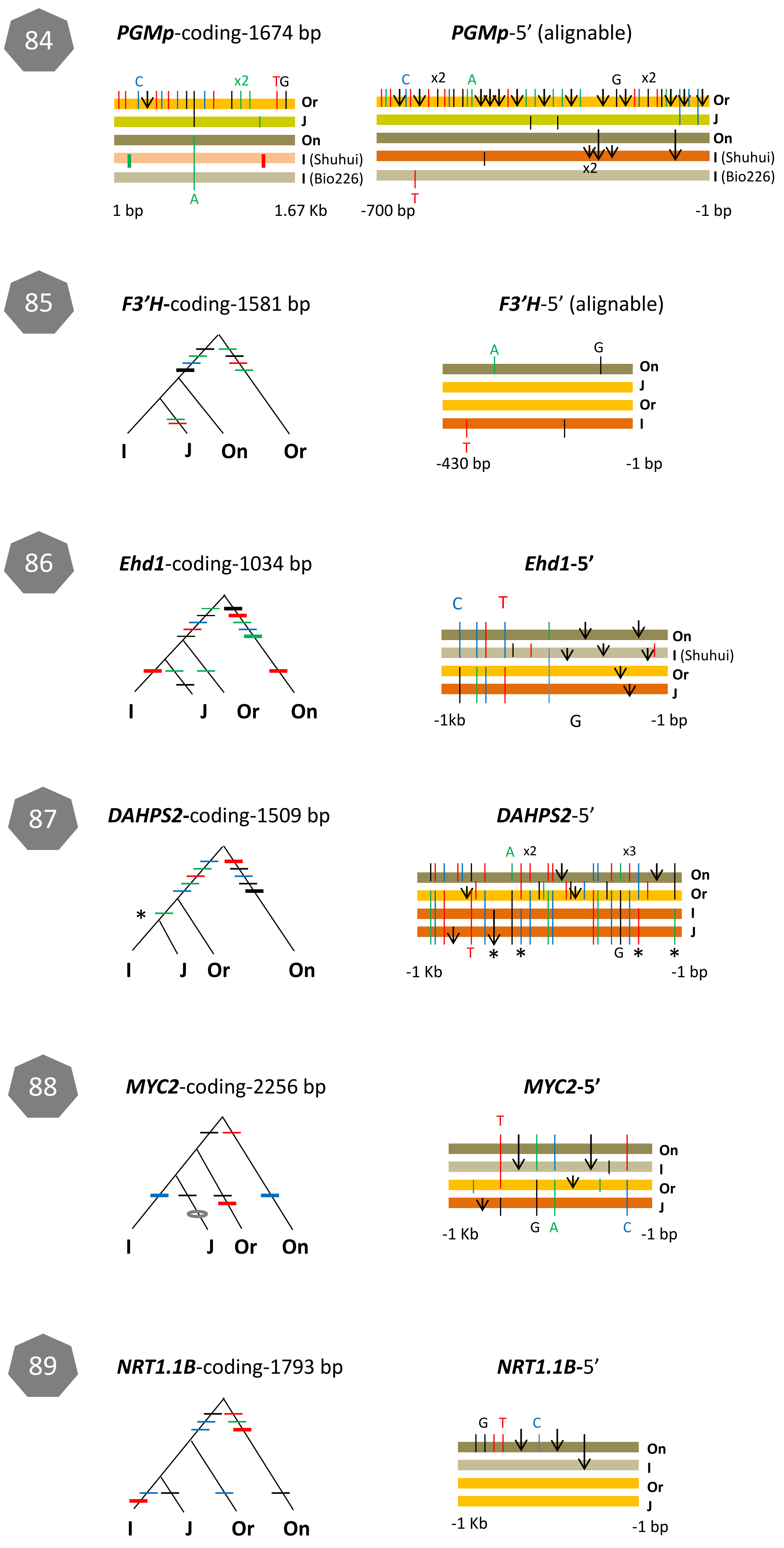

k. Chromosome 11

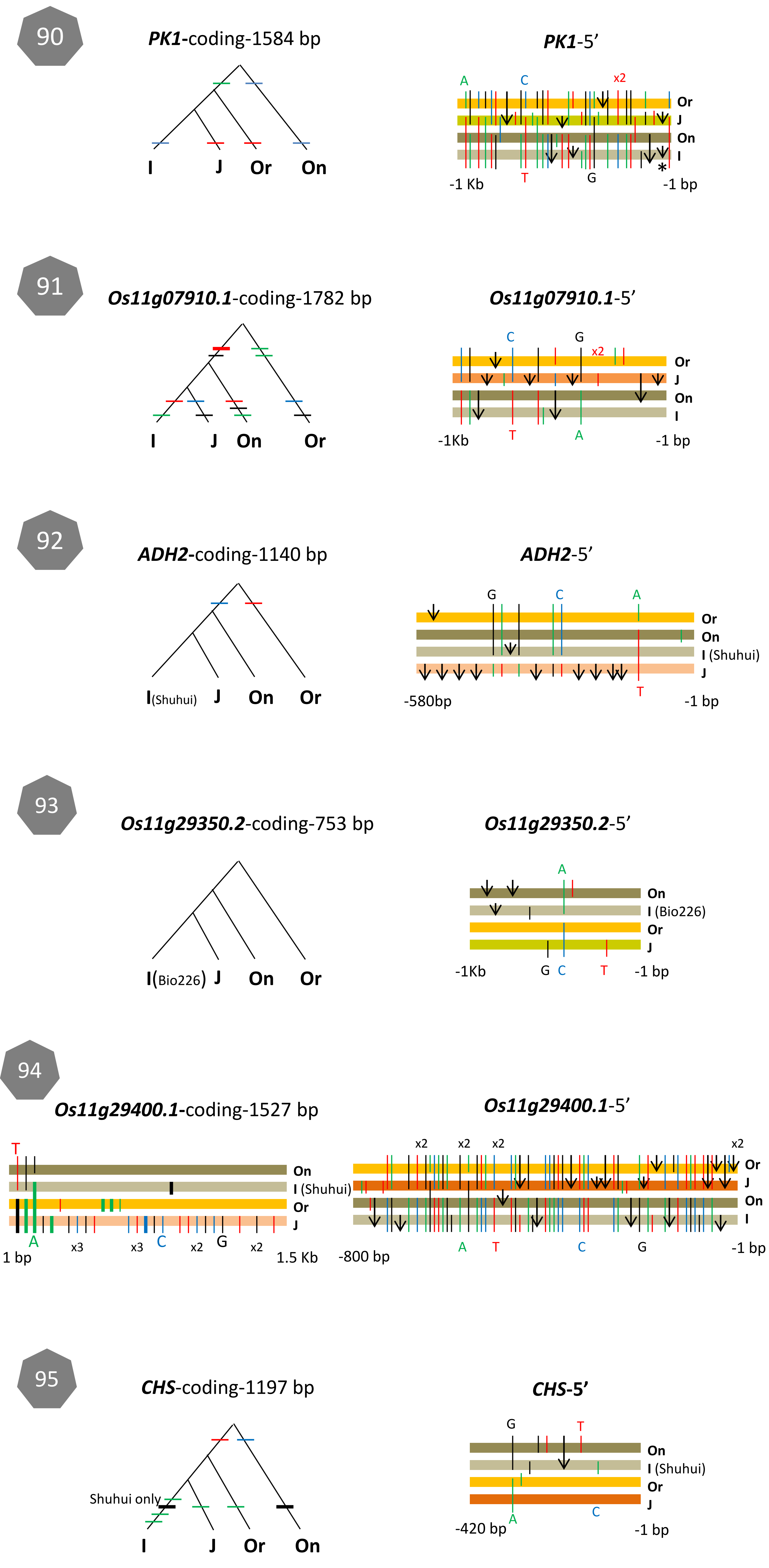

I. Chromosome 12

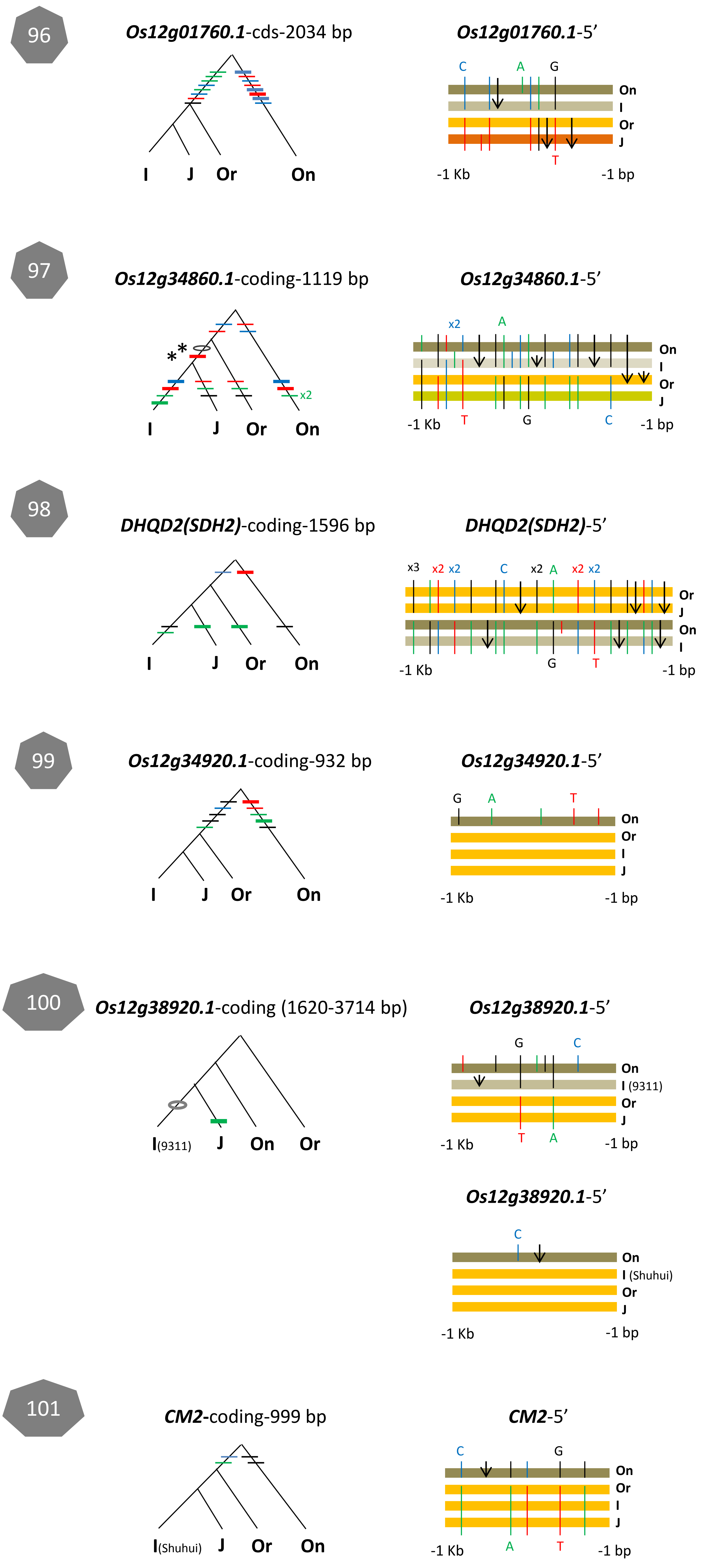

Supplement: Supplementary file 2 — Additional file 2: Fig. S1. Mapping of mutational events. a Chromosome 1. b Chromosome 2. c Chromosome 3. d Chromosome 4. e Chromosome 5. f Chromosome 6. g Chromosome 7. h Chromosome 8. i Chromosome 9. j Chromosome 10. k Chromosome 11. l Chromosome 12. [file 12284_2022_556_MOESM2_ESM.pdf]
