# Supplementary material for: Early Domestication History of Asian Rice Revealed by Mutations and Genome-Wide Analysis of Gene Genealogies
Source: Rice (N Y). 2022 Feb 15;15:11. doi: 10.1186/s12284-022-00556-6 (PMC8847465; doi:10.1186/s12284-022-00556-6)
Supplement: Supplementary file 3 — Additional file 3: Table S2. A list of 30 loci showing early and later mutations in rice. [file 12284_2022_556_MOESM3_ESM.pdf]

Additional file 3

Supplemental Table 2. A list of 30 loci showing early and later mutations in Asian rice.

	Number of mutations shared between *Indica* and *Japonica*[b]		Number of mutations specific to *Indica*[c]		Number of mutations specific to *Japonica*[c]	
**Rice locus[a]**	**5'**	**Coding**	**5'**	**Coding**	**5'**	**Coding**
SH4	6	1(nons)		1(nons)	1(idl)	
An-1(Awn-1)		2(1 non,idl:1 aa)	1(idl)	2(idl:>3 aa)	3(1 idl)	7(2 nons, 3 idl:>3 aa)
An-2(Awn-2)	4			1(idl:>3 aa)		
Hd5(Gdh8)		1(idl:1 aa)	4(1 idl)	1(idl:1 aa)	2(1 idl)	
GL3.2	7(3 idl)	3(1 nons)	11(3 idl)		3	
GIF1	2(idl)	2(2 nons)	7(2 idl)	5(1 nons, 1 idl:2 aa)	2(2 idl)	2
AGO2	3(3 idl)	1(1 nons)	2	4(3 nons)	7(2 idl)	19(9 nons)
GS5	1(idl)	1(idl:1 aa)	7(2 idl)	4(3 nons)	2	3(1 nons, 1 idl:2 aa)
PROG1	2(1 idl)	1	1			
TCP19	2(1 idl)				2(2 idl)	
CKX2		1(idl:2 aa)	4	1(idl:2 aa)	3(1 idl)	3(3 nons)
Hd3a	4 (1 idl)					3(3 nons)
Hd1	1	3(2 nons,1 idl:2 aa)	2(1 idl)	2(1 nons, 1 idl:>3 aa)	1(idl)	1(nons)
Hd6	2(1 idl)					1(idl: stop codon)
C1	2(1 idl)					1(nons)
CHI[#]		1(nons)	1		1	
DFR	2(1 idl)		4(2 idl)	2(1 nons)	6(2 idl)	2(1 nons)
ANS1		1(nons)		1(nons)	1(idl)	6(3 nons)
Os04g47040		1(idl:5 aa)		1(idl:5 aa)	2	2(1 nons, 1 idl:6 aa)
Rc[#]	3(1 idl)	2(1 nons, 1 idl:stop codon)	1(idl)	1(idl:>3aa)		
MYB3		2(1 nons, 1 idl:3 aa)				
MYB15	3(1 idl)	3(2 idl:5 aa)			1	
SK2	1		8(1 idl)		8(2 idl)	
DAHPS2	4	1			1(idl)	
PK1	1(idl)		3(1 idl)		6(2 idl)	
EPSPS	3(1 idl)		1(idl)			
ME	3	1(idl:2 aa)		3(1 idl:2 aa)		1(nons)
Os12g34860.1		2(1 nons, 1 idl:stop codon)	4(1 idl)	1(nons)		
RAE2	3	1(idl:stop codon)				2(2 idl:2 aa)
SPL16	1(idl)		1	2(1 nons)	3(2 idl)	1 (idl:1 aa)
**Subtotal**	**60**	**31**	**62**	**32**	**55**	**54**

[a] Loci in bold were detected under positive selection during Phase I.
[b] Mutations contributed to positive selection were underlined, including nonsynonymous substitutions (nons) and indels (idl). Amino acid (aa) changes introduced by each indel was listed after comma. Synonymous substitutions (syn) were not shown for simplicity but included in the mutation number.
[c] Underlined mutations are under positive selection in Phase II.
[#] Genes of shorter sequences
