# Supplementary material for: Early Domestication History of Asian Rice Revealed by Mutations and Genome-Wide Analysis of Gene Genealogies
Source: Rice (N Y). 2022 Feb 15;15:11. doi: 10.1186/s12284-022-00556-6 (PMC8847465; doi:10.1186/s12284-022-00556-6)
Supplement: Supplementary file 4 — Additional file 4: Table S3. Estimation of background-mutation rates from intron mutations of four genomes (Or, On, Indica (I) and Nipponbare (J)). [file 12284_2022_556_MOESM4_ESM.pdf]

Additional file 4

Supplemental Table 3. Estimation of background-mutation rates from intron mutations of four genomes (*Or*, *On*, *Indica* (I) and Nipponbare (J)).

Locus	Intron surveyed	Number of shared mutation between I and J	Mutations specific to I	Mutations specific to J	Length of intron[a] (bp)
SH4	Intron 1	3	0	0	863
AN-2	Intron 1	2	0	0	965
AN-1	Intron 3	0	0	0	832
DAHPS2	Intron 1	1	0	0	1001
SSY3	Intron 1	0	1	1	1095
Rc	Intron 2	1	0	0	2717
GS5	Intron 4	0	0	0	939
Chalk5	Intron 1	0	6	0	1561
Lc	Intron 1	0	0	1	2487
	**Sum**	7	7	2	12460
	**Mutation Rate**[b]	0.56	0.56	0.16	

[a] The length of the longest intron in the case of unequal intron lengths among orthologs.
[b] The unit is number of mutations per 1K nucleotides over the historical period implicated.
