# Supplementary material for: Early Domestication History of Asian Rice Revealed by Mutations and Genome-Wide Analysis of Gene Genealogies
Source: Rice (N Y). 2022 Feb 15;15:11. doi: 10.1186/s12284-022-00556-6 (PMC8847465; doi:10.1186/s12284-022-00556-6)
Supplement: Supplementary file 5 — Additional file 5: Table S4. Distribution of eight gene types (shown in Fig. 3) among 101 sampled loci of O. sativa. [file 12284_2022_556_MOESM5_ESM.pdf]

Additional file 5

Supplemental Table 4. Distribution of eight gene types (shown in Fig. 3) among 101 sampled loci of *O. sativa*.

Gene type	Affinity	Gene region		Source
		5'	Coding	
Type 1	Ancient	0	13	Ancient
Type 2	*On*-like	6	10	*O. nivara*
Type 3	*Or*-like	14	16	*O. rufipogon*
Type4	Mixed	46	33	Bi-specific
Type 5	*Or*-J & *On*-I	18	11	Bi-specific
Type 6	*Or*-I & *On*-J	1	1	Bi-specific
Type 7	*Or(On)-On (Or)*	1	1	Bi-specific
Type 8	New	14	17	New
