# Supplementary material for: Early Domestication History of Asian Rice Revealed by Mutations and Genome-Wide Analysis of Gene Genealogies
Source: Rice (N Y). 2022 Feb 15;15:11. doi: 10.1186/s12284-022-00556-6 (PMC8847465; doi:10.1186/s12284-022-00556-6)
Supplement: Supplementary file 6 — Additional file 6: Data 1. Plant height of F2 population in the field experiment 1. [file 12284_2022_556_MOESM6_ESM.pdf]

Additional file 6

Field data 1 Plant heights of F2 population from the field experiment 1.

Field Experiment 1

Height (cm) /plant								
70	95	105	110	125	130	135	145	158
70	95	105	112	125	130	135	145	160
70	98	105	115	125	130	135	145	160
75	98	105	115	125	130	138	145	160
75	98	108	115	125	130	138	145	160
75	98	110	115	125	130	140	145	160
80	100	110	115	125	130	140	145	160
80	100	110	115	125	130	140	145	160
80	100	110	115	125	130	140	145	160
80	100	110	115	125	130	140	145	160
80	100	110	120	125	130	140	145	160
82	100	110	120	125	130	140	145	160
85	100	110	120	125	130	140	145	160
85	100	110	120	125	130	140	148	160
90	100	110	120	125	130	140	148	160
90	100	110	120	125	130	140	148	165
90	100	110	120	125	132	140	148	165
90	100	110	120	128	132	140	150	165
90	100	110	120	128	135	140	150	170
90	100	110	120	130	135	140	150	170
90	100	110	120	130	135	140	150	170
90	100	110	120	130	135	140	150	170
90	100	110	120	130	135	140	150	170
90	100	110	120	130	135	140	150	170
92	102	110	120	130	135	140	150	170
92	102	110	120	130	135	140	150	175
94	105	110	120	130	135	140	150	180
95	105	110	120	130	135	140	150	180
95	105	110	120	130	135	140	150	180
95	105	110	120	130	135	140	150	182
95	105		122	130	135	140	150	
95	105		122	130	135	140	150	
95	105		123	130		140	155	
	105			130		140	155	
	105			130		140	155	
						140	155	
						140	155	
						142	155	
						142		

Data for Figure 4D

Height	count	mean
70-90	14	77.64
90-110	59	98.42
110-130	77	117.4
130-150	104	136.89
150-170	39	155.72
170-190	12	173.92
total	305	
