# Supplementary material for: Early Domestication History of Asian Rice Revealed by Mutations and Genome-Wide Analysis of Gene Genealogies
Source: Rice (N Y). 2022 Feb 15;15:11. doi: 10.1186/s12284-022-00556-6 (PMC8847465; doi:10.1186/s12284-022-00556-6)
Supplement: Supplementary file 7 — Additional file 7: Data 2. Morphological data in the field experiment 2. [file 12284_2022_556_MOESM7_ESM.pdf]

Additional file 7

Field data 2. Morphological data from the field experiment 2.

Anther length (mm) measured on 20190828

Plant	Anther1	Anther2	Anther3	Anther4	Anther5	Anther6	Anther7	Anther8
1	2.4	2.6	2.2	2.3	2.28	2.4		
2	2.63	2.56	2.6	2.3	2.48	2.62		
3	2.4	2.5	2.42	2.66	2.44	2.46		
4	2.4	2.2	2.24	2.16	2.22	2.4		
5	2.7	2.8	2.9	2.92	2.8	2.45		
6	2.12	2.4	2.3	2.4	2.56	2.4		
7	2.1	2.16	2.2	2.3	2.24	2.48		
8	2.2	2.3	2.1	2.12	2.16	2.1		
9	2.3	2.28	2.4	2.5	2.36	2.6		
10	2.8	2.6	2.7	2.5	2.46	2.5		
11	2.8	2.7	2.71	2.6	2.7	2.6		
12	2.8	2.5	2.6	2.2	2.6	2.6		
13	2.8	2.9	3.1	3	2.9	2.7		
14	2.8	2.9	2.76	2.9	2.62	2.8		
15	2.1	2.18	2.4	2.46	2.3	2.32		
16	2.2	2.4	1.7	2.5	2.18	2.26		
17	2.3	2.34	2.4	2.38	2.2	2.4		
18	2.4	2.5	2.56	2.4	2.28	2.3		
19	2.4	2.6	2.58	2.5	2.88	2.72		
20	2.5	2.56	2.1	2.48	3	2.6	2.7	
21	2.76	2.6	2.64	2.1	2.48	2.56		
22	3	2.5	2.6	2.58	2.7	2.64		
23	2.9	2.5	2.7	2.6	2.56	2.5		
24	2.6	2.8	2.9	2.68	2.6	2.5		
25	2.54	2.2	2.5	2.48	2.18	2.3		
26	2.32	2.36	2.5	2.3	2.28	2.26		
27	2.3	2.6	2.74	2.8	2.3	2.58	2.9	2.8
28	2.3	2.15	2.08	2.12	2.36	2.26		
29	2.4	2.3	2	2.2	2.36	2.18	2.06	
30	2.4	2.38	2.32	2.01	2.2	2		
31	2.26	2.62	2.48	2.56	2.36	2.28		
32	2.3	2.4	2.38	2.34	2.4	2.6		
33	2.9	2.88	2.56	2.7	2.8	2.78		
34	2.22	2.28	2.6	2.16	2.42	2.3		
35	2.3	2.1	2.4	2.5	2.48	2.66		
36	2.6	2.56	2.5	2.8	2.7	2.74		
37	2.6	2.4	2.2	2.7	2.9	2.55		
38	2.38	2.5	2.52	2.6	2.3	2.25		
39	2.4	2.25	2.36	2.52	2.7	2.72		
40	2.3	2.4	2.36	2.28	2.36	2.28		

Data for Figure 4E

Bin	Counts
1.6-1.8	1
1.8-2.0	2
2.0-2.2	67
2.2-2.4	128
2.4-2.6	103
2.6-2.8	79
2.8-3.0	50
3.0-3.2	24
3.2-3.4	12
total	466

Anther length (mm) measured on 20190904

Plant	Anther1	Anther2	Anther3	Anther4	Anther5	Anther6	Anther7	Anther8	Anther9
1	3.2	2.6	3.14	2.9	3.16	2.88	2.76	2.9	3.2
2	2.66	2.42	2.6	3.2	2.9	2.8	2.76	2.6	2.56
3	3.1	2.5	2.7	2.9	2.5	2.8	2.6	2.72	2.9
4	3.1	3.12	2.78	3.06	3	3.02	3.34	2.88	2.96
5	2.94	2.8	2.78	2.56	2.78	2.52	2.9	2.6	2.7
6	2.78	2.2	2.1	2.56	2.82	2.94	2.4	2.72	2.44
7	2.56	2.4	2.36	2.68	2.7	2.3	2.32		
8	3.1	3.4	3.02	3.2	3.28	3.3	3.3	3.14	2.86
9	3.2	3.3	2.8	2.9	3.38	2.92	3	3.12	2.96
10	2.9	2.86	2.96	2.88	2.74	2.8	2.6	2.82	2.48
11	3.16	3.2	3.18	2.9	3.4	2.78	2.7	2.8	3.16
12	3.26	2.74	3.18	3.2	3.22	3.3	3.22	2.88	2.9
13	2.38	2.6	2.4	2.2	2.1	2.3	2.24	2.28	2.3
14	2.36	2.2	2.1	2.22	2.18	2.2	2.3	2.26	2.18
15	2.08	2.3	2.4	2.4	2.38	2.2	2.3	2.4	2.08
16	2.4	2.2	2.18	2.16	2.4	2.36	2.16	2.2	2.3
17	2.16	2.3	2.32	2.2	2.16	2.18	2.14	2.1	2.24
18	2.2	2.22	2.16	2.2	2.16	2.12	2.1	2.16	2.18
19	2.9	2.7	2.68	2.64	2.8	2.7	2.5	3.2	2.88
20	2.4	2.36	2.2	2.28	2.36	2.4	2.3	2.42	2.48
21	2.4	2.6	2.36	2.38	2.4	2.3	2.58	2.5	2.6
22	2.52	2.6	2.38	2.88	2.82	2.62	2.3	2.44	2.22
23	3	2.66	2.56	2.5	2.4	2.7	2.58	2.72	2.78
24	2.8	3.1	2.78	2.92	2.56	2.5	2.9	2.8	2.88
25	2.2	2.8	2.45	2.4	2.22	2.38	2.3	2.28	2.2

Field data 2 (conti.)

Field experiment 2 – Degree of exsertion of panicle

Accessions[†]	Tiller1[§]	Tiller2	Tiller3	Accessions	Tiller1	Tiller2	Tiller3	Accessions	Tiller1	Tiller2	Tiller3	Accessions	Tiller1	Tiller2	Tiller3
1	5	4.5	2	13	14	12	13.9	24	9.9	9.8	11.1	34	7.4	8	5.5
	4	<0	<0		12.8	10.1	10.7		6.5	5.9	5.7		5.3	7.1	2.5
	2	<0	2.5		12.1	13.2	10.1		1.3	0.6	0.4		7.3	2.1	5
	2	0.5	<0		5.3	6.2	7.9		6	6.5	5.8		8.8	7.6	8
	2	<0	0.5		7	9.5	8.2		7.2	7.8	6.1		5.9	5.2	4.7
2	6.5	6.5	3.5	14	<0	3.9	4.3	25	3.6	6.3	4.8	35	4.8	3.9	2.1
	6.5	4	4		3	2.1	2		10	7.6	13.2		3.1	4.5	4.2
	1	2	2.5		4.3	<0	<0		8.6	6.3	7.2		3.8	2.1	5
	8	2.5	3.5		1.5	1.5	0.5		4.2	3.6	6.5		0	2.8	4.5
	7	3	9		1.5	<0	<0		5.8	7.1	6.3		5.7	<0	5.4
	4.3	6.4	5.6	15	7.1	6.9	7	26	8.6	7.7	7.5	36	1.1	3.5	1.5
	8.1	11	9.6		4.9	5.3	4.9		9.2	8.4	3.8		<0	2	5.5
3	<0	1.3	<0		7.9	5.3	6.8		3.6	3.8	1.8		1.1	5	5.3
	0.6	4.8	1.4		4.9	4.2	3.5		6.3	5.6	2.8		2.2	3.9	1.8
	0.9	0.7	0.6		3.7	2.5	3.8		2	<0	<0		<0	3.4	<0
	<0	<0	<0	16	5.4	7.8	7.9	27（North）	6.2	3.6	1.6	38	<0	<0	<0
	6.5	5	4.6		0	0	0.5		3	6.6	7.8		<0	<0	<0
4	0.5	3.6	4		5.1	5.1	3.9		2.9	2	1.8		<0	<0	<0
	0.7	3.2	3		3.2	3.4	4.9		9.5	11.4	10.7		<0	<0	<0
	1.4	5.5	4.7		7.3	3.9	6.9		15.3	12	13.2		<0	<0	<0
	<0	3.5	<0	17	3.9	4.6	5.8	27（South）	4	5	5.2	39	1.2	1.3	2.7
	<0	4.5	<0		7	6.7	6.6		4.4	4.4	4.3		<0	<0	<0
5	3.5	1.1	4.8		7.9	7.6	8.3		13.2	15.1	14.2		<0	<0	1.1
	3	2	1		7	8.4	5.3		8.1	10.9	10.4		<0	<0	<0
	4.4	3.1	<0		7.9	8.7	4.2		12.3	12.8	11.9		<0	<0	<0
	3.6	3.1	4	18	5.7	5.2	5.9	28（North）	<0	<0	<0	41	2.5	1	3.8
	3.2	1.5	<0		3.3	5.7	7.3		<0	<0	<0		4.4	4.5	2.1
6	4	0.5	0.5		5.7	4.2	5.5		1.2	<0	<0		6.6	7.5	5
	3.5	3	1.5		3.9	3.6	3.9		<0	<0	<0		5	5	5.9
	3.5	0.5	3		5.7	5.2	6.1		<0	<0	<0		4.8	7	5.2
	3	1.5	0.5	19	3.1	5.5	6	28（South）	<0	<0	<0	C2	5.9	6	2.1
	3	4.5	3		6.6	3.9	3.2		<0	<0	<0		<0	<0	<0
7	<0	<0	<0		3.9	4.1	0.5		<0	<0	<0		1.9	<0	4.5
	<0	<0	<0		3.9	4.3	6.2		<0	<0	<0		3	3	3
	<0	<0	<0		5.5	3.2	2.6		<0	<0	<0		<0	<0	<0
	<0	<0	<0	20	5.2	4.3	5.4	29	<0	<0	<0	C3	8.2	6	3.6
	<0	<0	<0		5.8	4.6	5.3		<0	<0	<0		9.5	6.9	7.5
8	2	1.5	3		4.5	3.2	4.6		<0	<0	<0		4.8	8.2	6.7
	1	<0	1		6	6.7	6.1		<0	<0	<0		1.2	<0	3.5
	1.5	0.5	<0		5.7	4.9	4.1		<0	<0	<0		9.1	1.1	9.8
	3.5	2	0.5	21	5	7.8	5.2	30	3.3	4	1.8	C4	6.3	4.9	9.9
	2.5	2	1.5		6.9	8.1	5.3		1.2	4.3	5.4		7.9	1	7.9
9	2	1.5	<0		3.1	4.6	2.8		3.9	2.4	5.2		7.1	7.4	4.9
	2	1	2.5		5.2	4.8	5.6		1.9	1.8	2.4		6.2	11.2	8.6
	1.5	0.5	1.5		5.4	5.5	6.6		1.1	2.3	2.2		11.1	8.9	7.2
	0.5	0	0.5	22	8.4	7.6	5.9	31	1.2	2.4	4.1	C5	<0	<0	<0
	1	0.5	2		5.7	5	6.5		4.7	3.5	7.5		0.6	<0	1.6
10	5.5	3.8	3.4		1.9	4.2	6.1		5.4	5.9	2.3		<0	<0	<0
	3.9	2.5	2		5.2	4.1	4.1		1	<0	<0		<0	<0	<0
	5.2	3.3	<0		8.4	5.2	8		0.9	<0	3.9		<0	<0	<0
	0.5	1.5	<0	23	3.2	2.5	2.3	32	4.9	5.2	3.4	C6	5.9	0.3	2.2
	5	6	3.2		1.8	0.5	<0		5.6	5.2	1.3		0.5	1.5	<0
12	6	7	7.5		1.1	2.8	2.2		4.8	3.8	0.5		1.6	<0	1.3
	9	9.2	9		1.6	4.3	2.1		3.3	5.2	3.2		<0	1.9	<0
	9.2	4.2	9.2		2.2	<0	<0		3.1	5	2.1		<0	<0	1
	5.7	6.3	4.9					33	<0	<0	<0	C7	1.5	0.9	1.8
	7.2	4	6.3						<0	<0	<0		0.9	0.6	0.5
									<0	<0	<0		2.2	2.6	1.8
									<0	<0	<0		<0	<0	<0
									<0	<0	<0		<0	<0	<0

[†] Accessions 1-38 were from filial generations of the cross between an Indica (Jixuenuo) and Japonica (Heidao). 39, 41, C2-C7 were randomly chosen from four Indica, two Japonica, and their crossed progeny.
[§] Values labeled <0 suggest a contracted panicle within the sheath of flag leaf.

Data for Figure 4F

Length (cm)	Counts	Mean
<0	175	<0
0-2	95	1.1
2-4	133	2.96
4-6	143	4.96
6-8	85	6.9
8-10	37	8.8
10-12	13	10.82
12-14	11	12.79
14-16	4	14.65

N	696
Accessions	46
Plants	232
N(>0)	521
