# Supplementary material for: Early Domestication History of Asian Rice Revealed by Mutations and Genome-Wide Analysis of Gene Genealogies
Source: Rice (N Y). 2022 Feb 15;15:11. doi: 10.1186/s12284-022-00556-6 (PMC8847465; doi:10.1186/s12284-022-00556-6)
Supplement: Supplementary file 8 — Additional file 8: Table S5. Genotypes at four Os loci across accessions. [file 12284_2022_556_MOESM8_ESM.pdf]

Additional file 8

Supplemental Table 5. Genotypes at four *Os* loci among accessions.

Variety/landrace	*OsPGMp*	*OsAPS1*	*OsSSY3*	*OsCRX2*
**A jiao jiao jei**	*PGMp2_c*	*APS1_d*	*SSY3_b*	
**Aimazagu**	*PGMp2_a*	*APS1_a*	*SSY3_b*	*CKX2_b*
**Anjiaobaigu**	*PGMp2_d*	*APS1_a*	*SSY3_b*	*CKX2_c*
**Banjiemu**		*APS1_d*	*SSY3_a*	*CKX2_d*
**Baxiludao**	*PGMp2_c*	*APS1_b*	*SSY3_b*	*CKX2_a'*
**Bendihanggu_1**	*PGMp2_a*	*APS1_c*	*SSY3_b*	*CKX2_a*
**Bendihanggu_2**	*PGMp2_a*	*APS1_c*	*SSY3_b*	*CKX2_a*
**Bendiludao**	*PGMp2_a*	*APS1_c*	*SSY3_b*	
**Bendiruangu**	*PGMp2_d*	*APS1_a*	*SSY3_b*	
**Boshange**	*PGMp2_a*	*APS1_a*	*SSY3_b*	
**Boxuan01**	*PGMp2_a*	*APS1_a*	*SSY3_b*	
**Changbainuo**	*PGMp2_a*	*APS1_b'*	*SSY3_b'*	
**Changmaogu_1**		*APS1_d*	*SSY3_b*	*CKX2_a*
**Changmaogu_2**	*PGMp2_c*	*APS1_d*	*SSY3_a*	*CKX2_d*
**Changruanmi**	*PGMp2_b*	*APS1_b'*	*SSY3_b*	
**Chudao01**		*APS1_d*	*SSY3_a*	*CKX2_b*
**Chuhui16**	*PGMp2_c*	*APS1_d*	*SSY3_a*	*CKX2_b*
**Chujin26**	*PGMp2_c*	*APS1_d*	*SSY3_b*	*CKX2_b*
**Chujin27**		*APS1_d*	*SSY3_a*	*CKX2_b*
**Chujin28**	*PGMp2_c*	*APS1_d*	*SSY3_b*	*CKX2_b*
**Dabaigu**	*PGMp2_a*	*APS1_a*	*SSY3_b*	
**Dagu**	*PGMp2_e*		*SSY3_c*	*CKX2_a*
**Daheigu**	*PGMp2_d*	*APS1_a*	*SSY3_b*	*CKX2_c*
**Dahonggu**	*PGMp2_a*	*APS1_a*	*SSY3_b*	
**Dahonggu(red)**	*PGMp2_d*	*APS1_a*	*SSY3_b*	*CKX2_c*
**Dahonggu_2**	*PGMp2_d*	*APS1_c*	*SSY3_b*	*CKX2_e*
**Dahongjiaogu**	*PGMp2_a*	*APS1_b*	*SSY3_d*	*CKX2_b*
**Dahongnuo**	*PGMp2_c*	*APS1_d*	*SSY3_b*	
**Dahongruan**			*SSY3_c*	*CKX2_a*
**Dakeligu**	*PGMp2_a*	*APS1_b'*	*SSY3_b*	
**Denjian**	*PGMp2_d*	*APS1_c*	*SSY3_b*	
**Digu**	*PGMp2_c*	*APS1_d*	*SSY3_b*	*CKX2_a*
**Dinggeng**	*PGMp2_a*	*APS1_a*	*SSY3_b*	*CKX2_c*
**Donggu**	*PGMp2_a*		*SSY3_b*	*CKX2_b*
**Duoyenuo**	*PGMp2_b*	*APS1_b'*	*SSY3_b*	
**Ebinnong**		*APS1_d*		
**Eyongxiu**	*PGMp2_d*	*APS1_c*	*SSY3_b*	*CKX2_a*
**Ezaling**		*APS1_d*	*SSY3_b*	*CKX2_a*
**Fagunuo**	*PGMp2_a*	*APS1_b'&d*	*SSY3_a*	
**Fanglaobai**	*PGMp2_d*	*APS1_c*	*SSY3_b*	
**Fengdao17**	*PGMp2_c*	*APS1_d*	*SSY3_a*	*CKX2_b*
**Fengdao21**	*PGMp2_c*	*APS1_d*	*SSY3_a*	*CKX2_a*
**Fengdao23**		*APS1_d*	*SSY3_a*	*CKX2_a*
**Fengdao26**		*APS1_d*	*SSY3_a*	*CKX2_b*
**Fenggu01**	*PGMp2_d*	*APS1_a*	*SSY3_b*	*CKX2_e*
**Gaduogu**		*APS1_d*	*SSY3_b*	*CKX2_a*
**Gaoshanghong**	*PGMp2_a*		*SSY3_b*	*CKX2_a*
**Guque**	*PGMp2_c*	*APS1_d*	*SSY3_b*	
**Handizaogu**		*APS1_d*	*SSY3_b*	*CKX2_a*
**Hanxiangnuo**	*PGMp2_b*	*APS1_b'*	*SSY3_b'*	
**Haobixiangnong**	*PGMp2_d*	*APS1_a*	*SSY3_b*	
**Haogaodou(red)**	*PGMp2_b*	*APS1_b'*	*SSY3_b*	
**Haogaodou(red)**	*PGMp2_d*	*APS1_c*	*SSY3_c*	*CKX2_a*
**Haojiehai**	*PGMp2_a*	*APS1_b'*	*SSY3_b*	
**Haokandou(yellow)**	*PGMp2_d*	*APS1_d*	*SSY3_a*	
**Haomuhao**	*PGMp2_d*	*APS1_a*	*SSY3_b*	
**Haonuolong**	*PGMp2_b*	*APS1_b'*	*SSY3_b*	
**Haopai**	*PGMp2_b*	*APS1_b'*	*SSY3_b*	
**Haopie**		*APS1_a*	*SSY3_b*	*CKX2_b*
**Heidao**		*APS1_d*	*SSY3_a*	
**Heidianban**	*PGMp2_c*	*APS1_d*	*SSY3_b*	
**Heigu**	*PGMp2_a*	*APS1_b*	*SSY3_b*	
**Heigu**		*APS1_c*	*SSY3_c*	*CKX2_a*
**Heijieba**	*PGMp2_d*	*APS1_c*	*SSY3_b*	*CKX2_a*
**Heinuo01**	*PGMp2_c*	*APS1_d*		*CKX2_b*
**Heinuomi**	*PGMp2_a*	*APS1_d*	*SSY3_c*	
**Heizinuo**	*PGMp2_d*	*APS1_c*	*SSY3_b*	*CKX2_a*
**Hexi22-2**	*PGMp2_c*	*APS1_d*	*SSY3_b*	*CKX2_b*
**Hexi25**	*PGMp2_e*	*APS1_d*	*SSY3_a*	*CKX2_d*
**Hexi46**	*PGMp2_c*	*APS1_d*	*SSY3_a*	*CKX2_d*
**Hong20**	*PGMp2_a*	*APS1_a*	*SSY3_b*	*CKX2_b*
**Hongdianban**	*PGMp2_c*	*APS1_d*	*SSY3_b*	
**Hongerkuaigu**	*PGMp2_a*	*APS1_a*	*SSY3_b*	*CKX2_b*
**Honggengxi**	*PGMp2_a*	*APS1_a*	*SSY3_b*	*CKX2_b*
**Honggu**	*PGMp2_a*	*APS1_d*	*SSY3_b*	
**Honggu**	*PGMp2_a*	*APS1_a*	*SSY3_b*	
**Honggu**	*PGMp2_a*	*APS1_a*	*SSY3_b*	*CKX2_c*
**Hongguyangjie**	*PGMp2_a*	*APS1_a*	*SSY3_d*	*CKX2_b*
**Honghexi**	*PGMp2_a*	*APS1_b*	*SSY3_b*	*CKX2_c*
**Hongjiaogu**	*PGMp2_a*	*APS1_b*	*SSY3_d*	*CKX2_b*
**Hongnuogu**	*PGMp2_a*	*APS1_a*	*SSY3_b*	
**Hongnuomi**	*PGMp2_c*	*APS1_a*	*SSY3_a*	*CKX2_b*
**Hongruanmi(yellow)**	*PGMp2_a*	*APS1_a*	*SSY3_b*	
**Hongxiang01**	*PGMp2_a*	*APS1_d*	*SSY3_a*	*CKX2_b*
**Hongxinnuo**	*PGMp2_a*	*APS1_a*	*SSY3_b*	
**Huangnuogu**	*PGMp2_a*	*APS1_b'*	*SSY3_b*	
**Huigu**	*PGMp2_c*		*SSY3_a*	*CKX2_d*
**Hunagruanmi**	*PGMp2_c*	*APS1_d*	*SSY3_a*	
**Huohe**	*PGMp2_a*	*APS1_d*	*SSY3_b*	
**Huowu**	*PGMp2_a*	*APS1_a*	*SSY3_b*	
**Jieba**	*PGMp2_c*	*APS1_c*	*SSY3_b*	
**Jiegunuo**	*PGMp2_d*	*APS1_d*	*SSY3_b*	
**Jinguoyin**	*PGMp2_d*	*APS1_a*	*SSY3_b*	*CKX2_b*
**Jixuegu**	*PGMp2_d*	*APS1_a*	*SSY3_b*	
**Jugubai**	*PGMp2_a*	*APS1_a*	*SSY3_b*	

**Kaolalong**	*PGMp2_e*	*APS1_d*	*SSY3_c*	*CKX2_a*
**Kaopie**	*PGMp2_a*	*APS1_a*	*SSY3_b*	
**Kaopie**	*PGMp2_a*	*APS1_a*	*SSY3_b*	*CKX2_b*
**Kendao09**		*APS1_d*	*SSY3_a*	
**Kendao10**		*APS1_d*	*SSY3_a*	
**Kendao12a**		*APS1_d*	*SSY3_a*	
**Kendao12b**			*SSY3_a*	
**Kendao16**			*SSY3_a*	
**Kendao25**			*SSY3_a*	
**Kenjiandao3**		*APS1_d*	*SSY3_a*	
**Kenjiandao6**			*SSY3_a*	
**kongyu131_1**		*APS1_d*	*SSY3_a*	
**kongyu131_2**			*SSY3_a*	
**Landinuo**	*PGMp2_a*	*APS1_d*	*SSY3_c*	
**Laodagu**	*PGMp2_c*		*SSY3_b*	*CKX2_b*
**Laolaihong**	*PGMp2_e*	*APS1_d*	*SSY3_c*	*CKX2_a*
**Laolaihong**		*APS1_a&d*	*SSY3_b*	*a&b*
**Laotiangu**	*PGMp2_a*	*APS1_a*	*SSY3_b*	
**Lengshuidao(yellow)**	*PGMp2_a*	*APS1_a*	*SSY3_b*	
**Lengshuidiao**	*PGMp2_e*		*SSY3_c*	*CKX2_a*
**Lianjin09**	*PGMp2_c*	*APS1_d*	*SSY3_c*	*CKX2_b*
**Liaoxing01**		*APS1_d*	*SSY3_a*	
**Liaoxing20**			*SSY3_a*	
**Longhua04426**			*SSY3_a*	
**Longjiao04-1963**			*SSY3_a*	
**Longjiao04-2411**			*SSY3_a*	
**Longjin01**		*APS1_d*	*SSY3_a*	
**Longjin04**	*PGMp2_a*	*APS1_d*	*SSY3_b*	*CKX2_b*
**Longjin20**			*SSY3_a*	
**Longjin21**		*APS1_d*	*SSY3_a*	
**Longjin24**			*SSY3_a*	
**Longjin25**		*APS1_d*	*SSY3_a*	
**Longjin26**		*APS1_d*	*SSY3_a*	
**Longjin27**		*APS1_d*	*SSY3_a*	
**Longjin29**			*SSY3_a*	
**Longjin30**			*SSY3_b*	
**Longjin31**		*APS1_d*	*SSY3_a*	
**Longjin36**			*SSY3_a*	
**Longjin39**			*SSY3_a*	
**Longjin40**		*APS1_d*	*SSY3_a*	
**Longjin41**		*APS1_d*	*SSY3_a*	
**Longjinxiang01**		*APS1_d*	*SSY3_a*	
**Longsheng04042**		*APS1_d*	*SSY3_c*	
**Lufenggu**	*PGMp2_a*	*APS1_a*	*SSY3_b*	*CKX2_b*
**Maheinuo**	*PGMp2_b*	*APS1_b'*	*SSY3_b*	
**Maimanggu**	*PGMp2_c*	*APS1_c*	*SSY3_b*	*CKX2_a*
**Malugu**	*PGMp2_a*	*APS1_a*	*SSY3_b*	
**Manbieheigannuo**	*PGMp2_a*	*APS1_b'*	*SSY3_b*	
**Manpangxichanggu**	*PGMp2_b*	*APS1_b'*	*SSY3_b*	
**ManpixiangHongnuomi**		*APS1_a*	*SSY3_b*	*CKX2_c*
**Maxiangu**	*PGMp2_a*	*APS1_a*	*SSY3_b*	*CKX2_c*
**Mazagu**	*PGMp2_a*	*APS1_a*	*SSY3_b*	*CKX2_c*
**Menglaguo**	*PGMp2_a*	*APS1_b'*	*SSY3_a*	
**Mengsonggu**	*PGMp2_b*	*APS1_a*	*SSY3_b*	*CKX2_c*
**Mengwanggu**	*PGMp2_c*	*APS1_d*	*SSY3_b*	
**Mengxinggu**	*PGMp2_a*	*APS1_b'*	*SSY3_b*	
**Mianmusikan**	*PGMp2_a*	*APS1_a*	*SSY3_b*	*CKX2_b*
**Miaojiachangmaogu**	*PGMp2_b*	*APS1_a*	*SSY3_b*	
**Nanjin49**	*PGMp2_c*	*APS1_d*	*SSY3_a*	*CKX2_b*
**Nanwenqieba**	*PGMp2_d*	*APS1_c*	*SSY3_b*	
**Nongzi11**	*PGMp2_b*	*APS1_b'*	*SSY3_b*	*CKX2_c*
**Nuogu**	*PGMp2_a*	*APS1_b'*	*SSY3_b'*	
**Ouniaoguo**	*PGMp2_a*	*APS1_b'*	*SSY3_b*	
**Piaolianggu**	*PGMp2_c*	*APS1_d*	*SSY3_b*	
**Qitougu**		*APS1_a*	*SSY3_b*	*CKX2_b*
**Ruanmazinuo**	*PGMp2_b*	*APS1_b'*	*SSY3_b*	
**Ruiligu**	*PGMp2_a*	*APS1_a*	*SSY3_b*	
**Sanbaizi**	*PGMp2_a*	*APS1_b'*	*SSY3_b*	
**Shangmengu**	*PGMp2_c*	*APS1_d*	*SSY3_b*	
**Shendao47**			*SSY3_a*	
**Shiyuenuo**	*PGMp2_a*	*APS1_a*	*SSY3_b*	*CKX2_c*
**Shuijinghangu**	*PGMp2_a*	*APS1_a*	*SSY3_b*	
**Song820**		*APS1_d*	*SSY3_a*	
**Songjin03**			*SSY3_b*	
**Songjin05**	*PGMp2_c*	*APS1_d*	*SSY3_a*	*CKX2_b*
**Songjin06**	*PGMp2_c*	*APS1_d*	*SSY3_a*	*CKX2_a*
**Songjin07**	*PGMp2_c*	*APS1_d*	*SSY3_a*	*CKX2_a*
**Songjin08**	*PGMp2_c*	*APS1_d*	*SSY3_a*	*CKX2_a*
**Songjin09**	*PGMp2_c*	*APS1_d*	*SSY3_a*	*CKX2_a*
**Songjin10**			*SSY3_a*	
**Songjin11**		*APS1_a*	*SSY3_b*	
**Songjin12**		*APS1_a*	*SSY3_b*	
**Songjin13**			*SSY3_a*	
**Songjin15**		*APS1_d*	*SSY3_a*	
**Songjin16**			*SSY3_b*	
**Songjin17**			*SSY3_a*	
**Songjin18**		*APS1_d*	*SSY3_a*	
**Songjin19**			*SSY3_a*	
**Songnian01**	*PGMp2_c*	*APS1_d*	*SSY3_a*	

**Suijin04**	*PGMp2_c*	*APS1_d*	*SSY3_b*	
**Suijin08**	*PGMp2_c*	*APS1_d*	*SSY3_a*	*CKX2_a*
**Suijin10**	*PGMp2_c*	*APS1_d*	*SSY3_a*	*CKX2_b*
**Teheinuomi**	*PGMp2_d*	*APS1_d*	*SSY3_b*	
**Tianza**	*PGMp2_a*	*APS1_a*	*SSY3_b*	
**Tk57**	*PGMp2_c*	*APS1_d*	*SSY3_a*	*CKX2_a'*
**Tk58**	*PGMp2_c*	*APS1_d*	*SSY3_a*	*CKX2_a*
**Tk77**	*PGMp2_d*	*APS1_d*	*SSY3_a*	*CKX2_b*
**Tk78**	*PGMp2_c*	*APS1_d*	*SSY3_a*	*CKX2_b*
**Wangyou9801**	*PGMp2_a*	*APS1_a*	*SSY3_b*	*CKX2_e*
**Wuyoudao01**	*PGMp2_c*	*APS1_d*	*SSY3_a*	*CKX2_a*
**Wuyoudao04**	*PGMp2_a*	*APS1_d*	*SSY3_a*	*CKX2_b*
**Wuzuigu**		*APS1_d*	*SSY3_c*	*CKX2_a*
**Xianggu**	*PGMp2_a*	*APS1_a*	*SSY3_b*	
**Xiangnuo**	*PGMp2_c*	*APS1_d*	*SSY3_b*	
**Xiangnuo_1**	*PGMp2_a*	*APS1_b'*	*SSY3_b*	
**Xiangnuo_2**	*PGMp2_a*	*APS1_a*	*SSY3_b*	
**Xiangnuogu**	*PGMp2_a*	*APS1_b'*	*SSY3_b*	*CKX2_c*
**Xiangsigu**		*APS1_a*	*SSY3_b*	*CKX2_c*
**Xiangzinuo**	*PGMp2_b*	*APS1_b'*	*SSY3_b*	
**Xiaoheigu**	*PGMp2_e*	*APS1_d*	*SSY3_b*	*CKX2_a*
**Xiaohuagu**	*PGMp2_d*	*APS1_a*	*SSY3_b*	*CKX2_b*
**Xiaohuanggu**	*PGMp2_d*	*APS1_a*	*SSY3_b*	
**Xiaoxigu**	*PGMp2_d*	*APS1_a*	*SSY3_b*	
**Xihonggu**		*APS1_a*	*SSY3_b*	*CKX2_b*
**Xihonggu(possible)**	*PGMp2_a*	*APS1_a*	*SSY3_b*	
**Xihongruan**	*PGMp2_a*		*SSY3_b*	*CKX2_b*
**Yaojiahonggu**	*PGMp2_a*	*APS1_a*	*SSY3_b*	
**Yidunban**	*PGMp2_d*	*APS1_a*	*SSY3_b*	*CKX2_d*
**Yingkagu**	*PGMp2_a*	*APS1_d*	*SSY3_b*	
**Yirongnuan**	*PGMp2_a*	*APS1_c*	*SSY3_b*	
**Youjin03**	*PGMp2_c*		*SSY3_a*	*CKX2_a*
**Youjin15**			*SSY3_a*	*CKX2_b*
**Youjin20**	*PGMp2_c*	*APS1_d*	*SSY3_a*	*CKX2_a*
**Youmangzigu**	*PGMp2_e*	*APS1_d*	*SSY3_b*	*CKX2_a*
**Yunhui290**	*PGMp2_a*	*APS1_a*	*SSY3_b*	
**Yunjin20**		*APS1_d*	*SSY3_b*	*CKX2_a*
**Yunjin29**	*PGMp2_c*	*APS1_d*	*SSY3_a*	*CKX2_a*
**Yunjin41**	*PGMp2_c*	*APS1_d*	*SSY3_a*	*CKX2_a*
**Yunnong04**		*APS1_b'*	*SSY3_b*	*CKX2_d*
**Zaohuagumi**	*PGMp2_a*	*APS1_a*	*SSY3_b*	*CKX2_b*
**Zhangbeng**	*PGMp2_c*	*APS1_d*	*SSY3_b*	*CKX2_a*
**Zhengdao11**		*APS1_d*	*SSY3_c*	*CKX2_b*
**Zhuhongdan**	*PGMp2_a*	*APS1_a*	*SSY3_b*	
**Zhuhongdan**	*PGMp2_a*	*APS1_a*	*SSY3_b*	
**Zhuhongnian**	*PGMp2_a*	*APS1_a*	*SSY3_b*	*CKX2_c*
**Zigenuo**	*PGMp2_b*	*APS1_a*	*SSY3_b*	
**Zigu**	*PGMp2_b*	*APS1_a*	*SSY3_b*	
**Zinuo**		*APS1_d*	*SSY3_c*	*CKX2_a*
**Zinuo**	*PGMp2_a*	*APS1_c*	*SSY3_b*	*CKX2_b*
**Zinuomi**	*PGMp2_a*	*APS1_c*	*SSY3_b*	
**Zinuomi**	*PGMp2_c*	*APS1_d*	*SSY3_b*	*CKX2_c*
**Zulugu**		*APS1_b*	*SSY3_b*	*CKX2_b*
**Zulugu_2**	*PGMp2_a*	*APS1_a*	*SSY3_b*	*CKX2_b*
**Subtotal**	**169**	**206**	**236**	**110**
