# Supplementary material for: Early Domestication History of Asian Rice Revealed by Mutations and Genome-Wide Analysis of Gene Genealogies
Source: Rice (N Y). 2022 Feb 15;15:11. doi: 10.1186/s12284-022-00556-6 (PMC8847465; doi:10.1186/s12284-022-00556-6)
Supplement: Supplementary file 9 — Additional file 9: Table S6. Comparisons of the model genomes (upper panel) with alleles of OsSSY3 (lower panel) in rice cultivars. [file 12284_2022_556_MOESM9_ESM.pdf]

Additional file 9

Supplemental Table 6. Comparisons of the model genomes (upper panel) with alleles of *OsSSY3* (lower panel) in rice cultivars.

**Plant**	***SSY3***	**3481**	**3559**	**3779**	**3900**	**3922**	**Frequency**
Oryza nivara	*OnSSY3*	G	A	A	C	G	
O. sativa Indica	*OsSSY3-Shuhui*	G	A	A	A	A	
O. rufipogon	*OrSSY3*	A	A	T	C	A	
O. sativa Japonica	*OsSSY3-Nipp*	G	T	A	C	A	
Other 236 cultivars of *O. sativa*	*OsSSY3_a*	G	T	A	C	A	0.32
	OsSSY3_b	G	A	A	C	A	0.59
	OsSSY3_b'	G	A	A	A	A	0.01
	OsSSY3_c	A	A	T	C	A	0.06
	OsSSY3_d	G	A	A	C	G	0.01

The genomic region here covers part of the third exon numbered from nucleotide 3450 to 4326, with the first nucleotide of the first exon as 1.
