# Supplementary material for: Early Domestication History of Asian Rice Revealed by Mutations and Genome-Wide Analysis of Gene Genealogies
Source: Rice (N Y). 2022 Feb 15;15:11. doi: 10.1186/s12284-022-00556-6 (PMC8847465; doi:10.1186/s12284-022-00556-6)
Supplement: Supplementary file 10 — Additional file 10: Table S7. Comparisons of model genomes (upper panel) with alleles of OsPGMp (lower panel) surveyed in rice materials. [file 12284_2022_556_MOESM10_ESM.pdf]

Additional file 10

Supplemental Table 7. Comparisons of model genomes (upper panel) with alleles of *OsPGMp* (lower panel) surveyed in rice materials.

Plant	*PGMp*	6130	6159	6170	6183	6192	6210	6213	6247	6271	6287	6395	6480	6491	6498	6499	Frequency
O. nivara	*OnPGMp*	-	A	T	C	A	C	C	G	G	A	A	GCTC	C	T	CT	
O. sativa	*OsPGMp*-Shuhui	-	A	T	C	A	C	C	G	T	A	A	GCTC	C	T	CT	
O. rufipogon	*OrPGMp*	AA	-	C	C	T	A	T	A	G	T	T	-	A	C	-	
O. sativa	*OsPGMp*-Nipp	-	A	T	C	A	C	C	G	G	A	A	GCTC	-	T	-	
Other 169 cultivars/ landraces of *O. sativa*	*OsPGMp_a*	-	A	T	C	A	C	C	G	G	A	A	GCTC	C	T	CT	0.44
	OsPGMp_b	-	A	T	C	A	C	C	G	T	A	A	GCTC	C	T	CT	0.09
	OsPGMp_c	-	A	T	C	A	C	C	G	G	A	A	GCTC	C	T	-	0.28
	OsPGMp_d	-	A	T	T	A	C	C	G	G	A	A	GCTC	C	T	CT	0.15
	OsPGMp_e	-	A	T	C	A	C	C	G	G	A	A	GCTC	C	T	CTCT	0.04

The nucleotide numbering starts from 1 at the first nucleotide of the first exon. The comparison spans 6032-6512 nucleotides between the 17th exon and the 19th intron.
