# Supplementary material for: Early Domestication History of Asian Rice Revealed by Mutations and Genome-Wide Analysis of Gene Genealogies
Source: Rice (N Y). 2022 Feb 15;15:11. doi: 10.1186/s12284-022-00556-6 (PMC8847465; doi:10.1186/s12284-022-00556-6)
Supplement: Supplementary file 11 — Additional file 11: Table S8. Comparisons of model genomes (upper panel) with alleles of OsCKX2 (lower panel) surveyed in rice. [file 12284_2022_556_MOESM11_ESM.pdf]

Additional file 11

Supplemental Table 8. Comparisons of model genomes (upper panel) with alleles of *OsCKX2* (lower panel) surveyed in rice.

Plant	***CKX2***	3650	3949	3950	4004	4016	4029	4070	4114	4209	4221	4255	4281	4302	4369	4462	Frequency
O. nivara	*OnCKX2*	T	T	A	T	T	G	T	A	C	C	C	A	C	G	A	
O. sativa	*OsCKX2*-9311	T	A	C	T	T	G	T	A	C	C	C	-	-	G	G	
O. rufipogon	*OrCKX2*	T	A	A	T	T	G	T	A	T	C	C	G	C	G	G	
O. sativa	*OsCKX2*-Nipp	C	A	A	T	C	G	T	G	C	C	C	G	T	C	G	
Other 110 cultivars/ landraces of *O. sativa*	*OsCKX2_a*	C	A	A	T	C	G	T	G	C	C	C	G	T	C	G	0.41
	OsCKX2_b	T	A	C	T	T	G	T	A	C	C	C	-	-	G	G	0.40
	OsCKX2_c	T	T	A	T	T	A	T	A	C	C	C	-	-	G	G	0.14
	OsCKX2_d	T	A	A	T	T	G	T	A	C	C	T	-	-	G	G	0.03
	OsCKX2_d'	T	A	A	C	T	G	T	A	C	T	C	G	C	G	G	0.02

The nucleotide numbering starts from 1 at the first nucleotide of the first exon. The comparison covers nucleotides numbered 3316-4579 between the 2nd intron and the 4th exon.
