# Supplementary material for: Early Domestication History of Asian Rice Revealed by Mutations and Genome-Wide Analysis of Gene Genealogies
Source: Rice (N Y). 2022 Feb 15;15:11. doi: 10.1186/s12284-022-00556-6 (PMC8847465; doi:10.1186/s12284-022-00556-6)
Supplement: Supplementary file 12 — Additional file 12: Table S9. Random samples on identified polymorphisms from NCBI. [file 12284_2022_556_MOESM12_ESM.pdf]

Additional file 12

Supplemental Table 9. Random samples on identified polymorphisms from NCBI.

Chromosome	Locus	Region	Polymorphism[a]	Subgroup	Frequency	Variety	Note
Chr01	*ME*	Exon 2	G	*Indica*	30/30	RPBio226,Zhenshan97,Minghui63, 9311, Isolates A9-A12 (EU229144-47),B1-B8 & B10-B12(EU229148-59),C1-C8(EU229160-67),E10 (EU229193)	
			Later A (from G)	*Japonica*	33/34	Nipponbare, Kitaake, Isolates A3-A5(EU229138-40),C9-C11(EU229168-71), D1-D8 (EU229172-79), D10(EU229181), D12(EU229183), E1-E3(EU229184-6), E5-E9(EU229188-92),E11(EU229194), F1-F3(EU229196-8), F7(EU229202), F9-F11(EU229204-6)	E3-Indica type
				Indica	1/34	E3(EU229186)	
Chr01	*DFR*	5' region	**Early idl**	*Indica*	6/6	Shuhui498,RPBio226,Zhenshan97,Minghui63,9311,Teqing(U70541)	
				Japonica	3/3	Nipponbare, Kitaake,Murasaki-ine(AB003495)	
			**Early A (from G)**	*Indica*	6/6	Shuhui498,RPBio226,Zhenshan97,Minghui63,9311	
				Japonica	3/3	Nipponbare, Kitaake,Murasaki-ine(AB003495)	
		Exon 1	C	*Indica*	16/18	9311, Heishuai (MK636607), Shuhui498,Minghui63, Zhenshan97, RP Bio-226, IR64_OrC_5-6(MW310657-8), IR64_OrC_9-10 (MW310655-6), IR64_OrC_62(MW310653), CYB_OrB_56(MW310652), CYB_OrB_3(MW310651),Rd_C6-9(MW310650), Rd_Cybonnet(MW310645),Rd_IR64(MW310644)	
				Japonica	2/18	Murasaki-ine(AB003495),Koshihikari(AB010744)	
			Later A (from C)	*Japonica*	3/5	Nipponbare,Kitaake,Toride-1(AB010745),Murasaki-ine(AB003495),Koshihikari(AB010744)	Murasaki-ine &Koshihikari –I type
		Exon 2	A	*Indica*	15/15	9311, Shuhui498,RP-Bio-226, Zhenshan97, Minghui63, Heishuai (MK636607), IR64_OrC_5-6 (MW310657-8), IR64_OrB_9-10(MW310655-6), CYB_OrC_62(MW310653), CYB_OrB_ 56(MW310652), Rd_IR64(MW310644), Teqing (U70541), Purpleputtu (Y07956)	
			Later G (from A)	*Japonica*	3/3	Nipponbare, Kitaake,Murasaki-ine(AB003495)	
Chr03	*GL3.2*	5' region	Later idl1	*Indica*	3/5	9311, Zhenshan97, RP Bio226	
			Later idl2	*Indica*	2/5	Shuhui498, Minghui63,	
			Later idl3	*Japonica*	2/2	Nipponbare, Kitaake	
			**Early idl(T)**	*Indica*	5/5	9311, Zhenshan97, RP Bio226, Shuhui498, Minghui63	
				Japonica	2/2	Nipponbare, Kitaake	
			**Early idl(C)**	*Indica*	2/5	*9311, Zhenshan97, RP Bio226,* Shuhui498, Minghui63	Varieties in italic not having the idl.
				Japonica	2/2	Nipponbare, Kitaake	
			**Early idl(CT)**	*Indica*	2/5	*9311, Zhenshan97, RP Bio226,* Shuhui498, Minghui63	Varieties in italic not having the idl.
				Japonica	2/2	Nipponbare, Kitaake	
Chr04	*MYB15*	Exon 3	**Early idl(4aa)**	*Indica*	6/6	9311, Zhenshan97, RP Bio226, Shuhui498, Minghui63, Kasalath	
				Japonica	3/3	Nipponbare, Kitaake, Arborio(Y11414)	
			**Early idl(1aa)**	*Indica*	6/6	9311, Zhenshan97, RP Bio226, Shuhui498, Minghui63, Kasalath	
				Japonica	3/3	Nipponbare, Kitaake, Arborio(Y11414)	
Chr06	*Hd3a*	5' region	**Early idl**	*Indica*	15/15	Shuhui498, RPBio226, Zhenshan97, Minghui63, 9311, Shuusouchu(AP011451), GuangLuAi4(KR611195), AiChiaoHong(KM043289), ChinGaley(AB838405), Milyang23(AB838394), LocalBasmati(AB838383), ShweNangGyi(AB838365),RyouSuisanKoumai(AB838357),QiuZhaoZhong(AB564444),Mehr(AB838260)	
				Japonica	14/14	Nipponbare,Kitaake,KhauMacKho(AP011450),Taichung65(KR611196), Tima(AB838392), Jaguary(AB838387), Houmanshindenine(AB838344), Shinriki(AB838324), TaHungGu(AB838246), Phudugey(AB838269), Akage(AB838308), Oiran(AB838305), GaisenMochi(AB838296), Wateribune(AB838310)	
			**Early A (from T)**	*Indica*	16/16	Shuhui498,RPBio226,Zhenshan97,Minghui63,9311,Shuusouchu(AP011451), GuangLuAi4(KR611195), ChinGaley(AB838405), Milyang23(AB838394), LocalBasmati(AB838383), ShweNangGyi(AB838365), RyouSuisanKoumai(AB838357), QiuZhaoZhong(AB564444), Mehr(AB838260), AiChiaoHong(AB838275), Basmati370(AB426882), Kasalath(AP011452)	
				Japonica	15/15	Nipponbare, Kitaake, KhauMacKho(AP011450), Taichung65(KR611196), Tima(AB838392), Jaguary(AB838387), Houmanshindenine(AB838344), Shinriki(AB838324), TaHungGu(AB838246), Phudugey(AB838269), Akage(AB838308), Oiran(AB838305), GaisenMochi(AB838296), Wateribune(AB838310), Ginbouzu(AB838318)	
			**Early A (from G)**	*Indica*	16/16	Shuhui498, RPBio226, Zhenshan97, Minghui63, 9311, Shuusouchu(AP011451), GuangLuAi4(KR611195), AiChiaoHong(KM043289), ChinGaley(AB838405), Milyang23(AB838394), LocalBasmati(AB838383), ShweNangGyi(AB838365), RyouSuisanKoumai(AB838357), QiuZhaoZhong(AB564444), Mehr(AB838260), SeratoesHari(AB838255)	
				Japonica	15/15	Nipponbare, Kitaake, KhauMacKho(AP011450), Taichung65(KR611196), Tima(AB838392), Jaguary(AB838387), Houmanshindenine(AB838344), Shinriki(AB838324), TaHungGu(AB838246), Phudugey(AB838269), Akage(AB838308), Oiran(AB838305), GaisenMochi(AB838296), Wateribune(AB838310), Ginbouzu(AB838318)	
			**Early C (from T)**	*Indica*	15/15	Shuhui498, RPBio226, Zhenshan97, Minghui63, 9311, Shuusouchu(AP011451), GuangLuAi4(KR611195), ChinGaley(AB838405), Milyang23(AB838394), LocalBasmati(AB838383), ShweNangGyi(AB838365), RyouSuisanKoumai(AB838357), QiuZhaoZhong(AB564444), Mehr(AB838260), SeratoesHari(AB838255), Kasalath(AP011452)	
				Japonica	15/15	Nipponbare, Kitaake, KhauMacKho(AP011450), Taichung65(KR611196), Tima(AB838392), Jaguary(AB838387), Houmanshindenine(AB838344), Shinriki(AB838324), TaHungGu(AB838246), Phudugey(AB838269), Akage(AB838308), Oiran(AB838305), GaisenMochi(AB838296), Wateribune(AB838310), Ginbouzu(AB838318)	
Chr08	*RAE2*	Exon 2	**Early idl**	*Indica*	7/7	Shuhui498, RPBio226, Zhenshan97, Minghui63, 9311, IRGC117889, GuangLuAi4(CT835915)	
				Japonica	2/3	Nipponbare, Kitaake, NSFTV223(tropical J)	Kitaake-recombinant

[a] Early mutations are in bold.
