# Supplementary material for: Early Domestication History of Asian Rice Revealed by Mutations and Genome-Wide Analysis of Gene Genealogies
Source: Rice (N Y). 2022 Feb 15;15:11. doi: 10.1186/s12284-022-00556-6 (PMC8847465; doi:10.1186/s12284-022-00556-6)

Additional file 13

Supplemental Table 10. Population-level examinations of mixed contributions from *Or* and *On* to *OsHd3a* based on NCBI data.

Locus	Region	Sites	Population	Frequency	Variety	Note
Hd3a	5' region	C from *Or* and A from *On*	*Indica*	28/28	Shuhui498, Zhenshan97, Minghui63, 9311, GuangLuAi4 (KR611195), Shuusouchu (AP011451), ChinGalay (AB838405), PadiKuning (AB838400), Hakphaynhay (AB838397), NeangMenh (AB838395), Milyang23 (AB838394), LocalBasmati (AB838383), ShweNangGyi (AB838365), RyouSuisanKoumai (AB838357), QiuZhaoZhong (AB564444), AichiaoHong (AB838275) ,KhaoDawkMail103 (AB838265), NaraAswina (AB838263), KaoGaew (AB838260), Mehr (AB838260), BR8 (AB838259), SeratoesHari (AB838255), GIE57 (AB838252), TRS4 (AB838250), DA11 (AB838247), TKM6 (AB838245), BeiKhe (KM043288), ChhoteDhan (KM043301)	See attached figure below for detail
			Japonica	16/16	Nipponbare, Kitaake, KhauMacKho (AP011450), Taichung65 (KR611196), Tima (AB838392), Jaguary (AB838387), Houmanshindenine (AB838344), Shinriki (AB838324), Phudugey (AB838269), Akage (AB838308), Oiran (AB838305), GaisenMochi (AB838296), Wateribune (AB838310), Ginbouzu (AB838318), Tropical J-KotobukiMochi (KM043291), Akamai (AB838312)	

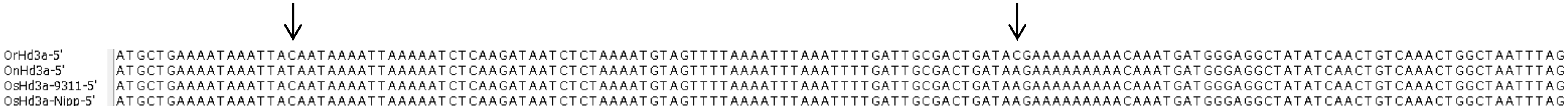

Supplement: Supplementary file 13 — Additional file 13: Table S10. Population-level examinations of mixed contributions of Or and On to OsHd3a based on NCBI data. [file 12284_2022_556_MOESM13_ESM.pdf]
