# Supplementary material for: Early Domestication History of Asian Rice Revealed by Mutations and Genome-Wide Analysis of Gene Genealogies
Source: Rice (N Y). 2022 Feb 15;15:11. doi: 10.1186/s12284-022-00556-6 (PMC8847465; doi:10.1186/s12284-022-00556-6)
Supplement: Supplementary file 14 — Additional file 14: Fig. S2. Comparisons of predictions under different hypotheses. [file 12284_2022_556_MOESM14_ESM.pdf]

Additional file 14

Hypothesis A (Lu et al. this study) vs. Hypothesis B (Huang et al. 2012)

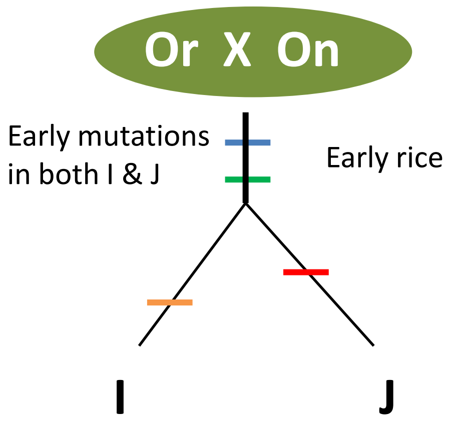

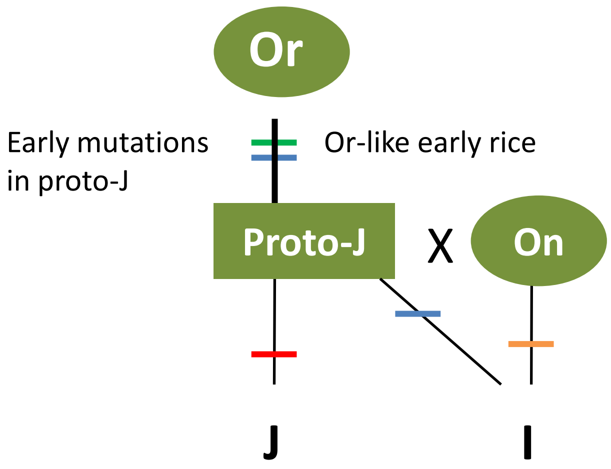

Predictions under the hypothesis A

(1) Shared early mutations in rice populations of I and J.

(2) Presence of both *On* and *Or* alleles in genomes of I and J.

(3) Recombinants of *On* and *Or* alleles in I and J.

Relevant Data

Predicted (1) is supported by fixed early mutations in different populations of japonica and indica (Table S9, Table S10).

Prediction (2) is supported by Type 2 and Type 3 genes (Table S3) in genomes of japonica (e.g., Nipponbare and Kitaake) and indica (e.g., 9311, Shuhui-498, and RP Bio-226).

Prediction (3) is supported by recombinant alleles shown for both indica and japonica (Fig. 4a).

Predictions under the hypothesis B

(1) Absence of *On* alleles in japonica genomes.

(2) No recombinant alleles in japonica varieties.

(3) A hybrid origin of indica (but not japonica) led to more genetic diversity accumulated in indica than in japonica.

Relevant Data

Prediction (1) is rejected as sampled 101 genes show that J genomes (e.g., those of Nipponbare and Kitaake) contain at least 4 loci of *On* alleles and 5 *On*-like genes (Table S1 and Table S11).

Prediction (2) is rejected, since a recombinant allele is seen at the locus *APS1* in japonica (Fig. 4a).

Prediction (3) is supported neither by the number of japonica- or indica-specific loci with positively selected mutations (12 vs. 10) cited in Table 2 and Table S2 nor by the total number of mutations (Table S2) specific to indica (96) or to japonica (110).

Supplemental Fig.2. Comparisons of predictions under different hypotheses. One commonly cited hypothesis on the origin of Asian rice (the hypothesis B here) is compared to the scenario proposed in this study, based on three predictions under each hypothesis. Relevant data are from evidence shown in this study. Colored bars represent mutations on different lineages for conceptual presentation only. Plants involved in the origin of rice are in green background, with On for *O. nivara*, Or for *O. rufipogon*, I for indica, and J for japonica.
