# Supplementary material for: Early Domestication History of Asian Rice Revealed by Mutations and Genome-Wide Analysis of Gene Genealogies
Source: Rice (N Y). 2022 Feb 15;15:11. doi: 10.1186/s12284-022-00556-6 (PMC8847465; doi:10.1186/s12284-022-00556-6)
Supplement: Supplementary file 15 — Additional file 15: Table S11. Genomic comparisons of Kitaake with reference genomes across 101 genes. [file 12284_2022_556_MOESM15_ESM.pdf]

Additional file 15

Supplemental Table 11. Genomic comparisons of Kitaake with referenced genomes across 101 genes.

Chromosome	MSU ID	Rice gene	Kitaake genome[a]		Comparisons with referenced genomes[b]		Graphic display[c] of early mutations	Graphic display[c] of later mutations
			5'	Coding	5'	Coding		
**1**	Os01g09320.1	***ME***	Kitaake	Kitaake	1 subs: Kitaake type	1 nons:Kitaake type	No dispute	No dispute
	Os01g10110.1	***CKX2***	Nipp	Nipp			No dispute	No dispute
	Os01g27490.1	***ANS1***	Nipp	Nipp			No dispute	No dispute
	Os01g44260.1	***DFR***	Nipp	Nipp			No dispute	No dispute
	Os01g54860.1	***NOG1***	Nipp	Nipp			No dispute	No dispute
	Os01g55870.1	***CM3***	Nipp	Nipp			No dispute	No dispute
	Os01g60190.1	***iPGAM1***	Nipp	Nipp			No dispute	No dispute
	Os01g62920.1	***qSH1***	Nipp	Nipp			No dispute	No dispute
	Os01g66100.1	***SD1***	Nipp	Nipp			No dispute	No dispute
**2**	Os02g32660.1	***SBE3***	Kitaake	9311	2 subs:Kitaake(G->T & G->A),3 SNPs same as 9311	9311	No dispute	No dispute
	Os02g41680.1	***ZB8***	Kitaake	Nipp	one idl:Kitaake type		No dispute	No dispute
	Os02g45810.2	***TTG1***	Nipp	Nipp			No dispute	No dispute
	Os02g46220.1	***SK2***	Kitaake	Nipp	one idl:Kitaake type		No dispute	No dispute
	Os02g52840.1	***FLS***	Nipp	Nipp			No dispute	No dispute
	Os02g49230.1	***DTH2***	Nipp	**Kitaake**		**disagrees with Nipp at T(nons)**	No dispute	One later nons(specific to J)is not in Kitaake.
**3**	Os03g11614.1	***MADS1***	Nipp	Nipp			No dispute	No dispute
	Os03g14990.1	***CS***	Kitaake	Kitaake	1 subs:Kitaake & 1(C)agrees with *Indica*	2 syn:One agrees with Or & one Kitaake	No dispute	One 5' later mut(C->G,specific to Nipp)and one coding (C->T,specific to Nipp)are not in Kitaake.
	Os03g15360.2	***LAR***	Kitaake	Nipp	3 muts:Kitaake,2 SNP:Nipp, 5SNPs/idl:9311	`	No dispute	No dispute
	Os03g21260.1	***Unknown***	Nipp	Nipp			No dispute	No dispute
	Os03g22120.2	***SUS4***	Nipp	Nipp			No dispute	No dispute
	Os03g27230.1	***DAHPS1***	Nipp	Nipp			No dispute	No dispute
	not listed	***GS3***	Nipp	Nipp			No dispute	No dispute
	Os03g29614.1	***OsMYB3***	Nipp	Nipp			No dispute	No dispute
	Os03g30420.1	***GL3.2***	Nipp	Nipp			No dispute	No dispute
	Os03g49880.1	***TB1***	Nipp	Nipp			No dispute	No dispute
	Os03g55389.1	***Hd6***	Nipp	Nipp			No dispute	No dispute
	Os03g57240.1	***Dst***	Kitaake	Nipp	1 subs:Kitaake, others as Nipp		No dispute	No dispute
	Os03g60509.1	***CHI***	Nipp	Nipp			No dispute	No dispute
	Os03g61120.1	***ASA1***	Nipp	Nipp			No dispute	No dispute
**4**	Os04g28280.2	***An-1***	Nipp	Kitaake		Kitaake=Guangluai4, Shuhui-like	No dispute	2 coding-idl disputes with Shuhui
	Os04g33740.1	***GIF1***	Kitaake	Nipp	1 idl:Kitaake, others as Nipp		No dispute	No dispute
	Os04g43680.1	***MYB15***	Nipp	Nipp			No dispute	No dispute
	Os04g43840.1	***An-2***	Nipp	Nipp			No dispute	No dispute
	Os04g47040.1	***Unknown***	Kitaake	Nipp	1 later subs of Nipp is not present		No dispute	One 5' later subs(G->A,specific to Nipp)is not in Kitaake.
	Os04g47059.1	***B2***	Nipp	Kitaake*		Nipp is a mutant, Plw differs from Kitaake in 1 syn (C->A). Kitaake was used as reference	No dispute	No dispute
	Os04g47080.1	***B1***	Nipp	Kitaake		9311,Nipponbare, Kitaake each has a different deletion in one of the last 2 exons.	No dispute	No dispute
	Os04g52540.1	***AGO2***	Nipp	Nipp			No dispute	No dispute
	Os04g56580.1	***IPK1***	Nipp	Nipp			No dispute	No dispute
	Os04g56700.1	***F3H***	Nipp	Nipp			No dispute	No dispute
	Os04g57530.1	***SH4***	Kitaake	Nipp	1 idl:Kitaake=9311, others as Nipp		No dispute	One 5' later idl(A string,specific to Nipp)is not in Kitaake.
	Os04g58110.1	***PK3***	Nipp	Nipp			No dispute	No dispute
	Os04g55560.1	***SHAT1***	Nipp	Nipp			No dispute	No dispute
**5**	Os05g06480.1	***Chalk5***	Nipp	Nipp			No dispute	No dispute
	Os05g06660.1	***GS5***	Nipp	Nipp			No dispute	No dispute
	Os05g09520.1	***qSW5***	Kitaake	Nipp	1 idl:Kitaake=9311, others same as Nipp		No dispute	One 5' later idl(T insertion,specific to Nipp)is not in Kitaake.
	Os05g10780.1	***unknown***	9311	9311	Nipp allele is unique			
	Os05g25490.1	***ACC7***	Kitaake	Nipp	1 idl:Kitaake, others same as Nipp		No dispute	No dispute
	Os05g25640.1	***C4H***	Kitaake	Nipp	1 idl:Kitaake, others same as Nipp		No dispute	No dispute
	Os05g38120.1	***SH5***	Nipp	Nipp			No dispute	No dispute
	Os05g44210.1	***T6PS***	Nipp	Nipp			No dispute	No dispute
**6**	Os06g04280.1	***EPSPS***	Nipp	Nipp			No dispute	No dispute
	Os06g06320.1	***Hd3a***	Nipp	Kitaake		1 nons:Kitaake, others same as Nipp	No dispute	No dispute
	Os06g06560.1	***SSY1***	Nipp	Nipp			No dispute	No dispute
	Os06g10340.1	***C1***	Nipp	Kitaake		2 nons:Kitaake, others same as Nipp	No dispute	No dispute
	Os06g12230.1	***TCP19***	Kitaake	Nipp	1 idl:Kitaake, others same as Nipp		No dispute	No dispute
	Os06g16370.1	***Hd1***	Kitaake	Kitaake	1 idl:Kitaake, others same as Nipp	1 nons:Kitaake, others same as Nipp	No dispute	No dispute
	Os06g18790.1	***3GT***	Nipp	Nipp			No dispute	No dispute
	Os06g37180.1	***vATPB1***	Kitaake	Nipp			No dispute	One 5' later idl(specific to Nipp)is not in Kitaake.
	Os06g45540.1	***GL6***	Nipp	Nipp			No dispute	No dispute
**7**	Os07g05900.1	***PROG1***	Kitaake	Nipp	1 idl:Kitaake=Or, others same as Nipp		1 idl of 2 5'mutations not in Kitaake	No dispute
	Os07g08420.1	***bZIP58***	Nipp	Nipp			No dispute	No dispute
	Os07g11030.1	***Rc***	Kitaake	Nipp	1 idl:Kitaake=Or, others same as Nipp		No dispute	No dispute
	Os07g13170.1	***SSH1***	Nipp	Nipp			No dispute	No dispute
	Os07g15770.1	***Ghd7***	Nipp	Kitaake		1 nons:early stop codon in Kitaake, others same as Nipp	No dispute	No dispute
	Os07g39700.1	***SDR4***	Kitaake	Nipp	mixed sites of I & J, no specific site		No dispute	No dispute
	Os07g41240.1	***BG2***	Kitaake	Nipp	1 idl of Nipp not in Kitaake, others same as Nipp		No dispute	One 5' later idl(specific to Nipp)is not in Kitaake.
	Os07g42960.1	***DAHPSp***	Nipp	Nipp			No dispute	No dispute
	Os07g45090.1	***NADH***	Nipp	Nipp			No dispute	No dispute
	Os07g49460.1	***PRR37***	Nipp	Kitaake		1 nons (C->G) of Nipp not in Kitaake & 1 nons(T->C) of Kitaake not in Nipp	No dispute	One later nons(specific to Nipp)is not in Kitaake.
**8**	Os08g07740.1	***Hd5(Gdh8)***	Kitaake	Nipp	1 idl:Kitaake, others same as Nipp		No dispute	No dispute
	Os08g07740.1	***SSY3***	Nipp	Nipp			No dispute	No dispute
	Os08g09230.2	***APS1***	Nipp	Nipp			No dispute	No dispute
	Os08g34290.1	***CM4***	Nipp	Nipp			No dispute	No dispute
	Os08g37890.1	***RAE2***	Nipp	Nipp & 9311			No dispute	No dispute
	Os08g39890.1	***IPA1***	Kitaake	Kitaake	1 idl:Kitaake, others same as Nipp	1 nons:Kitaake, others same as Nipp	No dispute	No dispute
	Os08g41940.1	***SPL16***	Nipp	Nipp			No dispute	No dispute
**9**	Os09g26890.1	***unknown***	Nipp	Nipp			No dispute	No dispute
	Os09g26900.1	***unknown***	Nipp	Nipp			No dispute	No dispute
	Os09g26999.1	***DEP1***	Nipp	Nipp			No dispute	No dispute
	Os09g29070.1	***PGI***	Nipp	Nipp			No dispute	No dispute
	Os09g29820.1	***bZIP73***	Nipp	Nipp			No dispute	No dispute
	Os09g36220.1	***PRR95***	Kitaake	Nipp	1 subs:Kitaake, others same as Nipp		No dispute	No dispute
	Os09g36800.1	***DHQS***	Nipp	Kitaake			No dispute	One later syn(specific to Nipp)is not in Kitaake.
**10**	Os10g11140.2	***PGMp***	Nipp	Nipp			No dispute	No dispute
	Os10g17260.1	***F3'H***	Nipp	Nipp			No dispute	No dispute
	Os10g32600.1	***Ehd1***	Kitaake	Nipp	Mixed sites of I & J, 1 idl:Kitaake		No dispute	No dispute
	Os10g41480.1	***DAHPS2***	Kitaake	Nipp	1 idl:not in Kitaake, others same as Nipp		No dispute	One later 5' idl(specific to Nipp)is not in Kitaake.
	Os10g42430.1	***MYC2***	Nipp	Nipp			No dispute	No dispute
	Os10g40600.1	***NRT1.1B***	Nipp	Nipp			No dispute	No dispute
**11**	Os11g05110.2	***PK1***	Nipp	Nipp			No dispute	No dispute
	Os11g07910.1	***unknown***	Nipp	Nipp			No dispute	No dispute
	Os11g10510.1	***ADH2***	Nipp	Nipp			No dispute	No dispute
	Os11g29350.2	***unknown***	Nipp	Nipp			No dispute	No dispute
	Os11g29400.1	***unknown***	Kitaake	Nipp	1 idl:Kitaake, others same as Nipp		No dispute	No dispute
	Os11g32650.1	***CHS***	Nipp	Nipp			No dispute	No dispute
**12**	Os12g01760.1	***unknown***	Nipp	Nipp			No dispute	No dispute
	Os12g34860.1	***unknown***	Nipp	Nipp			No dispute	No dispute
	Os12g34874.1	***SDH2(DHQD2)***	Nipp	Nipp			No dispute	No dispute
	Os12g34920.1	***unknown***	Nipp	Nipp			No dispute	No dispute
	Os12g38920.1	***unknown***	Nipp	Nipp			No dispute	No dispute
	Os12g38900.1	***CM2***	Nipp	Nipp			No dispute	No dispute
	Non-Nipponbare regions		**27**	**16**				
	Disputed later mutations		7	6				
	Disputed early mutations		1	0			.	

[a] Nipp is for Nipponbare
[b] Mut stands for mutation, subs for substitution, and idl is for indel. Synonymous substitution is abbreviated as syn and nonsynonymus as nons.
[c] The graphic displays are in Additional file 2: Fig. S1.
