# Supplementary material for: Early Domestication History of Asian Rice Revealed by Mutations and Genome-Wide Analysis of Gene Genealogies
Source: Rice (N Y). 2022 Feb 15;15:11. doi: 10.1186/s12284-022-00556-6 (PMC8847465; doi:10.1186/s12284-022-00556-6)
Supplement: Supplementary file 16 — Additional file 16: Table S12. List of primers used in this study. [file 12284_2022_556_MOESM16_ESM.pdf]

Additional file 16

Supplemental Table 12. List of primers used in this study.

Experiment	Gene	Primer name	Sequence (5’- 3’)[a]
Genomic survey	*OsSSY3*	SSY3-e3f	GTTCGGAGACATGATCCACTACAT
		SSY3-e3r	TGCAACCACCATGCATCCAGATCT
	OsAPS1	APS1-e2f	ACTCTGCCTCTCTGAACCGTC
		APS1-e3r	CATGAGGCCGAATGCAGTTGC
	OsCKX2	CKX2-1f	TCCATCCACGCTGCTAGTGCTG
		CKX2-e3r	TGGCCACGGGACAGGATG
	OsPGMp	PGMp-e17	TCTAGCTTGGCTGTCCATACTTGCA
		PGMp-e20	GGAAGATAATCCTAGATCCATCGGTG
C1 survey	*OsC1*	C1p-1Kf	GATATGAGCATTATCCGTCCG
		C1-30r	AGAGCTTGCTGCGCAAAGGA
Mutation test	$OsDFR_{pro}$	OsDFRpro0921-F	caagctaattcgagctcggtaccGACAGGACTTCTATAGATTATAAA
		OsDFRpro0921-R	gtcttccatggatccgtcgacGGCGTACCGTGCGTGATC
	OrMYB3	Orlink1r1	GGTGGTGGTGGTGGTGGTGGTGGTGGTGGTGGTGGTCACCG
		Orlink1r2	TCCTGCTGCTGGTGGTGGTGGTGGTGG
		Orlink2f	CACCACCAGCAGCAGGAGGAGGAGGTGC
		OsTT2-163-SalIF	acgtcgacgATGGGGAGGAAGCCGTGC
		OsTT2-163-EcoRI_R:	acgccggaattcTCAGAGGAGCCATGGCGC
	OnMYB3	OnTT2cag-f	CAGCAGCAGCAGCAGCAAGAGC
		OnTT2cag-r	CTGCTGCTGCTGCTGCTGCACCG
		OnTT2tlnk-r	CGCCGCCGAGCTCATAGAC
		OnTT2tlnk-f	GTCTATGAGCTCGGCGGCG
		Onlink1r	TCCTGCTGCTGCTGGTGGTGGTGGTGGTCACC
		Onlink2	CACCAGCAGCAGCAGGAGGAGGAGGTGC
		OnTT2-163TF-F	tggagaggacagcccaagcttATGGGGAGGAAGCCGTGC
		OnTT2-163TF-R	tccgtcgacctgcatctagaTCAGAGGAGCCATGGCGCCAAC
	$OsCHS_{pro}$	$OsCHS_{pro}$-EcoRIF	acgccggaattcGTGCATATATACCAACTAAATAGTCATC
		$OsCHS_{pro}$-MluIR	acgccgACGCGTCTCTCTCGACTAATTCACCAG
	$DFR_{pro\text{-}short}$	OsDFRp370f	CAAGCTAATTCGAGCTCGGTACCTAAGAAAATTATGAAAAAAAATTAAAAAAACAAG
		OrDFRp369f	CAAGCTAATTCGAGCTCGGTACCTAAGAAAATTATGAAAAAAATTAAAAAGACAAG
	Hd3a	OsHd3alinker1	CACCAAGTGCAAGGTAGCGCAAAACTATAAACCTTCAGGGTTTTTTGCAAGATCG
		Hd3alinker2	CTTCCATGGATCCGTCGACCGATCTTGCAAAAAACCCTGAAGG
		orHd3alinker1	CACCAAGTGCAAGGTAGCGCAAAACTATATACCTTCAGGGTTTTTTGCAAGATCG

[a] sites in lower case indicate restriction enzyme cutting area.
